# Minimal-actuation feather star–inspired soft swimmers for multimodal 3D maneuverability

**DOI:** 10.1126/sciadv.aeg9211

**Published:** 2026-10-07

**Authors:** Haitao Qing, Yuanhang Zhu, Jiacheng Guo, Caizhi Zhou, Haoze Sun, Haibo Dong, Daniel Quinn, Jie Yin

**Affiliations:** ^1^ Department of Mechanical and Aerospace Engineering, North Carolina State University, Raleigh, NC 27695, USA.; ^2^ Department of Mechanical and Aerospace Engineering, University of Virginia, Charlottesville, VA 22903, USA.; ^3^ Department of Mechanical Engineering, University of California, Riverside, CA 92521, USA.

## Abstract

Complex, three-dimensional (3D) motions typically require actuator arrays and complex control architectures. Here, we present a feather star–inspired soft robotic swimmer that uses only two pneumatic inputs to produce three distinct and switchable swimming modes: jellyfish-like pulsation, fishlike propulsion, and rotor-like reorientation. The robot owes this ability to mechanical intelligence: It leverages a monostable instability in its flexible arms to convert two control actuation inputs into 3D swimming modes, including ascension and descension, forward and backward swimming, hovering, and rotation. The robot achieves a maximum swimming speed of 1.64 body lengths per second, minimum cost of transport of 17.6, and peak rotation speed of 90° per second. Particle image velocimetry analyses and computational fluid dynamics simulations reveal distinct vortex structures governing thrust generation and/or rotational torque in each swimming mode. The robot’s minimal input yet multimodal output demonstrates how mechanical intelligence can enable adaptive and multifunctional, yet simple and energy-efficient, robotic and biological swimming mechanisms.

## INTRODUCTION

Marine animals exhibit versatility in locomotion, using diverse propulsion strategies to navigate complex and dynamic underwater environments. Creatures such as fish, jellyfish, rays, and cephalopods achieve efficient thrust generation and agile three-dimensional (3D) maneuverability through the coordination of compliant structures and distributed actuation, offering fundamental insights for the design of adaptive soft robotic swimmers (*1*–*7*).

Inspired by these biological systems, recent efforts have developed versatile soft swimming robots composed of compliant structures and driven by pneumatic (*8*), dielectric elastomer (*9*, *10*), and photothermal (*11*) actuators. Jellyfish-inspired designs generate thrust through pulsation (*12*–*17*), whereas fishlike (*18*–*21*) and ray-inspired robots (*22*) swim via undulation or fin flapping. Despite these advances, most soft swimmers remain limited to unimodal locomotion and restricted planar trajectories (*23*, *24*). Achieving fully 3D reorientation typically requires multiple independent actuators and control modules. For example, a tuna-inspired robot (*25*) uses six separate inputs for propulsion, pitch, and buoyancy control, thus increasing structural, actuation, and control complexity and reducing overall efficiency. A persistent challenge therefore lies in realizing multimodal and 3D maneuverable swimming with minimal-actuation complexity in soft swimming robotics.

Among natural swimmers, feather stars (crinoids), also known as sea lilies, provide an elegant and distinct model for multimodal propulsion (Fig. 1A) (*26*). Using multiple flexible, feather-like arms, they coordinate synchronous and metachronal flapping to generate upward thrust similar to jellyfish, hover stably in place, steer forward or backward similar to fish, and reorient in three dimensions through rotor motions. Leveraging this versatile locomotion, a typical feather star with a body length (BL) of ∼350 mm achieves an average vertical swimming speed of 0.15 BL/s by extending its arms laterally during downstroke and a horizontal speed of 0.19 BL/s through differential arm extension (*27*). This distributed yet coordinated actuation yields locomotor versatility emerging from simple repetitive and symmetric structures, a capability largely unexplored in engineered soft robotic systems (*28*).

**Fig. 1.**
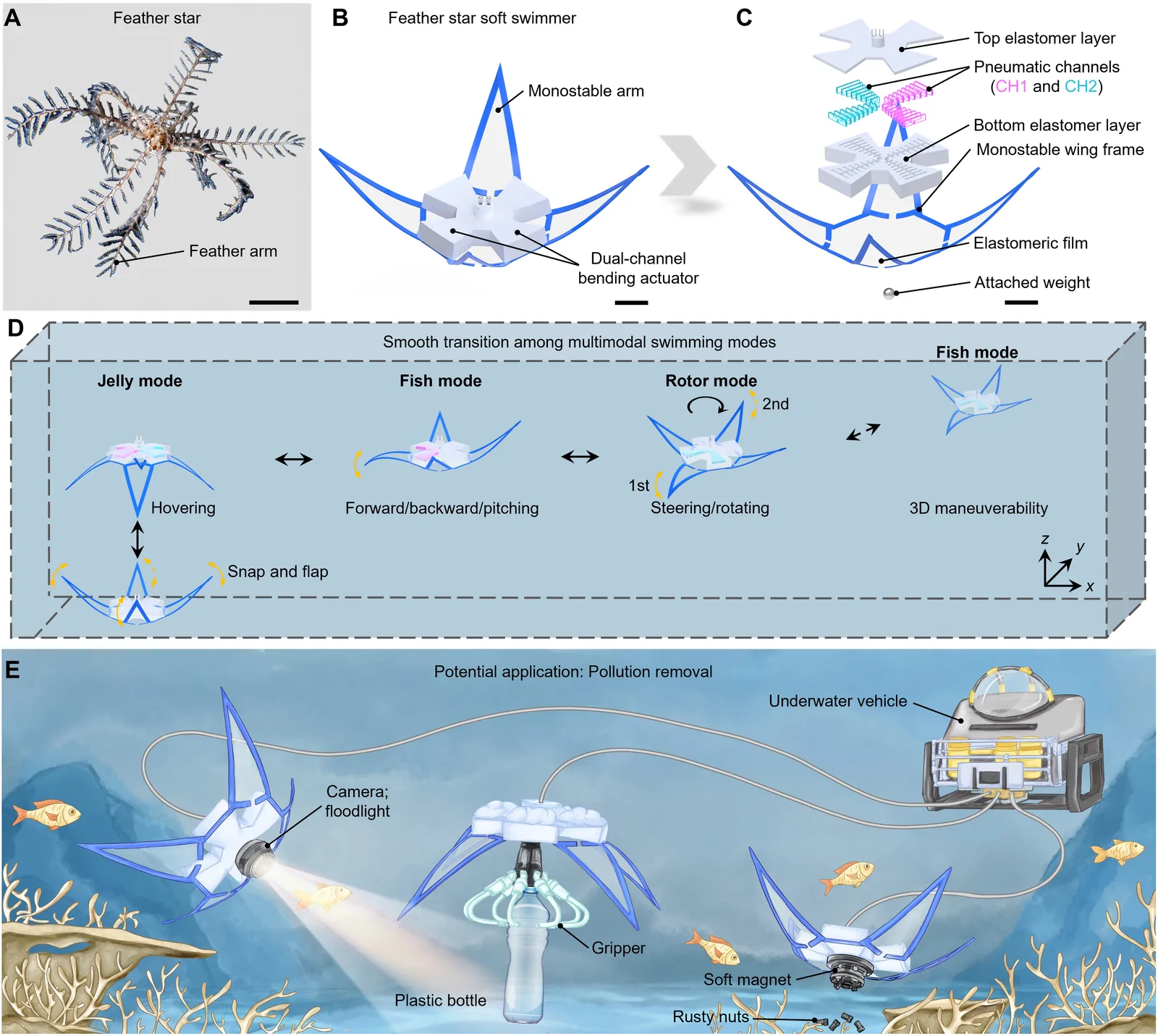
Feather star–inspired soft robot for multimodal and 3D maneuverability. (**A**) A feather star (crinoid) featuring multiple feather-like arms for multimodal propulsion. Scale bar, 50 mm. (**B** and **C**) Schematics of the soft robot in fully assembled (B) and exploded (C) configurations. The robot consists of four symmetric, flexible, monostable ribbon arms joined to a central octagonal monostable frame. Coordinated arm flapping is driven by a dual-channel pneumatic bending actuator that serves as the body and induces rapid snapping motions. Scale bar, 10 mm. (**D**) Schematic illustration of continuous transitions among multimodal swimming modes, including jelly mode, fish mode, and rotor mode, achieved through synchronous and sequential arm actuations. (**E**) Conceptual schematic of the swimmer as a multifunctional platform for underwater applications, including metallic and plastic waste collection and environmental monitoring, enabled by integrating soft magnets, grippers, and miniature underwater cameras.

Here, inspired by feather stars, we present a soft swimming robot that achieves fast, multimodal, and 3D maneuverable locomotion using only two pneumatic actuation inputs. The robot features four symmetric, flexible triangular ribbon arms driven by a dual-channel soft pneumatic bending actuator as the central octagonal body (Fig. 1, B and C). It leverages snapping mechanical intelligence in both the body and individual arms to achieve fast and coordinated flapping motions arising from monostable instabilities in the flexible frame. By modulating the frequency and phase of the two actuation inputs, which govern synchronous and sequential arm motions, the swimmer can transition among three distinct locomotion modes: jelly mode, i.e., jellyfish-like pulsation for vertical ascent, descent, and in-place hovering; fish mode, i.e., fishlike flapping for forward and backward swimming at tunable pitch angles; and rotor mode, i.e., rotor spinning for 3D reorientation (Fig. 1D).

Combined particle image velocimetry (PIV) and computational fluid dynamics (CFD) analyses reveal the hydrodynamic physics underlying its multimodal propulsion. We further demonstrate its potential for underwater tasks, including pollution collection, cooperative multiswimmer operation, and environmental exploration (Fig. 1E). This feather star–inspired design demonstrates that complex, agile, and multifunctional swimming behaviors can emerge from minimal-actuation inputs, establishing a general framework for efficient and adaptable soft swimming robots.

## RESULTS

### Feather star–inspired soft swimming robot design and fabrication

The feather star–inspired soft robot consists of a dual-channel, cross-shaped soft bending actuator integrated with a four-arm ribbon frame that together form the central body and flapping appendages (Fig. 2A and movie S1). The elastomeric actuator contains two separate and symmetric pneumatic channels (CH1 and CH2) that independently control unidirectional bending on the left and right sides (Fig. 1C and figs. S1 and S2). The actuator is first aligned and bonded to an octagonal polyester ribbon frame (Fig. 2Aa and fig. S3). Bonding the tips of four pairs of parallel ribbons introduces prestored elastic energy, causing the ribbons to deform spontaneously into four precurved triangular arms (arm length *AL* = 45 mm). This configuration creates a multistable ribbon structure with 16 discrete stable states (Fig. 2Ab and figs. S3 to S6), evidenced by the negative stiffness and forces in the force-displacement curves (Fig. 2B, fig. S7, and movie S2) and the two local minima in the energy landscape (Fig. 2C). Each arm can snap between two stable configurations corresponding to the upstroke and downstroke states, reflecting the intrinsic bistability of the structure. In principle, a single bistable arm requires two independent actuation inputs to drive a complete flapping cycle (*2*). Therefore, independent control of flapping in all four arms would necessitate at least eight separate actuation inputs, substantially increasing the mechanical and control complexity of the system.

**Fig. 2.**
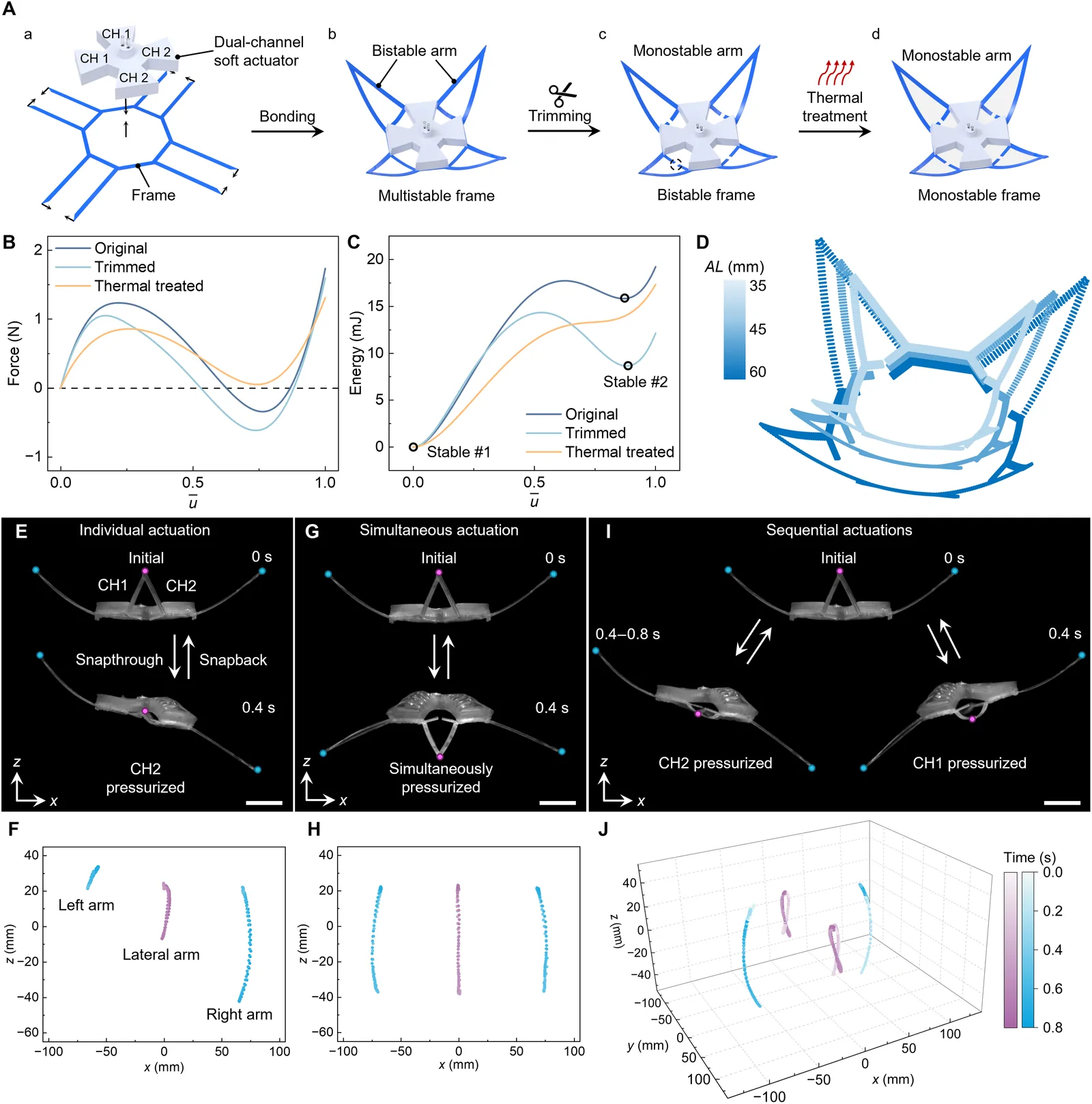
Fabrication, mechanical characterization, and multimodal actuation. (**A**) Fabrication process of the soft robot through sequential bonding, trimming, and thermal treatment to generate a monostable body frame and arms capable of multimodal actuation driven by dual-channel pneumatic inputs. (**B** and **C**) Force-displacement curves (B) and corresponding energy landscapes (C) of the original, trimmed, and thermally treated architectures with arm length *AL* = 45 mm, showing the transition from bistability to monostability. (**D**) Analytical predictions of ribbon-frame and arm geometries with varying arm lengths (*AL* = 35, 45, and 60 mm). (**E** to **J**) High-speed images and corresponding arm-tip trajectories during representative downstroke cycle (snapthrough) and upstroke (snapback) cycles, demonstrating snapping amplified arm-flapping motions under different actuation modes: single-channel [(E) and (F)], simultaneous dual-channel [(G) and (H)], and sequential dual-channel [(I) and (J)] pressurization and depressurization. Scale bars, 20 mm.

To overcome this limitation, we strategically harness structural monostability, achieved through selective trimming and thermal treatment, to simplify the actuation architecture, enabling coordinated multimodal flapping with only two pneumatic inputs. In the first step, the central buckled beams in each arm were trimmed at their midpoints to release stored elastic energy, converting the bistable arms into monostable elements (Fig. 2Ac). Thus, each arm required only a single actuation input to complete a cyclic flapping motion: Inflation drives the snapthrough downstroke, while deflation triggers a spontaneous snapback upstroke powered by the elastic restoring force (*3*). Although this modification removed bistability from the arms, the central octagonal frame remained bistable due to its retained high residual stress (Fig. 2, B and C) and would still require additional actuation inputs for coordinated operation (fig. S8) (*2*). To address this, we applied a subsequent thermal treatment to further relax this stress (*3*), rendering the frame fully monostable with negative stiffness and positive force and a single energy minimum (Fig. 2, Ad, B, and C, and figs. S9 and S10). This transformation preserves the overall structural integrity while simplifying its energy landscape. Varying the arm lengths preserves this monostability in both the central frame and the arms, with 3D geometries in close agreement with analytical predictions, supported by the 3D-scanned models (Fig. 2D and fig. S11).

We explore how this structure-actuation codesign enables multimodal arm motions using only two control channels. Figure 2 (E to J) shows the time-lapse sequences and tip trajectories of the stationary robot during a single actuation cycle under water (fig. S12 and movie S3). When a single channel is inflated (e.g., CH2, single-channel actuation frequency *f*_CH2_ = 2.5 Hz; Fig. 2E and fig. S13), the right side of the body bends downward, driving the right arm through a rapid snapthrough within ∼26 ms and producing a powerful downstroke, followed by an equally fast snapback recovery upon deflation. This upstroke motion is powered by the elastic restoring force of the frame, enabled by the arm’s monostable characteristics. The resulting flapping reaches a large amplitude of ∼55° in the actuated right arm, while the two lateral arms undergo moderate chordwise rotation (∼25°) without snapping, and the unactuated left arm lifts slightly (Fig. 2F). In the fully actuated state, the body reaches a pitch angle θ of ∼15°, and the head-trail deflection angle increases to ∼30°.

When both channels are actuated simultaneously, the body bends symmetrically downward, driving all four arms to snap through simultaneously and generate a synchronized downstroke (double-channel actuation frequency *f*_double_ = 2.5 Hz; Fig. 2G and fig. S13). Upon deflation, the arms snap back to their initial state, yielding a large-amplitude flapping motion across all arms (Fig. 2H). This fully coordinated behavior arises from the monostable nature of the integrated body-arm structure achieved through trimming and thermal treatment, which allows fast, reversible deformation under cyclic pneumatic actuation. Sequential alternate activation of the two channels (CH1↔CH2) produces out-of-phase bending on opposite sides (sequential actuation frequency *f*_CH1↔CH2_ = 1.25 Hz; Fig. 2I and fig. S14). Two monostable arms alternate their downstroke-upstroke cycles in a mirror-symmetric fashion, while the lateral arms exhibit slight asymmetry in their flapping trajectories (Fig. 2J and fig. S15).

These distinct actuation patterns correspond to three primary swimming gaits: (i) jelly mode, in which synchronized four-arm flapping generates jellyfish-like vertical pulsation; (ii) fish mode, in which single-arm actuation produces fish- or dolphin-like propulsion through tail beating with pectoral-fin stabilization; and (iii) rotor mode, in which alternating actuation induces spinning for reorientation. Notably, these multimodal behaviors are achieved using only two pneumatic channels rather than four independent actuation inputs for each arm. This reduction is not the result of arbitrary coupling but arises from the structure-actuation codesign that preserves task-relevant df while organizing them into distinct locomotion modes.

Through asymmetric pairing and elastic coupling, two actuation inputs generate coordinated synchronous and sequential motions that give rise to the three gaits. In this way, control is embedded within the mechanical architecture, allowing complex behaviors to emerge from simple inputs. We refer to this property as mechanical intelligence, whereby geometry asymmetry, elastic coupling, and phase-modulated actuation collectively perform functions that would otherwise require additional actuators and more complex control schemes.

### Jelly and fish modes

Next, we investigate the robot’s multimodal underwater swimming performances, such as jelly mode (Fig. 3, A to F) and fish mode (Fig. 3, G to K). When both channels are actuated simultaneously during cyclic inflation and deflation, the robot operates in jelly mode, flapping all four arms synchronously via snapping-enabled strokes (Fig. 3, A and B, and fig. S16). This mode achieves fast vertical ascent (Fig. 3C and fig. S17) and slow descent (Fig. 3D and fig. S18). Figure 3B shows the time-lapse images of an ascending soft robot with a speed of ∼40 mm/s at an actuation frequency *f*_double_ = 2.5 Hz (movie S4). As *f*_double_ increases from 1 to 4 Hz, the ascending speed rises nearly linearly from 0 to ∼90 mm/s (Fig. 3C). The highest relative swimming speed is about ∼1.64 BL/s based on the body diameter of 55 mm, which is superior to most jellyfish-inspired soft robots in the range of 0.1 to 0.4 BL/s (*4*, *6*, *23*) (table S1). Below *f*_double_ = 1 Hz, the swimmer descends; and when the actuation is turned off (0 Hz), it sinks freely at ∼32 mm/s due to its mass density slightly exceeding that of water. As *f*_double_ increases from 0 to 0.75 Hz, the descending speed decreases linearly from ∼32 to ∼15 mm/s (Fig. 3D and fig. S18). Vertical motion of the swimmer is regulated through pneumatic actuation, as the actuation frequency modulates the cycle-averaged inflation time and thereby changes the cycle-averaged inflated volume and buoyancy while also altering the flapping-induced hydrodynamic lift.

**Fig. 3.**
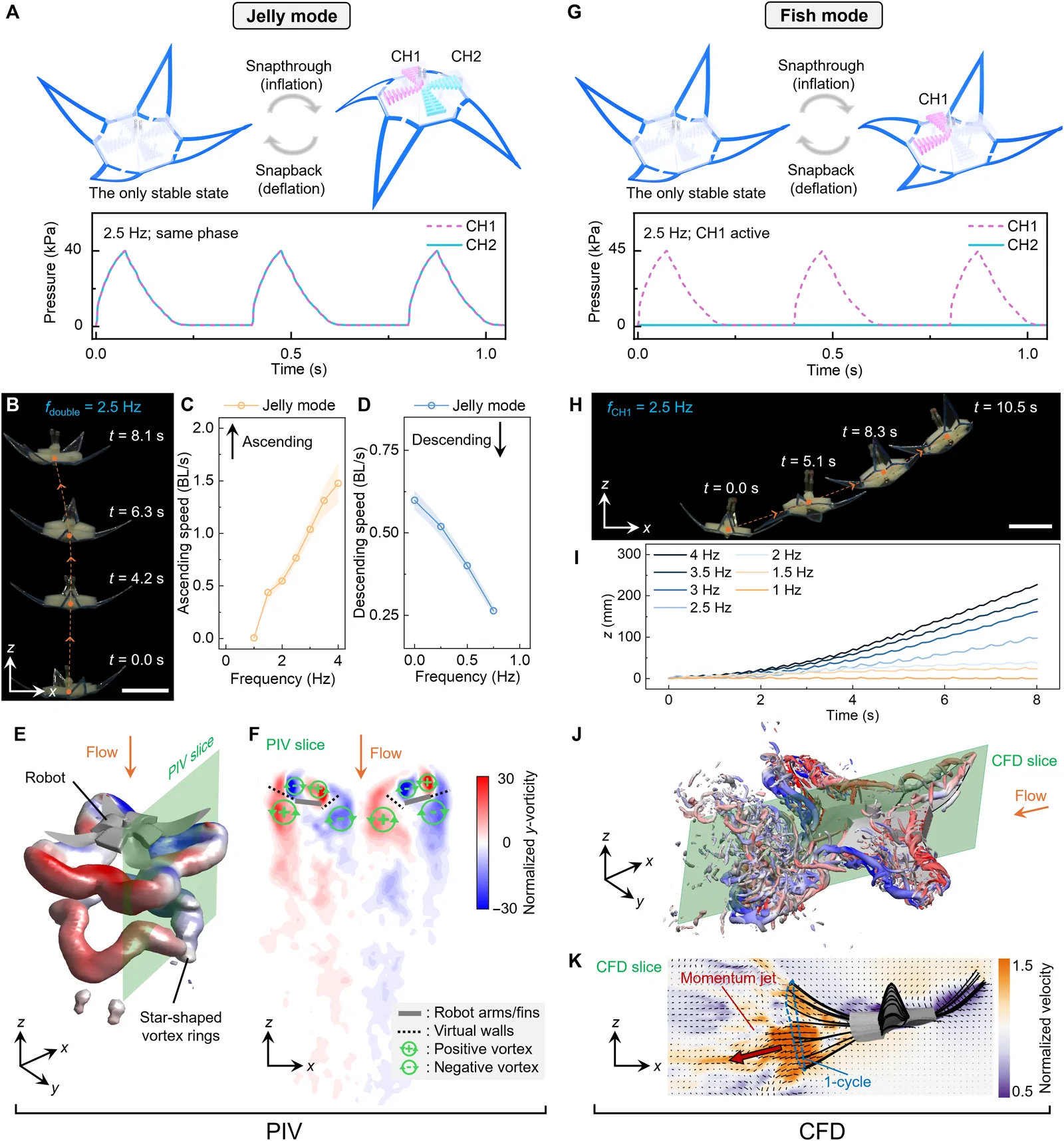
Jelly mode and fish mode. (**A** to **F**) Characterization of jelly mode: Schematic of simultaneous dual-channel actuation and corresponding pressure-time signals (A), experimental vertical ascending motion (B), measured ascending (C) and descending (D) speeds versus actuation frequency, and PIV analyses [(E) and (F)]. (B) Time-lapse front-view images showing vertical ascent in jelly mode of the soft robot (*AL* = 45 mm) under a frequency *f* of 2.5 Hz. Scale bar, 50 mm. (E) A train of star-shaped vortex rings generated during jelly-mode actuation, measured using PIV. Red indicates positive counterclockwise vortices, and blue indicates negative clockwise vortices. (F) A slice of the 3D PIV flow field showing the vortex dynamics and propulsion mechanism associated with jelly mode. (**G** to **K**) Characterization of fish mode: schematic of single-channel actuation and pressure-time signals (G), experimental rightward steering motion (H), swimming trajectories versus time (*t* = 0 to 8 s) at actuation frequencies from 1 to 4 Hz (I), and corresponding CFD simulation [(J) and (K)]. (H) Time-lapse front-view images showing the steering behavior in fish mode of the soft robot (*AL* = 45 mm) under a frequency *f* of 2.5 Hz. Scale bar, 50 mm. (J) 3D vortex dynamics in fish mode obtained from CFD in perspective view. (K) The CFD simulation of fish mode, showing the streamwise momentum on sagittal cross sections of the flow field intersecting the main fin, a quarter span from the fin tip. Scale bar, 20 mm.

When only one channel is actuated (Fig. 3G and figs. S16 and S19), the swimmer transits to a fish mode, flapping one arm as a tail to steer rightward (CH1 on) or leftward (CH2 on; Fig. 3, G to K). Figure 3H shows the time-lapse images of a rightward steering soft robot in fish mode (movie S5). As the single-channel actuation frequency *f*_CH1_ increases from 1 to 4 Hz, the swimmer progresses from bottom crawling to horizontal swimming and lastly to pitched swimming (fig. S19). Between 1 Hz < *f*_CH1_ ≤ 2 Hz, it exhibits steady cruising at intermediate depths, while beyond 2 Hz, it adopts a pitched trajectory with a high pitch angle of ∼48° and a steering speed of 44 mm/s (0.8 BL/s) at *f*_CH1_ = 4 Hz.

For jelly mode, the vertical propulsion is governed by a drag-based, pulsatile mechanism that is hydrodynamically analogous to medusan swimming, although the robot consists of four discrete fins rather than a continuous bell (*29*–*31*). The synchronous snapthrough of all four fins produces a rapid contraction of the propulsive surface, accelerating fluid downward and generating a train of vortex rings that interact constructively with the body during successive cycles, a phenomenon known to associated with higher wake efficiency (Fig. 3E and figs. S20 and S21) (*32*). The discrete fin geometry gives rise to the starlike shape of the vortex rings while maintaining a largely symmetric momentum distribution that supports stable vertical ascent (movie S6). As in biological jellyfish, vortices of opposite sign generated during the preceding fin relaxation persist near the propulsor and form a dynamically generated “virtual wall,” enhancing pressure beneath the body and increasing thrust during the subsequent contraction (Fig. 3F) (*30*). This vortex-vortex interaction enables the robot to exploit passive energy recapture, whereby kinetic energy stored in the vortex generated in the previous cycle continues to contribute to upward motion during the coast phase without additional actuation (*33*, *34*). Because of snapping, the jelly mode naturally operates in a burst-and-coast regime, featuring high propulsive efficiency by extending the contribution of each actuation cycle (see the actuation time lapse of Fig. 3A).

For fish mode, the propulsion mechanism transitions to a lift-based regime reminiscent of fish swimming (*35*), where lateral propulsion is achieved through the asymmetric snapping of the fins enabled by CH1. The inflation and deflation of CH1 leads to the large-amplitude flapping of the posterior fin. The rapid flapping motion produces large downstream flow momentum, as shown in Fig. 3J, figs. S22 and S23, and movie S7. This large downstream jet helps propel the swimmer forward (*36*). There is also a slight downward tilt of the jet that is mainly responsible for the ascending trajectory of the swimmer in fish mode. At the same time, the side fins undergo moderate flapping similar to our previous design (*3*), which had a propulsion mechanism linked to manta ray. Here, similar bifurcating jets can be seen being produced by the side fins (Fig. 3K) corresponding to the shedding of vortex pairs from the leading and trailing edges (Fig. 3K). These secondary jets also contribute to force balance and body stabilization, consistent with pectoral fin hydrodynamics in fish (*37*). Together, the posterior fin and side fins form a distributed propulsion system, where thrust is generated primarily through momentum redirection rather than bulk fluid displacement. This mode therefore contrasts with jelly mode (*38*).

### Rotor mode

When the two channels are actuated sequentially with a phase shift of ϕ = π (Fig. 4A), the soft swimmer exhibits a rotor mode (Fig. 4, B to D). Rotor mode occurs within a phase range of π/2 < ϕ < 3π/2, and the rotation direction is governed not by ϕ but by the bonding chirality of the arms during fabrication, where clockwise bonding of the ribbon pairs produces clockwise rotation and counterclockwise bonding reverses the direction (Fig. 4B). Starting from the bottom, it continuously rotates clockwise while ascending vertically at an actuation frequency *f*_CH1↔CH2_ = 1.52 Hz, as shown in the front and top views (Fig. 4C, fig. S24, and movie S8). The rotation speed reaches ∼79°/s, while the simultaneous average ascending speed is 37.5 mm/s (∼0.68 BL/s). The tip trajectory of an arm follows a helical path, forming a clockwise spiral during ascent (Fig. 4D). The angular velocity ω can be tuned by the actuation frequency: As *f*_CH1↔CH2_ increases from 1.22 to 1.52 Hz, ω increases nonlinearly from ∼0.9 to 1.25 rad/s (Fig. 4E), accompanied by a concurrent increase in ascending speed. At *f*_CH1↔CH2_ ≈ 1.2 Hz, the swimmer achieves near-neutral buoyancy, enabling stable hovering in rotor mode.

**Fig. 4.**
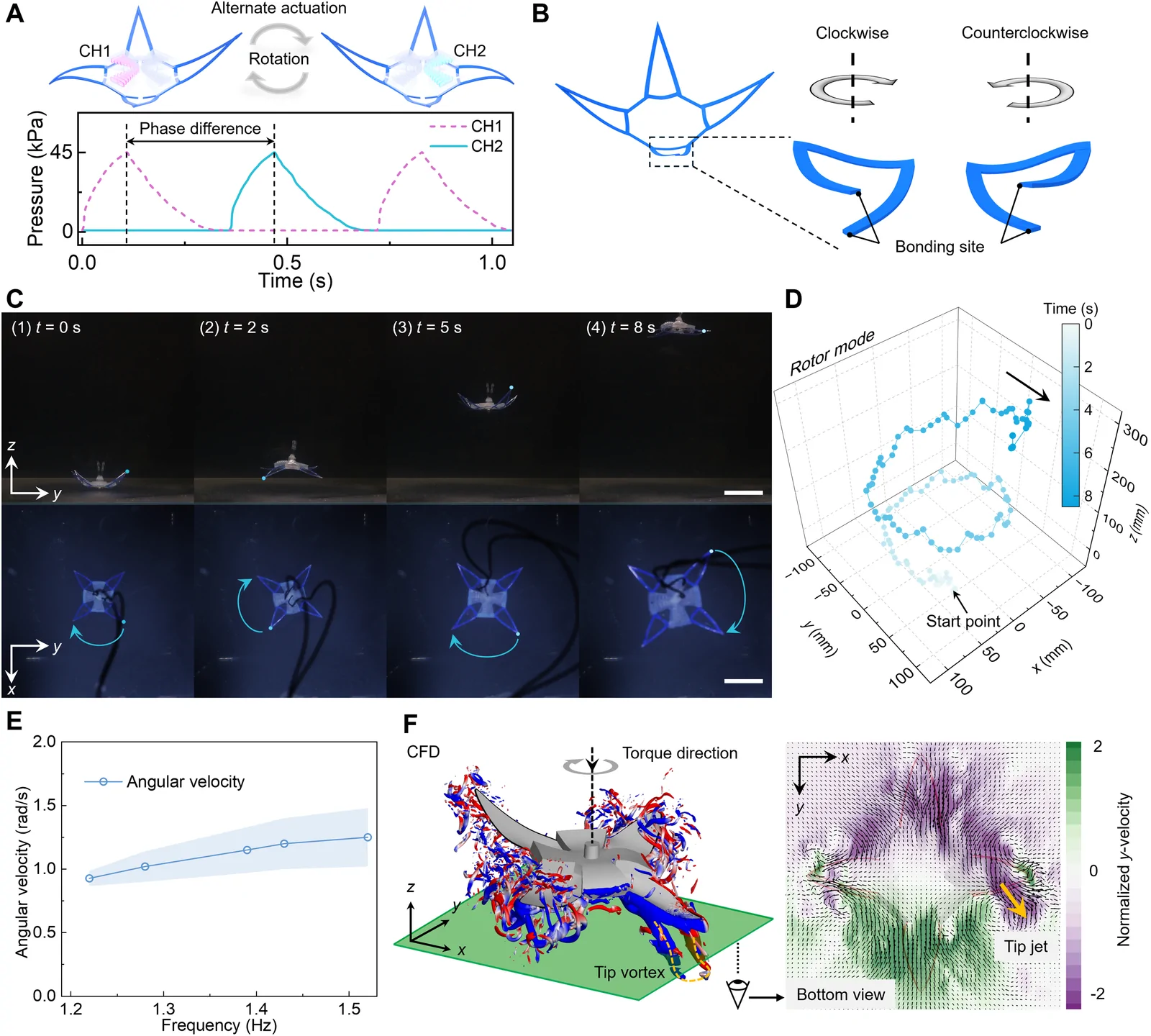
Rotor mode. (**A**) Characterization of rotor mode: schematic of sequential dual-channel actuation with a phase shift of π and corresponding color-coded pressure-time signals (A). (**B**) Schematics showing bonding chirality of the ribbon pairs that determines the rotation direction. (**C** and **D**) Time-lapse front- and top-view images showing clockwise rotational ascent (*AL* = 45 mm, 1.52 Hz) (C), and helical arm-tip trajectory (D). Scale bars, 50 mm. (**E**) Angular velocity versus actuation frequency. (**F**) CFD simulation results validating the rotational dynamics, showing the 3D vortex structure at the end of the snapthrough and the cycle-average lateral momentum distribution near the fin tip.

For rotor mode, chirality-induced asymmetric vortex shedding makes the robot rotate. As either CH1 or CH2 inflates and the actuated fin snaps through, strong 3D vortex features are produced by the fin tip (Fig. 4F, fig. S25A, and movie S9). Because of the clockwise chirality imposed by the ribbon bonding, the fin tip is slightly tilted during snapthrough, and the resulting tip vortices produce a jet with a strong lateral component. When averaged over a cycle of motion, the flow field underneath the fin tip shows a strong lateral jet (Fig. 4F). This leads to a lateral force applied to the fin tip in the direction that is opposite of the jet and thus a torque around the center of the swimmer in the clockwise direction. At the same time, the 3D vortex structures also impart a downward momentum component to the surrounding fluid similar to jelly mode, resulting in a net vertical force and a gradual ascent.

We note that, although a lateral force component arising from chirality-induced tip vortices is also present in fish mode, this contribution is small relative to the dominant axial thrust (fig. S25B), and no appreciable sustained lateral drift is observed experimentally. In fish mode, single-channel actuation primarily generates axial thrust for directional translation. In contrast, in rotor mode, alternating-channel actuation leads to partial cancelation of axial force components while producing a net rotational moment that drives continuous spinning motion. Moreover, despite the presence of similar bonding chirality, no rotor-like motion is observed in our previous two-wing swimmer (*3*). In that configuration, the extended symmetric trailing films promote more symmetric vortex attachment and wake development, thereby suppressing the asymmetric tip-vortex shedding required for rotation. By contrast, the feather star–inspired architecture enables such asymmetric vortex dynamics to emerge and be effectively harnessed for rotational swimming.

### Continuous transitions for 2D and 3D maneuverability

Building on these three modes, we next explore their continuous transitions to enable 2D and 3D maneuverability. Here, “continuous transition” refers to switching between distinct locomotion modes through smooth modulation of actuation inputs (frequency and phase) without discrete structural reconfiguration or interruption of motion. This capability is critical for achieving agile and robust 3D maneuverability in complex underwater environments, as it allows real-time adaptation of propulsion and orientation using low-dimensional control inputs. Figure 5 (A and B) demonstrates the robot’s 2D multimodal maneuverability through continuous transitions between jelly and fish modes (movie S10). A Π-shaped trajectory illustrates continuous vertical ascent, leftward steering, hovering, and descent in 1 cycle (Fig. 5A). The robot ascends in jelly mode at *f*_double_ = 3 Hz, hovers at *f*_double_ = 1 Hz for ∼4 s, steers leftward in fish mode by actuating CH2 (*f*_CH2_ = 3 Hz), hovers again, and lastly descends at *f*_double_ = 0.5 Hz in jelly mode. Notably, at *f*_double_ = 1 Hz, the swimmer transitions into a hovering mode with neutral buoyancy, maintaining its position at desired depth between ascent and descent and stably hovering at arbitrary depths (Fig. 5A and fig. S26). It can also produce steplike trajectories without hovering through continuous bimode transitions (Fig. 5B and fig. S27). These results highlight the robot’s fast, multimodal, and highly maneuverable swimming achieved through only two actuation inputs.

**Fig. 5.**
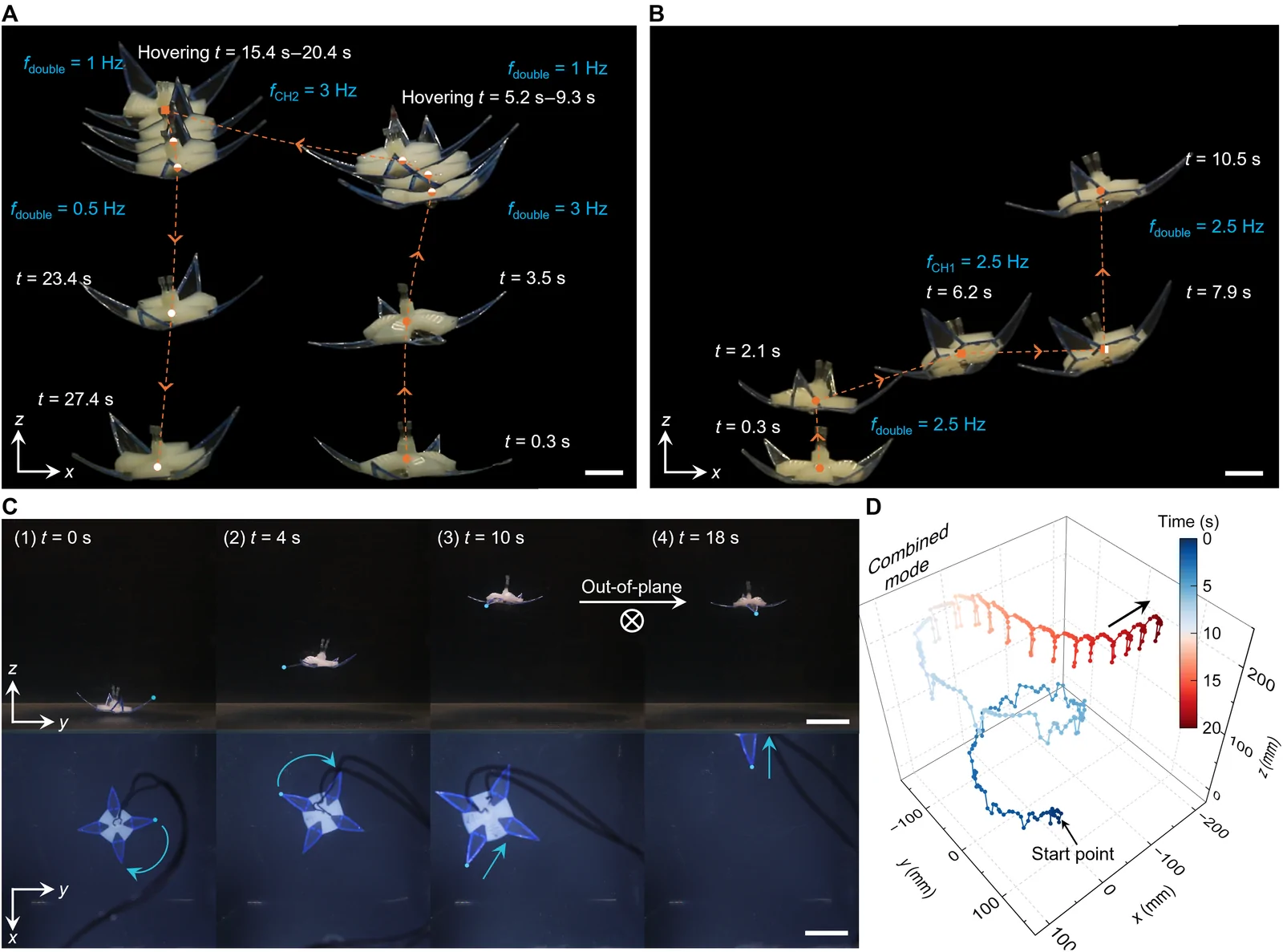
Continuous multimodal transitions enable 3D maneuverability. (**A** and **B**) Demonstrations of Π-shaped (A) and steplike (B) trajectories showing smooth transitions among jelly mode, fish mode, and hovering motion. Scale bars, 20 mm. (**C** and **D**) Time-lapse front- and top-view images showing smooth transitions between rotor and fish modes for 3D swimming (C), and corresponding arm-tip trajectory (D). Scale bars, 50 mm.

Despite its high maneuverability in jelly and fish mode, the swimmer’s motion is primarily confined to in-plane (*xz*/*yz*-plane) trajectories and lacks turning capability for out-of-plane *xy*-plane) navigation (Fig. 5, A and B). Combining rotor mode with the previously demonstrated 2D swimming behaviors allows out-of-plane turning and 3D maneuverability, as shown in the proof-of-concept demonstration (Fig. 5, C and D). The swimmer first ascends in rotor mode and then transitions at *t* = 10 s to the fish mode via single-channel actuation, reorientating its swimming direction out of plane and continuing forward motion with a slight slope (Fig. 5C and movie S11). The recorded arm-tip trajectory confirms 3D maneuverable swimming, achieved through continuous transitions between rotor and fish modes (Fig. 5D). These results highlight a simple yet effective strategy for enabling potentially complex 3D navigation in soft aquatic robots using only two actuation inputs.

### Multifunctional platform for underwater applications

To test whether our propulsion strategy could enable realistic applications, we put the robot through a series of cleanup and inspection trials. Underwater pollutants such as rusted metals and plastics pose serious ecological and health threats, while underwater monitoring demands compact and adaptive platforms capable of carrying sensors or cameras for inspection and exploration. The soft swimmer could serve as a multifunctional platform for diverse underwater tasks. The robot’s compliant body and minimal dual-input control architecture enable integration of task-specific components, such as soft magnets and hooks for manipulation and cameras for inspection, without compromising mobility or stability. Leveraging its multimodal actuation and high maneuverability, the swimmer demonstrates waste collection (Fig. 6, A to E), cooperative manipulation (Fig. 6F), and environmental monitoring in confined aquatic environments (Fig. 6, G and H), highlighting its potential as a versatile soft robotic platform for underwater operations.

**Fig. 6.**
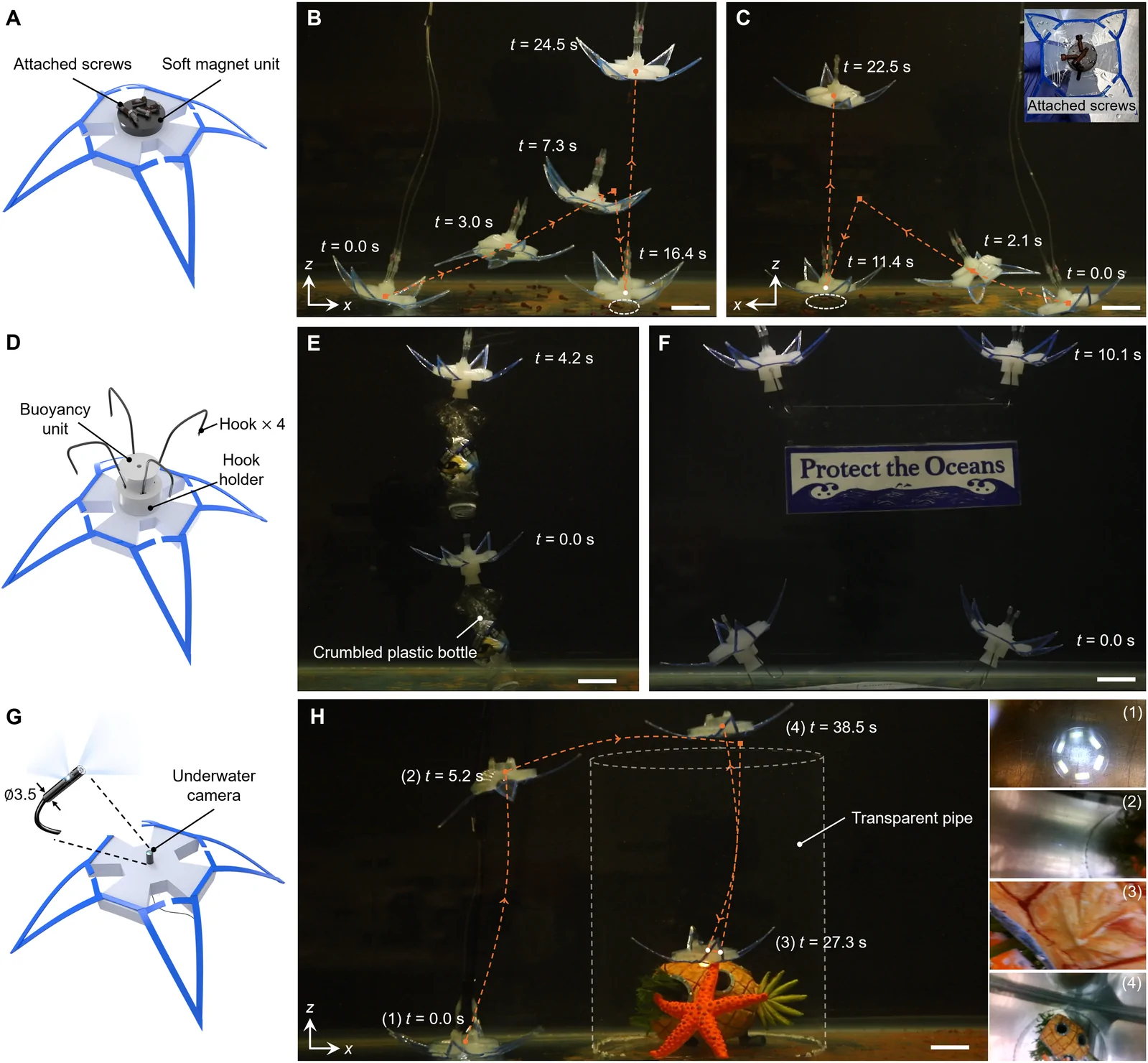
Multifunctional platform for versatile underwater applications enabled by integrated functional components. (**A** to **C**) Metallic waste collection using integrated soft magnetic pads: schematic of screw collection via soft magnets attached beneath the body (A), and time-lapse front-view images showing the retrieval of rusty screws from the water bottom through rightward (B) and leftward (C) steering and jellyfish ascent and decent motions. (**D** to **F**) Plastic waste collection using integrated hooks and cooperative operation: schematic of integrated hooks and buoyancy units (D), and demonstrations of lifting a crumpled plastic bottle with a single swimmer (E) and a larger plastic sticker using two cooperative swimmers (F). (**G** and **H**) Underwater inspection in unstructured environments via an integrated miniature camera (Ø = 3.5 mm): schematic of camera integration at the body center (G), and demonstration of inspecting submerged objects inside a transparent tube through multimodal swimming (H) with corresponding captured images. Scale bars, 50 mm.

We first demonstrate underwater waste collection using the robot’s multimodal swimming and cycle-averaged buoyancy modulation (Fig. 6, A to F). A soft magnet pad is attached beneath the swimmer’s body to capture metallic debris such as rusted screws on the water bottom (Fig. 6A and figs. S28 and S29). Starting from a distance, the robot navigates toward the target in fish mode (*f*_CH1_ = 3 Hz in Fig. 6B; *f*_CH2_ = 3 Hz in Fig. 6C) and then switches to jelly mode at a lower frequency (*f*_double_ = 0.5 Hz) to descend and approach the pile. After several flapping strokes above the target, the magnetic pad successfully collects multiple screws, and the robot ascends using jelly mode at a higher frequency (*f*_double_ = 3 Hz; Fig. 6, B and C, and movie S12). The corresponding vertical displacement over time curve (fig. S29) confirms rapid, controlled ascent and descent, enabling efficient collection within tens of seconds.

To handle plastic waste, such as crumpled bottles and heavy plastic bags, we integrate four small hooks beneath the swimmer for manipulation and cooperative lifting (Fig. 6, D to F, and fig. S30). A single swimmer can lift a hooked lightweight plastic bottle to the surface (Fig. 6E and movie S13), while two cooperating robots jointly lift heavier objects, including a large plastic bag and a “Protect the Oceans” sticker (Fig. 6F and movie S13) for cooperative operations.

Last, to demonstrate underwater environmental exploration and monitoring, a miniature camera (Ø = 3.5 mm) is mounted at the robot’s center (Fig. 6G and fig. S31). In an unstructured underwater setup containing various submerged objects (e.g., an artificial starfish and a pineapple) inside a transparent tube, the robot follows a programmed sequence (Fig. 6H and fig. S32): ascending in jelly mode (*f*_double_ = 2.5 Hz), steering rightward in fish mode (*f*_CH1_ = 2.5 Hz) to move over the open-ended tube, and then descending at a lower frequency (*f*_CH1_ = 0.5 Hz) to enter the confined space and approach objects near the bottom. Between *t* = 27.3 s and 38.5 s, the robot ascends again to exit the tube after capturing detailed underwater imagery (Fig. 6H and movie S14). The corresponding *z*-axis trajectory (fig. S32) highlights its adaptive navigation and multimodal control with minimal-actuation complexity.

### Comparison of energy efficiency and swimming performance

To evaluate the energy efficiency of the soft swimmer, we quantify the cost of transport (CoT) and the Strouhal number (St), with results shown in Fig. 7A and fig. S33, respectively. The CoT is defined as CoT = *E*/(*mgd*), where *E* is the energy consumption, *m* is swimmer mass, *g* is gravitational acceleration, and *d* is the traveled distance. The St is defined as St = *fA*/*U*, where *A* is the flapping amplitude and *U* is the swimming speed.

**Fig. 7.**
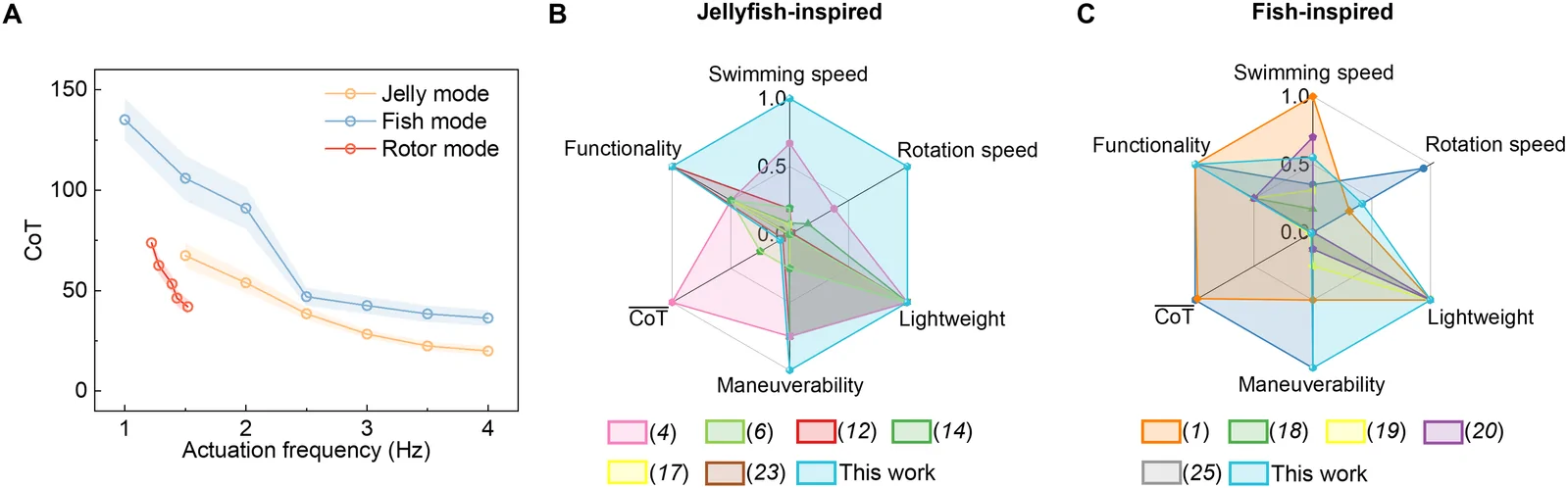
Comparison of swimming performances. (**A**) CoT versus actuation frequency for three locomotion modes of the feather star–inspired swimmer. (**B** and **C**) Radar charts comparing the performance of the present soft swimmer with state-of-the-art jellyfish- (B) and fish-inspired (C) soft swimmer in metrics of swimming speed, rotation speed, $\bar{\text{CoT}}$, maneuverability, lightweight, and functionality.

As the actuation frequency *f* increases, the swimmers achieve higher speeds while exhibiting reduced CoT for all the three modes (Fig. 7A). Overall, fish mode exhibits the highest CoT across the full range of actuation frequency from 1 to 4 Hz, while rotor mode exhibits the lowest CoT at lower frequencies. In jelly mode at *f* = 4 Hz, the swimmer reaches a peak speed of 1.64 BL/s with a minimum CoT of ∼17.6 (Fig. 7A). The corresponding St numbers in jelly mode (*f* > 2.5 Hz) and rotor mode fall within the biologically relevant range of 0.2 < St < 0.4, which is associated with high propulsive efficiency (fig. S33). In contrast, the St numbers in fish mode range from ∼0.5 to ∼0.8, indicating lower propulsive efficiency. This reduced efficiency arises because only a single arm is actuated; however, fish mode enables directional swimming with dynamic depth adjustment, providing functional versatility beyond efficient propulsion.

We next benchmark the swimmer against state-of-the-art soft robotic swimmers in terms of swimming speed, rotation speed, CoT, maneuverability, lightweight design, and functional capability (*1*–*7*, *9*–*23*, *25*, *39*–*44*), as summarized in Fig. 7 (B and C), Table 1, figs. S34 to S37, and tables S1 to S3. Among representative jellyfish- and fish-inspired systems (Fig. 7, B and C, and Table 1), our swimmer achieves a high swimming speed of ∼1.64 BL/s at *f* = 4 Hz in jelly mode, a directional speed of ∼0.8 BL/s in fish mode, and a high rotation speed of ∼90°/s in rotor mode while requiring only two actuation inputs to realize 3D maneuverability. Although several surface-swimming soft robots exhibit lower CoT values, our swimmer maintains a relatively low CoT of ∼17.6 while achieving underwater ascent against gravity, multimodal locomotion, and 3D maneuverability. In addition to locomotion, the platform supports multifunctional capabilities, including waste collection, underwater exploration, and environmental monitoring, whereas most existing systems are primarily designed for propulsion alone (Fig. 7, B and C).

**Table 1.** Summary of performance of representative jellyfish-inspired, fish-inspired, and other bioinspired soft swimming robots and the robot in this work.

		Max. speed (BL/s)	Body length (mm)	Max. speed (mm/s)	Rotation speed (°/s)	Weight (g)	CoT	Maneuverability	Actuation	Power input (*N*)	Functionality
Jellyfish-inspired	Soft robotic jellyfish (*4*)	0.14	210	30	14	380	200.88	2D	Pneumatic	2	Swimming only
	Robojelly (*12*)	0.12	164	20	–	242	2270	1D	SMA	7	Swimming only
	Jellyfish (*23*)	0.31	160	50	1.7	50	24.14	2D	HASEL	2	Waste collection
Fish-inspired	Soft electronic fish (*1*)	1.45	93	135	7.2	42.5	0.43	2D (surface only)	DEA	2	Stealth sailing
	SoFi (*25*)	0.51	470	239.7	22.1	1600	0.42	3D	Hydraulic	6	Underwater exploration
Other bioinspired	Flat swimming robot (*44*)	2.64	45	119	120	1.25	13	2D (surface only)	HASEL	2	Navigation; object translation
	Bistable soft swimmer (*2*)	3.74	22.8	85.3	157	3.1	39	2D (surface only)	Pneumatic	4	Swimming only
	Monostable soft swimmer (*3*)	6.8	23	156.4	26	2.2	23.7	2D	Pneumatic	1	Swimming only
This work		1.64 (J); 0.8 (F); 0.64 (R)	55	90 (J); 44 (F); 35 (R)	90 (R)	14.5	17.6 (J); 32.4 (F); 38.2 (R)	3D	Pneumatic	2	Waste collection; underwater exploration and monitoring

Last, we note that state-of-the-art soft swimmers are typically restricted to a single or a limited set of locomotion modes (*12*–*16*) and often rely on multiple actuators and complex control schemes to achieve even modest multimodal behavior (*6*, *7*, *25*, *42*). Most jellyfish-, fish-, and manta ray–inspired soft robots operate predominately in two dimensions and require two to four actuation inputs to realize in-plane translation and steering, either underwater or at the air-water interface (Table 1). For example, our recent manta ray–inspired bistable flapping-wing swimmer requires two independent actuators and four actuation inputs to achieve propulsion and bidirectional turning, yet its maneuverable remains limited to the water surface (*2*). Replacing bistable wings with monostable ones reduces the actuation inputs to one and enables both surface and underwater swimming at different depths through frequency modulation (*3*). However, despite this reduction in actuation complexity, the motion remains confined to the transverse plane, lacking out-of-plane turning capabilities, similar to the constrained fish mode observed in the present swimmer.

Achieving 3D maneuverability in existing systems generally necessitates six or more independently controlled soft actuators, as demonstrated in tuna- and octopus-inspired swimmers that generate multiple df through distributed actuation (tables S1 to S3) (*25*, *42*). In contrast, the present dual-actuation system leverages the synergy of geometric asymmetry, elastic coupling, and phase-modulated actuation to dynamically reconfigure flapping patterns, enabling multimodal locomotion and 3D maneuverability through low-dimensional inputs and control.

## DISCUSSION

The feather star–inspired soft swimmer illustrates how mechanical intelligence encoded in structural and material designs can enable complex, multifunctional behaviors with minimal control. By coupling a dual-input pneumatic bending actuator with a monostable elastic architecture, the swimmer achieves three distinct locomotion modes: jelly, fish, and rotor modes, as well as their continuous multimodal transitions for 3D maneuverability.

Combined PIV and CFD analyses uncover the hydrodynamic origins of each mode, revealing polygonal vortex-ring formation and periodic jetting in jelly mode, asymmetric wake structures generating directional thrust in fish mode, and counterrotating vortices producing rotational torque in rotor mode. In jelly mode, propulsion is drag based and pulsatile, driven by vortex-ring formation, virtual wall effects, and passive energy recapture, leading to an efficient burst-and-coast strategy. In fish mode, lift-based propulsion arises from periodic vortex shedding that forms a coherent downstream jet sustaining forward motion. In rotor mode, chirality-induced asymmetric vortex shedding generates a lateral jet that drives rotation while imparting a net downward momentum that leads to coupled rotation and vertical motion. Together, these results show how a single soft robot can access distinct hydrodynamic regimes through simple actuation modulation.

Specially, the feather star–inspired swimmer exploits these three modes to achieve 3D maneuverability using only two actuation inputs, illustrating how elastic instabilities and geometric asymmetry enable vertical motion, translation, and rotation to be selectively accessed and largely decoupled within a single morphology through distinct actuation patterns and frequency modulation. Although both the present system and our previous manta ray–inspired flapping-wing swimmer (*3*) leverage elastic instabilities to reduce actuation complexity, the latter is limited to a single propulsion mode with maneuverability confined to 2D transverse plane. This distinction arises from the mechanically intelligent design of the multistable body-arm architecture. In the present design, the monostable arms remain functionally independent despite being mechanically coupled, allowing selective and sequential flapping for fish and rotor modes. In contrast, deformation of the central body, configured as a closed-loop ring frame, induces synchronized flapping of all four arms, enabling jelly mode. By comparison, the H-shaped monostable frame in the previous swimmer (*3*) supports only synchronous flapping of both wings, restricting the system to a single propulsion mode without independent control. These results highlight a scalable design strategy for soft robotic systems, in which complex, multimodal behaviors emerge from embedded mechanical intelligence rather than increased actuation complexity.

Despite its demonstrated multifunctionality, several limitations remain. First, the swimmer relies on tethered pneumatic actuation, which fundamentally limits its autonomy and operational range. In practical deployment, the swimmer would depend on a host remotely operated vehicle (ROV) or autonomous underwater vehicle (AUV) to provide pressurized air, power, and control, effectively functioning as a peripheral platform rather than a fully independent agent. This tethered configuration limits the achievable deployment range, introduces additional hydrodynamic drag that degrades swimming performance, and increases the risk of tether entanglement in cluttered or confined environments such as pipelines and reef structures. In addition, the current platform lacks onboard sensing or closed-loop feedback and thus relies on open-loop control. This limits its ability to adapt to environmental disturbances such as currents and obstacles in natural underwater environments. Furthermore, precise directional control during multimodal transitions remains a challenge. In particular, when switching from sequential dual-channel to single-channel actuation, transition from rotor mode to fish mode is affected by inertial carryover from the preceding rotational motion, making it difficult to fully arrest residual angular momentum and achieve accurate directional steering. This limitation highlights the sensitivity of the system to dynamic coupling between actuation modes.

Future work will focus on addressing these limitations through advances in actuation, control, and system integration. Integrating onboard soft sensing and closed-loop feedback will be essential for enabling adaptive navigation, particularly during transitions between locomotion modes. Strategies such as fluidic damping, variable-stiffness material, or phase-adaptive control could be explored to mitigate inertial effects and stabilize orientation during mode switching. In addition, developments in compact onboard power, miniature air compressors or pumps, pumpless phase-change materials or alternative actuation systems, and embedded control will be critical for improving untethered, adaptive, and autonomous operation. Furthermore, given the scale independency of monostability, scaling up the sizes of the present systems will facilitate the onboard integration. In parallel, hybrid deployment strategies may offer practical pathways for near-term applications. For example, an AUV or ROV serves as a mobile support station could transport multiple untethered soft swimmers to target locations and deploy them for localized exploration. This hierarchical approach would combine the endurance, navigation, and communication capabilities of conventional underwater vehicles with the compliance and maneuverability of soft swimmers, enabling distributed exploration. More broadly, these developments could enable distributed, cooperative soft robotic systems for long-duration environmental monitoring, inspection, and exploration in complex marine settings (*45*).

## MATERIALS AND METHODS

### Materials and fabrication of feather star–inspired soft robots

The feather star–inspired soft robots consisted of three primary components: a dual-channel pneumatic actuator, a monostable wing frame, and a stainless steel ballast. The pneumatic actuator was fabricated using standard molding and demolding techniques commonly used for soft pneumatic systems (fig. S2). Ecoflex 00-50 (Smooth-On) was selected as the elastomeric material for both the upper and lower actuator layers. After curing, the two layers were bonded using silicone rubber adhesive (Smooth-On) to form enclosed pneumatic channels. The wing frames were laser cut from 0.55-mm-thick polyester sheets (Grafix) and adhered to the actuator edges using super glue. The tips of each pair of parallel wing ribbons were bonded to create a prestressed multistable ribbon structure. To reduce structural bistability, the central sections of the buckled beams were trimmed, releasing stored strain energy. The trimmed frames were subsequently thermally treated at 52°C for several minutes to further relax residual stress and transition the structure from bistability to monostability. The completed wing frames were covered with Tegaderm transparent film (3M) to enhance surface durability and waterproofing. Two short plastic tubes were inserted into the actuator’s inlets and sealed with super glue and silicone adhesive to enable independent pneumatic control of the two channels. Last, a steel ball (0.68 g, ∼4.5% of the total robot weight of ∼14.5 g) was attached beneath the actuator’s center using super glue to slightly increase the robot’s density (>1 g/cm^3^), enabling stable underwater operation.

### Force-displacement measurement

The static mechanical responses of the multistable, bistable, and monostable star-shaped wing frames were characterized via quasistatic indentation tests using an Instron 5944 tensile tester. As illustrated in fig. S7, one end of the bottom wing ribbons was clamped, and the opposite wing tips were connected to a vertically aligned indenter. Tests were conducted under displacement control at a constant loading rate of 5 mm/min, with a normalized maximum displacement of 100% for each wing frame. The applied force and corresponding displacement were continuously recorded throughout the indentation cycle to generate force-displacement curves for precurved wing frames with varying arm lengths.

### Motion capture

The flapping kinematics of the feather star–inspired soft robots were recorded using high-speed cameras (Photron SA-2) operating at 500 frames/s. The recorded image sequences were analyzed using Photron FASTCAM Viewer (PFV4) software to extract flapping trajectories and angular displacements for each swimming mode.

### Aquatic experimental setup

Underwater swimming tests of the soft robots were conducted in two aquarium setups. A rectangular tank (122 cm by 52 cm by 32 cm) filled with 45 gallons (170.34 liters) of water was used for tests in jelly and fish modes, while a cubic tank (50 cm by 50 cm by 50 cm) containing 30 gallons (113.56 liters) of water was used for rotor mode tests.

An open-loop pneumatic control system was connected to the robots via a 3-m-long soft rubber tube. Actuation pressure *P*_snap_ of CH1 and CH2 in jelly mode for snapthrough is 40 kPa. Actuation pressure *P*_snap_ of CH1 or CH2 in fish and rotor modes for snapthrough is 45 kPa. Swimming motions were recorded using Photron SA-2 high-speed cameras, along with supplementary footage captured by Canon 6D Mark II and Sony a7 III cameras for both stationary and dynamic underwater testing.

### Analytical model of the buckled star-shaped wing

Cosserat rod model is used in the static equilibrium model of the structure. The governing equations for the movement and force equilibrium of a single beam are presented below. r denotes the rod frame origin position in the global coordinate system {A}. q represents the frame orientation in the quaternion form. F and M are the cross-sectional force and moment in the material frame {m}. f and m represent the external force and moment in {m}. η and ω are the frame translational velocity and angular velocity represented in {m}. s is the arc length parameter. RmA is the rotational matrix of frame {m} represented in the frame {A}. E and G are the young’s modulus and shear modulus. A is the cross-sectional area. w and t are the width and thickness of the beam cross section. Details can be found in (*46*)

dds[rqFM]=RmAKη−1F+η¯12L(q)[0(Kω−1M+ω¯)]+k1−qTqq−Kω−1M+ω¯×F−f−Kω−1M+ω¯×M−Kη−1F+η¯×F−m (1)

$$L\left(q\right)=\left[\begin{array}{cccc} q_{0} & -q_{1} & -q_{2} & -q_{3}\\ q_{1} & q_{0} & -q_{3} & q_{2}\\ q_{2} & q_{3} & q_{0} & -q_{1}\\ q_{3} & -q_{2} & q_{1} & q_{0} \end{array}\right],q=q_{0}+q_{1}i+q_{2}j+q_{3}k$$

$$K_{\eta }=\left[\begin{array}{ccc} \mathrm{GA} & 0 & 0\\ 0 & \mathrm{GA} & 0\\ 0 & 0 & \mathrm{EA} \end{array}\right],K_{\omega }=\left[\begin{array}{ccc} EI_{x} & 0 & 0\\ 0 & EI_{y} & 0\\ 0 & 0 & GI_{z} \end{array}\right]$$

$$\begin{array}{c} A=\mathrm{wt},I_{x}=\frac{wt^{3}}{12},I_{y}=\frac{tw^{3}}{12},I_{z}=\\ \frac{1}{3}wt^{3}\left\{1-\frac{192t}{\pi^{5}\;w}\sum_{n=1}^{\infty }\frac{1}{{\left(2n-1\right)}^{5}}\text{tanh}\left[\frac{\left(2n-1\right)\pi w}{2t}\right]\right\} \end{array}$$

For multibeam structure, the cross-sectional force and moment $Y_{s}$ at the starting point of each rod are unknown parameters. All rods are connected end to end, and therefore, starting from the first rod, the frame position on each rod and the ending point cross-sectional force $Y_{e}$ of all rods can be derived using Eq. 1.

At each connection point, the force equilibrium equations $Q\left(Y_{s},Y_{e}\right)=0$ need to be satisfied. For example, rods 2 and 3 should satisfy force equilibrium condition below. In addition, rods 1 and 12 are bonded together by satisfying the displacement conditions below. The interior-set method is used to solve these equations

−[F2eM2e]+[F3sM3s]=0

Ts1A=Te12A·Ry(−π4)

$F_{2}^{e}$ and $M_{2}^{e}$ are the cross-sectional force and moment at the ending point of rod 2. $F_{3}^{s}$ and $M_{3}^{s}$ are the cross-sectional force and moment at the starting point of rod 3. All forces in equilibrium equations are all represented in {A}. Ts1A is transformation matrix of starting point material frame of rod 1 represented in {A}. Te12A is transformation matrix of ending point material frame of rod 12 represented in {A}. $R_{y}\left(-\frac{\pi }{4}\right)$ represents rotating around y axis by $-\frac{\pi }{4}$.

### Flow measurement and simulation

3D flow fields were measured using multilayer stereoscopic PIV in a recirculating water channel (1.52 m by 0.38 m by 0.45 m). The soft robot was mounted at the channel center on streamlined supports and was operated in jelly and fish swimming modes at Reynolds numbers *O*(10^4^). The flow field was illuminated by opposing laser sheets and imaged by two high-speed cameras (Phantom, SpeedSense M341) with Scheimpflug adaptors. Phase-averaged velocity fields from sequential measurement layers were combined to reconstruct 3D, three-component flow fields for vortex visualization (*47*, *48*).

The CFD simulations were performed using an in-house direct numerical simulations solver (*49*). A computational model of the soft robot was created, and the kinematics were reconstructed from high-speed videos (movie S3) using a virtual scene setup. Flow conditions of the fish mode and the rotor mode were recreated in the solver, with the Reynolds number equal to 10,000. Cartesian mesh with tree-topological local mesh refinement (*49*) were used to reach a mesh density of Δ ∼ 0.003 BL. This mesh density was found appropriate for flow in the inertial regime, such as fish swimming (*50*). Subscale vortices were resolved using large eddy simulations (*51*, *52*).
