# Supplementary material for: Minimal-actuation feather star–inspired soft swimmers for multimodal 3D maneuverability
Source: Sci Adv. 2026 Oct 7;12(41):eaeg9211. doi: 10.1126/sciadv.aeg9211 (PMC13644798; doi:10.1126/sciadv.aeg9211)
Supplement: Supplementary file 1 [file sciadv.aeg9211_sm.pdf]

Science Advances
AAAS

Supplementary Materials for

Minimal-actuation feather star–inspired soft swimmers for multimodal 3D maneuverability

Haitao Qing *et al.*

Corresponding author: Jie Yin, jyin8@ncsu.edu

Sci. Adv. **12**, eaeg9211 (2026)
DOI: 10.1126/sciadv.aeg9211

The PDF file includes:

Supplementary Text
Figs. S1 to S37
Tables S1 to S3
Legends for movies S1 to S14

Other Supplementary Material for this manuscript includes the following:

Movies S1 to S14

Supplementary Text

Analytical model of the buckled star-shaped wing

Each beam of the star-shaped wing is modeled as a geometrically exact Cosserat rod. Details can be found in reference *(46)*. The global frame is $\{A\} = (x_A, y_A, z_A)$. The material frame attached to each cross-section is $\{m\} = (\boldsymbol{e}_1, \boldsymbol{e}_2, \boldsymbol{e}_3)$, with $\boldsymbol{e}_3$ aligned with the centerline tangent in the reference configuration. The arc-length coordinate along beam i is $s \in [0, L_i]$. The rod centerline and orientation are described by

$$\boldsymbol{r}_i(s) \in \mathbb{R}^3, \boldsymbol{q}_i(s) \in \mathbb{R}^4,$$

Where q_i represents unit quaternion mapping the material frame $\{m_i\}$ to the global coordinate system $\{A\}$. The rotation matrix associated with $\boldsymbol{q}_i = [q_0, q_1, q_2, q_3]^T$ is

$$^A\boldsymbol{R}_i(\boldsymbol{q}_i) = \begin{bmatrix} q_0^2 + q_1^2 - q_2^2 - q_3^2 & 2(q_1q_2 - q_0q_3) & 2(q_1q_3 + q_0q_2) \\ 2(q_1q_2 + q_0q_3) & q_0^2 - q_1^2 + q_2^2 - q_3^2 & 2(q_2q_3 - q_0q_1) \\ 2(q_1q_3 - q_0q_2) & 2(q_2q_3 + q_0q_1) & q_0^2 - q_1^2 - q_2^2 + q_3^2 \end{bmatrix}. \tag{1}$$

The internal force and moment in the material frame are

$$\boldsymbol{F}_i(s) = [F_{ix}, F_{iy}, F_{iz}],$$

$$\boldsymbol{M}_i(s) = [M_{ix}, M_{iy}, M_{iz}].$$

External distributed force and moment per unit length are $\boldsymbol{f}_i(s)$ and $\boldsymbol{m}_i(s)$.

In the geometrically exact Cosserat formulation, we define the shear/extension and curvature/twist strains:

$$\boldsymbol{v}_i = \boldsymbol{K}_\eta^{-1}\boldsymbol{F}_i + \boldsymbol{\eta}_i, \boldsymbol{\Omega}_i = \boldsymbol{K}_\omega^{-1}\boldsymbol{M}_i + \overline{\boldsymbol{\omega}}_i. \tag{2}$$

Here: $\boldsymbol{v}_i = [v_{ix}, v_{iy}, v_{iz}]^T$ is the generalized shear/extension strain, $\boldsymbol{\Omega}_i = [k_{ix}, k_{iy}, k_{iz}]^T$ is the generalized curvature/twist strain, $\boldsymbol{\eta}_i$ and $\overline{\boldsymbol{\omega}}_i$ can be used to introduce pre-strain or other reference effects, $\boldsymbol{K}_\eta$ and $\boldsymbol{K}_\omega$ are the shear/extension and bending/torsion stiffness matrices.

For a rectangular cross-section of width w and thickness t,

$$A = wt, I_x = \frac{wt^3}{12}, I_y = \frac{tw^3}{12}, I_z = \frac{1}{3}wt^3\left(1 - \frac{192t}{\pi^5 w}\sum_{n=1}^{\infty}\frac{1}{(2n-1)^5}\tanh(\frac{(2n-1)\pi w}{2t})\right) \quad (3)$$

and

$$\boldsymbol{K}_\eta = \begin{bmatrix} GA & 0 & 0 \\ 0 & GA & 0 \\ 0 & 0 & EA \end{bmatrix}, \boldsymbol{K}_\omega = \begin{bmatrix} EI_x & 0 & 0 \\ 0 & EI_y & 0 \\ 0 & 0 & GI_z \end{bmatrix}. \quad (4)$$

The centerline tangent in the global frame is the rotated material strain vector $\boldsymbol{v}_i$:

$$\frac{d\boldsymbol{r}_i}{ds} = {}^A\boldsymbol{R}_i(\boldsymbol{q}_i)\boldsymbol{v}_i = {}^A\boldsymbol{R}_i(\boldsymbol{q}_i)(\boldsymbol{K}_\eta^{-1}\boldsymbol{F}_i + \overline{\boldsymbol{\eta}}_i). \quad (5)$$

Component-wise, if

$${}^A\boldsymbol{R}_i = \left[R_{ij}\right], \qquad j = 1, 2, 3,$$

then

$$\frac{dr_{ix}}{ds} = R_{11}v_{ix} + R_{12}v_{iy} + R_{13}v_{iz},$$

$$\frac{dr_{iy}}{ds} = R_{21}v_{ix} + R_{22}v_{iy} + R_{23}v_{iz},$$

$$\frac{dr_{iz}}{ds} = R_{31}v_{ix} + R_{32}v_{iy} + R_{33}v_{iz}. \quad (6)$$

The quaternion evolution is governed by

$$\frac{d\boldsymbol{q}_i}{ds} = \frac{1}{2}\boldsymbol{L}(\boldsymbol{q}_i)\boldsymbol{\Omega}_i + k(1 - \boldsymbol{q}_i^T\boldsymbol{q}_i)\boldsymbol{q}_i, \quad (7)$$

where k is a penalty parameter enforcing the unit-norm constraint on $\boldsymbol{q}_i$, and $\boldsymbol{L}(\boldsymbol{q}_i)$ is the quaternion multiplication matrix:

$$\boldsymbol{L}(\boldsymbol{q}_i) = \begin{bmatrix} q_0 & -q_1 & -q_2 & -q_3 \\ q_1 & q_0 & -q_3 & q_2 \\ q_2 & q_3 & q_0 & -q_1 \\ q_3 & -q_2 & q_1 & q_0 \end{bmatrix}. \quad (8)$$

Using $\boldsymbol{\Omega}_i = [k_{ix}, k_{iy}, k_{iz}]^T$, Eq. (8) can be written component-wise as

$$\frac{dq_0}{ds} = \frac{1}{2}\left(-q_1 k_{ix} - q_2 k_{iy} - q_3 k_{iz}\right) + k(1 - q_0^2 - q_1^2 - q_2^2 - q_3^2)q_0,$$

$$\frac{dq_1}{ds} = \frac{1}{2}\left(q_0 k_{ix} - q_3 k_{iy} + q_2 k_{iz}\right) + k(1 - q^T q)q_1,$$

$$\frac{dq_2}{ds} = \frac{1}{2}\left(q_3 k_{ix} + q_0 k_{iy} - q_1 k_{iz}\right) + k(1 - q^T q)q_2,$$

$$\frac{dq_3}{ds} = \frac{1}{2}\left(-q_2 k_{ix} + q_1 k_{iy} + q_0 k_{iz}\right) + k(1 - q^T q)q_3.$$

(9)

In the material frame, static balance of linear momentum yields

$$\frac{d\boldsymbol{F}_i}{ds} + \boldsymbol{\Omega}_i \times \boldsymbol{F}_i + \boldsymbol{f}_i = 0. \quad (10)$$

Expanding in components:

$$\frac{dF_{ix}}{ds} + \left(k_{iy}F_{iz} - k_{iz}F_{iy}\right) + f_{ix} = 0,$$

$$\frac{dF_{iy}}{ds} + (k_{iz}F_{ix} - k_{ix}F_{iz}) + f_{iy} = 0,$$

$$\frac{dF_{iz}}{ds} + \left(k_{ix}F_{iy} - k_{iy}F_{ix}\right) + f_{iz} = 0.$$

Static balance of angular momentum in the material frame gives

$$\frac{d\boldsymbol{M}_i}{ds} + \boldsymbol{\Omega}_i \times \boldsymbol{M}_i + \boldsymbol{v}_i \times \boldsymbol{F}_i + \boldsymbol{m}_i = 0. \quad (11)$$

Expanding in components:

$$\frac{dM_{ix}}{ds} + \left(k_{iy}M_{iz} - k_{iz}M_{iy}\right) + \left(v_{iy}F_{iz} - v_{iz}F_{iy}\right) + m_{ix} = 0,$$

$$\frac{dM_{iy}}{ds} + (k_{iz}M_{ix} - k_{ix}M_{iz}) + (v_{iz}F_{ix} - v_{ix}F_{iz}) + m_{iy} = 0,$$

$$\frac{dM_{iz}}{ds} + (k_{ix}M_{iy} - k_{iy}M_{ix}) + \left(v_{ix}F_{iy} - v_{iy}F_{ix}\right) + m_{iz} = 0.$$

The wing-like ribbon frame consists of $N = 12$ beams connected in a star topology. For each beam i: $s = 0$ is its starting cross-section, with state $\boldsymbol{Y}_i(0) = \boldsymbol{Y}_i^s$; $s = L_i$ is its ending cross-section, with state $\boldsymbol{Y}_i(L_i) = \boldsymbol{Y}_i^e$. Integrating the single-rod ODEs gives a nonlinear mapping

$$\boldsymbol{Y}_i^e = \boldsymbol{\Phi}_i(\boldsymbol{Y}_i^s), \tag{12}$$

where $\boldsymbol{\Phi}_i$ is obtained using a shooting method.

At each connection node between beam i and beam j, we enforce positional compatibility

$$\mathbf{r}_i^e = \mathbf{r}_j^s. \tag{13}$$

and rotational compatibility

$${}^A\boldsymbol{R}_i^e = {}^A\boldsymbol{R}_j^s\boldsymbol{R}_{ij}, \tag{14}$$

where $\boldsymbol{R}_{ij}$ is a simple rotation such as $R_y(\frac{\pi}{4})$.

Force equilibrium:

$$\mathbf{F}_i^e + \mathbf{F}_j^s = \mathbf{0}. \tag{15}$$

Moment equilibrium:

$$\mathbf{M}_i^e + \mathbf{M}_j^s + (\mathbf{r}_i^e - \mathbf{r}_{\text{node}}) \times \mathbf{F}_i^e + (\mathbf{r}_j^s - \mathbf{r}_{\text{node}}) \times \mathbf{F}_j^s = \mathbf{0}, \tag{16}$$

where $\mathbf{r}_{\text{node}}$ is the common node position. When the node is taken at $\mathbf{r}_i^e = \mathbf{r}_j^s$, the last two cross terms cancel, and Eq. (16) simplifies to $\mathbf{M}_i^e + \mathbf{M}_j^s = 0$.

For the specific example in the text, rods 2 and 3 share a node and satisfy

$$-\begin{bmatrix}\boldsymbol{F}_2^e\\ \boldsymbol{M}_2^e\end{bmatrix} + \begin{bmatrix}\boldsymbol{F}_3^s\\ \boldsymbol{M}_3^s\end{bmatrix} = 0, \tag{17}$$

after transforming all quantities into the same frame. $\boldsymbol{F}_2^e$ and $\boldsymbol{M}_2^e$ is the cross-section force and moment at the ending point of rod 2. $\boldsymbol{F}_3^s$ and $\boldsymbol{M}_3^s$ is the cross-section force and moment at the starting point of rod 3. All forces in equilibrium equations are all represented in $\{A\}$.

To close the 12-beam ring, the end of beam 12 and the start of beam 1 must coincide, with a prescribed relative rotation

$$\mathbf{r}_{12}^{e} = \mathbf{r}_{1}^{s}, \tag{18}$$

$$^{A}\boldsymbol{R}_{12}^{e}\boldsymbol{R}_{y}\left(-\frac{\pi}{4}\right) = {}^{A}\boldsymbol{R}_{1}^{s}, \tag{19}$$

in addition, Rod 1 and Rod 12 are bonded together by satisfying the displacement conditions below. The interior-set method is used to solve these equations

$$^{A}_{1}\boldsymbol{T}_{s} = {}^{A}_{12}\boldsymbol{T}_{e} \cdot \boldsymbol{R}_{y}(-\frac{\pi}{4}), \tag{20}$$

$^{A}_{1}\boldsymbol{T}_{s}$ is transformation matrix of starting point material frame of rod 1 represented in $\{A\}$. $^{A}_{12}\boldsymbol{T}_{e}$ is transformation matrix of ending point material frame of rod 12 represented in $\{A\}$. $\boldsymbol{R}_{y}\left(-\frac{\pi}{4}\right)$ represents rotating around y axis by $-\frac{\pi}{4}$.

Collect all unknown starting states of the N=12 beams in a global vector

$$\mathbf{Y}_{s} = [(\mathbf{y}_{1}^{s})^{T}, (\mathbf{y}_{2}^{s})^{T}, \ldots, (\mathbf{y}_{12}^{s})^{T}]^{T}. \tag{21}$$

Using Eq. (12), we compute the corresponding beam end states

$$\mathbf{Y}_{e} = \Phi(\mathbf{Y}_{s}) = [(\mathbf{y}_{1}^{e})^{T}, \ldots, (\mathbf{y}_{12}^{e})^{T}]^{T}. \tag{22}$$

and assemble all connection, closure, and boundary conditions into a residual vector

$$\boldsymbol{Q}(\boldsymbol{Y}_{s}, \boldsymbol{Y}_{e}) = 0. \tag{23}$$

Because $\boldsymbol{Y}_{e}$ is a nonlinear function of $\boldsymbol{Y}_{s}$, we obtain a closed nonlinear algebraic system

$$\mathbf{R}(\mathbf{Y}_{s}) = \mathbf{Q}(\mathbf{Y}_{s}, \Phi(\mathbf{Y}_{s})) = 0. \tag{24}$$

At convergence, $\boldsymbol{Y}_{s}$, $\boldsymbol{Y}_{e}$ define the complete 3-D buckled configuration and the internal force/moment distribution of the star-shaped wing frame.

3D Scanning

3D scanned and reconstruction models of the feather starfish-inspired soft robot are obtained using blue laser 3D scanning technique. A Creality CR-Scan Raptor 3D Scanner (fig. S15) is used to precisely capture and reconstruct the 3D models. To improve 3D scan quality, soft robots are fully coated with REVOPOINT ABP 3D scanning spray before scanning. The scanned models are then post-processed in CrealityScan software. Scanned images of the soft robot (AL = 45 mm) are shown in fig. S15. The analytical model closely agrees with the 3D-scanned model of the soft robot (AL = 45 mm), as shown in Fig. S15, with minor discrepancies attributed to the adhesion of the pneumatic soft actuator and wing frame.

Setup for the particle image velocimetry tests

To measure the three-dimensional flow field around the soft robot, we performed multi-layer stereoscopic particle image velocimetry (SPIV) experiments in a recirculating water channel. The schematic of the test section (length × width × height = 1.52 m × 0.38 m × 0.45 m) is shown in Fig. S25A**.** In both the jelly and fish modes, the robot was suspended at the channel center using 3D-printed supports with aerodynamic fairings. For the jelly mode (figs. S20B−C), the robot was oriented perpendicular to the free-stream flow. The flow velocity was set to 0.2 m/s, corresponding to a chord-based Reynolds number of Re = ρUc/μ = 20,000, where c = 0.1 m is the robot width, and ρ and μ are the water density and viscosity, respectively. The robot was actuated at f = 2.5 Hz, yielding a Strouhal number of St = *f*A/U = 1.25, where A = c represents the wake width. For the fish mode (figs. S20E−F), the robot was aligned with the flow, with an upstream forearm, a downstream rear arm, and two side arms. The characteristic length scale in this mode is the diagonal width of the robot, c = 0.141 m. The flow velocity was set to 0.1 m/s, and the robot was actuated at 2.5 Hz with a rear-arm amplitude of A = 0.06 m (see also Fig. 2H), resulting in Re = 14,100 and St = 1.05.

In the SPIV experiments, the flow was seeded with neutrally buoyant silver-coated hollow ceramic particles (50 μm diameter). The particles were illuminated by two horizontal laser sheets generated by continuous-wave lasers (532 nm, 5 W Raypower MGL-W-532 and 10 W CNI MGL-W-532A) with sheet optics, directed from opposite sides to minimize shadowing. Two high-speed cameras (Phantom SpeedSense M341, 2560 × 1600 pixels) equipped with Scheimpflug adaptors were used to image the particles. After calibration, the images were processed in Dantec Dynamic Studio

(v6.9) to obtain two-dimensional three-component (2D3C) velocity fields using an adaptive PIV algorithm with interrogation window sizes ranging from 32 × 32 to 64 × 64 pixels.

Velocity components in the *x*-, *y*-, and *z*-directions were obtained for each measurement layer. The robot was then translated vertically by 5 mm using a vertical traverse to acquire the next layer. This procedure was repeated until the upper half of the robot and additional buffer layers were covered. In total, 17 layers were measured for jelly mode and 19 layers for fish mode. Assuming symmetry about the mid-plane, the measured layers were mirrored to yield 33 layers for jelly mode and 37 layers for fish mode. For each layer, 750 consecutive image pairs were recorded from each camera at 61.5 Hz. The resulting velocity fields were phase-binned into 30 bins per actuation cycle, yielding approximately 25 realizations per bin. The velocity fields within each bin were phase-averaged to suppress small-scale, cycle-to-cycle variations while preserving coherent flow structures across cycles and layers. The phase-averaged multi-layer 2D3C velocity fields were then combined to reconstruct three-dimensional three-component (3D3C) velocity fields.

The reconstructed flow domain spanned 0.33 m × 0.21 m × 0.16 m for the jelly mode and 0.33 m × 0.21 m × 0.18 m for the fish mode. These 3D velocity fields were used to visualize the flow structures. Similar multi-layer SPIV approaches have been used to resolve three-dimensional flow structures generated by propulsors and airfoils (*47*, *48*). This technique requires high cycle-to-cycle repeatability for our soft robot, which is demonstrated by the coherent structures obtained in the present study. To identify vortical structures, we computed the second invariant of the velocity gradient tensor, $Q = 0.5(||\mathbf{\Omega}||^2 - ||\mathbf{S}||^2)$, where $\mathbf{\Omega}$ is the vorticity tensor and $\mathbf{S}$ is the strain-rate tensor. Vortical structures were visualized using iso-Q surfaces colored by the spanwise vorticity, $\omega = dv/dx - du/dy$, where u and v denote the velocity components in the *x*- and *y*-directions, respectively. Sample iso-Q surface plots are shown in fig. S20D−G**.** For the jelly mode, iso-surfaces of $Q = 10\ s^{-2}$ were plotted, with the surfaces colored by spanwise vorticity over a range of $\omega = \pm 15\ s^{-1}$. For the fish mode, iso-surfaces of $Q = 2\ s^{-2}$ were plotted, with a corresponding vorticity range of $\omega = \pm 10\ s^{-1}$.

Flow and vortex dynamics of Jelly Mode propulsion

In fig. S21, we detail our analysis of the flow and vortex dynamics associated with jelly mode propulsion. The top panel of fig. S21 shows a time-lapse of the pressure signal replotted from Fig. 3A. We selected five representative time instances within the cycle for analysis. At time instance

(i), which corresponds to the onset of the actuation stroke, the fins begin to move downward. At this stage, a pair of strong stopping vortices generated during the previous cycle remains directly beneath the fins. As the actuation proceeds to time instance (ii), downward fin motion leads to the formation of a new pair of starting vortices on the upper surfaces of the fins. Meanwhile, the stopping vortices from the previous cycle remain adjacent to the fins. The interaction between these counter-rotating starting and stopping vortices creates an effective virtual wall between them, enhancing pressure on the lower fin surfaces and contributing to upward propulsion, consistent with wall effect mechanisms in jellyfish swimming. By the end of the actuation stroke at time instance (iii), the starting vortices have grown in strength, and the virtual wall effect persists throughout the stroke, continuing to augment thrust generation.

As the fins transition into the relaxation stroke at time instance (iv), they move back upward and generate a new pair of stopping vortices beneath the fins, while the previously formed starting vortices are shed from the fin edges. During the remainder of the relaxation stroke (time instance v), both the starting vortices and the older stopping vortices are convected downstream, leaving only the newly formed stopping vortices near the fins. These new stopping vortices subsequently contribute to passive energy recapture by inducing flow toward the lower fin surfaces that decelerates against the fins, generating elevated pressure during relaxation and allowing continued forward motion without additional actuation. The cycle then repeats. Overall, the sequence of vortex generation, interaction, and shedding of our robot closely mirrors the canonical vortex dynamics observed in jellyfish propulsion, despite differences in bell geometry.

Setup for the computational fluid dynamics

To visualize the interaction between the actuating robot wings and the surrounding flow in varying modes, computational fluid dynamics (CFD) was conducted using kinematics reconstructed from high-speed videos (movie S3). First, a computational model was created and meshed using Autodesk Maya. The main body containing the pneumatic channels was modeled as a volumetric solid, and each of the four wings was modeled as a separate membranous surface, as shown in fig. S22A. To reconstruct the swimmer's kinematics across different modes, a virtual scene is set up as shown in Fig. S27B, following similar procedures used to reconstruct insect *(49)*, bird *(50)*, and fish locomotion *(20)*. High-speed videos (movie S3) taken from different angles of a cycle of

actuation are projected onto image planes at corresponding positions around the computational model. Combining previous methods of joint-based and point-based reconstruction, we used a cross-shaped virtual skeleton to match the body deformation with that of the video, and moved control points mapped onto wings to match the wing deformation. After the reconstruction of kinematics was complete, we remeshed the computational model using a Catmull-Clark subdivision method to achieve the resolution of surface triangular meshes shown in fig. S22A.

The numerical method for the flow simulation combines the direct numerical simulation (DNS) and the immersed boundary method (IBM). The governing equations are the incompressible Navier-Stokes equations.

$$\nabla \cdot \vec{u} = 0;$$

$$\frac{\partial \vec{u}}{\partial t} + \vec{u} \cdot \nabla \vec{u} = -\nabla p + \frac{1}{Re} \nabla^2 \vec{u}$$

The CFD algorithm used here is detailed by Zhang et al. *(48)*, including the implementation of the tree-topological local mesh refinement (TLMR) to refine regions of interest locally, the treatment of internal and external boundary conditions, and various validations.

The computational domains for the fish and rotor modes are shown in figs. S22C−D. Both domains share the same dimensions of (10, 10, 10) BL, but the discretization differs due to the orientation of the flow. With the computational model oriented upright, to simulate the fish and rotation modes while ascending, the incoming flow is imposed from the left and above, respectively. Flow speeds are chosen to match the Strouhal numbers experienced in the experiments. The Strouhal numbers based on the wake width for the fish and rotor modes are 1.23 and 0.92. For the fish mode, the flapping amplitude is chosen as the wake width, and for the rotor mode, the wake width is the chord length at the wing base. The Reynolds number for both cases is set to be 10000.

The domain is discretized using Cartesian grids, as shown in figs. S22B−C. In this schematic, the grid count is reduced by half compared to the nominal mesh for the sake of visibility. Plenty of stretch nodes are used to fully develop the incoming flow before it reaches the computational model enclosed in the dense region. Downstream of the dense region, high-density stretched nodes with limited stretching were used to ensure that the perturbed wakes safely advect out of the flow domain. To further enhance the resolution around the model and its near-wake, TLMR blocks were placed within the dense region to further subdivide the background mesh. The layout of the nested and interlocking TLMR blocks are shown in figs. S22E−F. Each TLMR level (blue and orange)

subdivides the mesh of the previous level by a factor of 2 in any dimension. The innermost TLMR level is composed of three blocks fully enclosing the model at any point during its cyclic motion. The setup of these blocks evenly distributes mesh counts. The minimum mesh dimensions are (2.87, 3.25, 3.25) × 10^{-3} BL in the streamwise, lateral, and transverse directions. The streamwise directions for the fish and rotor modes are along the *x*- and *z*-axes, respectively. These mesh settings have been consistently found to reveal flow behaviors in the inertial regime accurately (Re $\in (10^3, 10^5)$) *(51, 52)*. Large-eddy simulation (LES) with a dynamic Lagrangian method has been used to resolve subscale small vortices.

Hydrodynamic force production in Fish Mode

The instantaneous coefficients of thrust (C_T) and lift (C_L) of the fins and the body of the robot over a whole snapping cycle are shown in fig. S23. Overall, the robot produces balanced net forces in the streamwise and vertical directions. The main body and the upstream-pointing fin produce mostly drag ($C_T < 0$), while the side and back fins produce varying amounts of thrust and drag. The main propulsor responsible for most of the thrust can be identified as the back fin (red line in fig. S23), and the side fins produce a moderate amount of thrust. In both the snap-through and snap-back phases, there is a similar trend of sustained positive thrust followed by a sharp dip into negative thrust. Positive thrust is produced by the rapid backward acceleration of the added fluid mass, and as the fins approach the terminal points of their flapping motion, the forces are more oriented in the vertical direction. Notably, at t/T = 0.4, the sharp loss of thrust coincides with the rapid increase of lift (fig. S23). This high lift production in static flapping is responsible for the upward motion of the robot when free swimming in the fish mode, as shown in Fig. 3H.

Hydrodynamics moment production in Rotor Mode

The instantaneous coefficient of hydrodynamic yaw moment of the rotor mode is shown in fig. S25A. This corresponds to the CFD simulation shown in Fig. 4F, with the motion reconstructed from the tethered experiments (movie S3). The snapping fins are responsible for the net negative C_M over a cycle, which would result in a clockwise rotation when free swimming. Though larger amplitudes of C_M are generated by the side fins due to the high effective angle of attack of their chords, their simultaneous actions result in nearly equal and opposite C_M which cancel each other

out. Ultimately, the non-zero C_M produced by the snapping fins due to their spanwise twist, though smaller in amplitude compared to the companion fins, is the main contributor to the nonzero cycle-average C_M.

Fabrication of soft magnets

The soft magnetic composite was fabrication by blending uncured Ecoflex 00-50 silicone elastomer (Smooth-On) with neodymium–iron–boron (NdFeB) magnetic microparticles (MQFP-B+20174-088, Magnequench). Part A and Part B of Ecoflex 00-50 were mixed in a 1:1 weight ratio, followed by the addition of NdFeB particles at a concentration of 60 wt%. The mixture was thoroughly stirred and degassed under vacuum for 5 minutes to eliminate entrapped air. The composite was cast into a cylindrical mold (radius: 10 mm; height: 3.5 mm) fabricated using a Mars 5 Ultra 3D printer (Elegoo Inc.). After curing, the cylindrical samples were axially magnetized by placing them between the poles of a GMW 3472-70 electromagnet, applying a uniform magnetic field of 1.75 T for 3 minutes to align and magnetize the embedded particles. Following magnetization, the residual surface magnetism of the soft magnet was measured using a magneto-optical sensor (CMOS-MagView, Matesy, Jena, Germany) and found to be 26.8 mT in the direction perpendicular to the axial axis (See details in fig. S28).

Evaluation criteria of hexagon radar charts

The hexagon radar charts are constructed based on six key evaluation criteria that assess the overall performance of bioinspired soft swimming robots (figs. S34-S37 and Table S1-S3). These criteria include:

1. Swimming speed

   The maximum relative swimming speed of each soft robot (measured in BL/s) is normalized by the highest value within the respective category.
2. Rotation speed

   The maximum rotation speed (in °/s) of each robot is normalized by the highest value within its category.

3. Lightweight

   If the weight of soft swimming robot is larger than 1 kg, it will get 0 point; if the weight of soft swimming robot is less than or equal to 1 kg but larger than 0.5 kg, it will get 0.5 points; and if the weight of soft swimming robot is less than or equal to 0.5 kg, it will get 1 point.

4. Maneuverability

   3D maneuverability will get 1 point, 2D will get 0.75 points, 2D (surface only) will get 0.5 points, 1D will get 0.25 points, and 1D (surface only) will get 0.125 points.

5. Normalized Cost of Transport ($\overline{CoT}$)

   The normalized CoT of each soft robot is normalized by the highest value within the respective category.

6. Functionality

   If the soft swimmer has practical functionalities except swimming capability, it will get 1 point; if the soft swimmer only focuses on the swimming capability, it will get 0.5 points.

Acronyms in the tables: Tethered (TT); Untethered (UTT); Jelly Mode (J); Fish Mode (F); Rotor Mode (R); Body length (BL); Dielectric elastomer actuator (DEA); Shape memory alloy (SMA); Hydraulically amplified self-healing electrostatic actuator (HASEL).

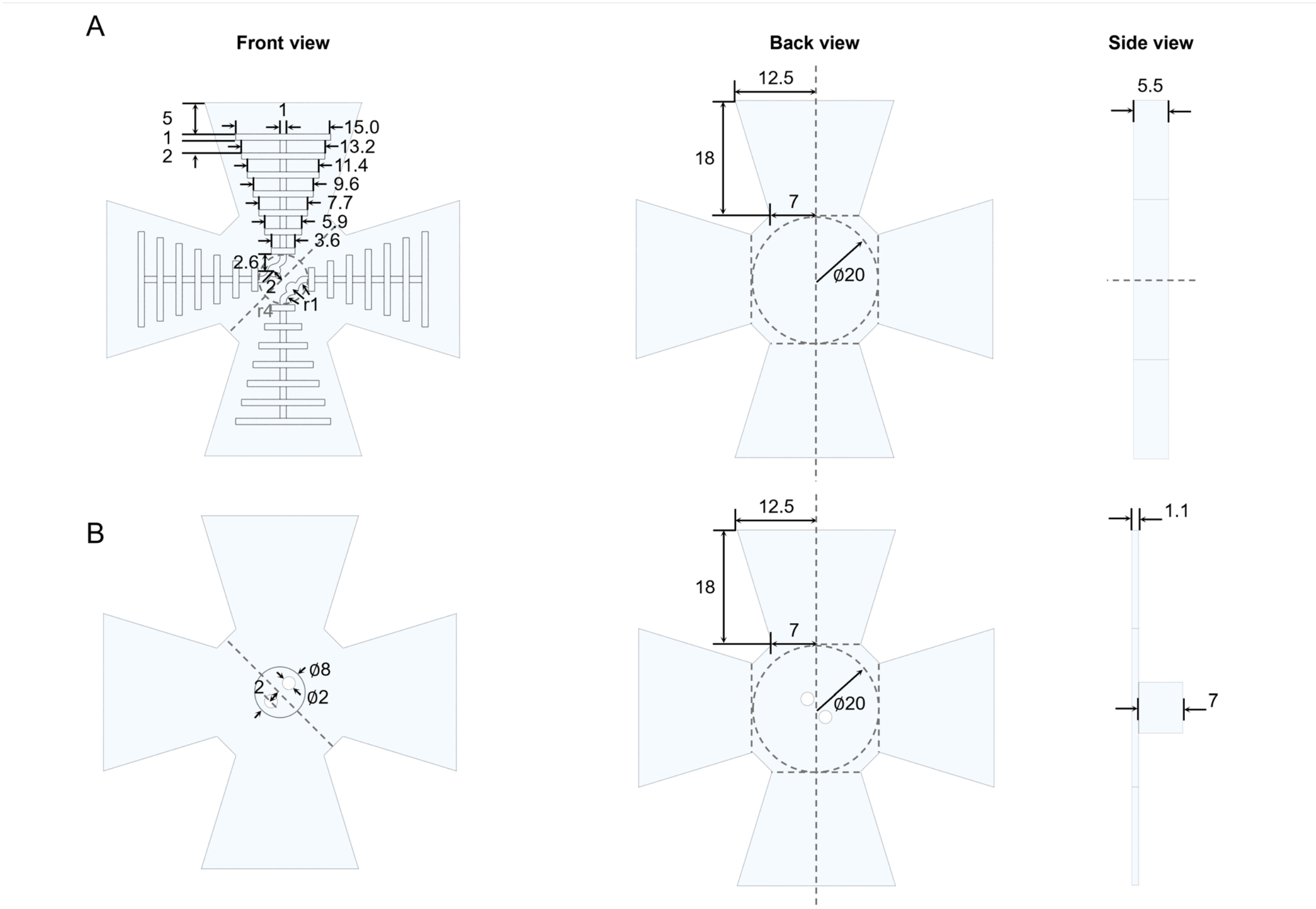

Figure S1. Geometries of the octagonal dual-channel soft bending actuator. (A) Bottom elastomeric layer. (B) Top elastomeric layer.

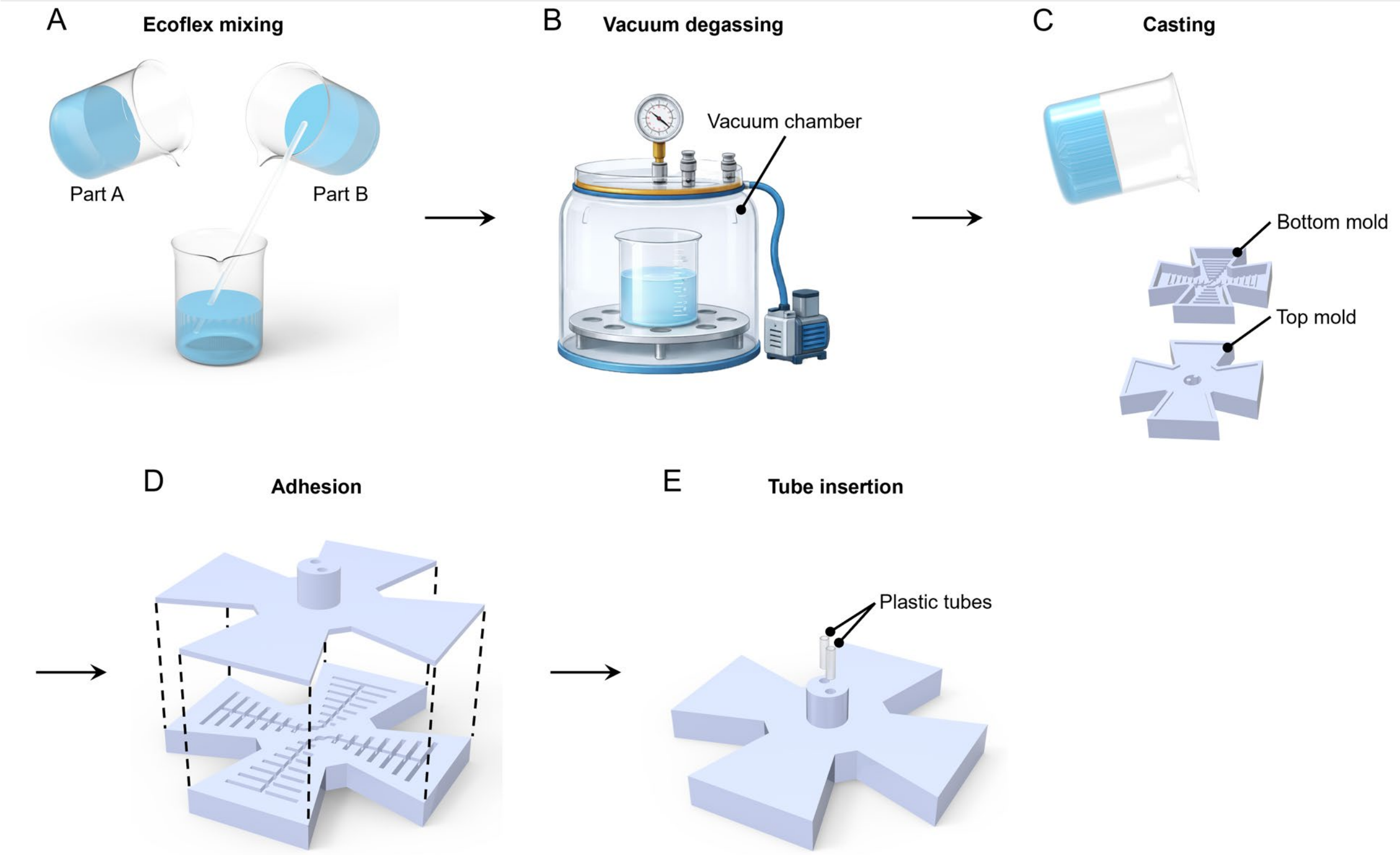

Figure S2. Fabrication procedure of the dual-channel soft bending actuator. (A) Mixing Ecoflex Part A and Part B to form a uniform elastomer precursor. (B) Vacuum degassing to remove trapped air bubbles from the Ecoflex mixture. (C) Casting and curing of the actuator by pouring Ecoflex into two molds. (D) Assembly of the elastomeric soft actuator using a silicone rubber adhesive. (E) Insertion and sealing of plastic tubes into the air inlets of the actuator.

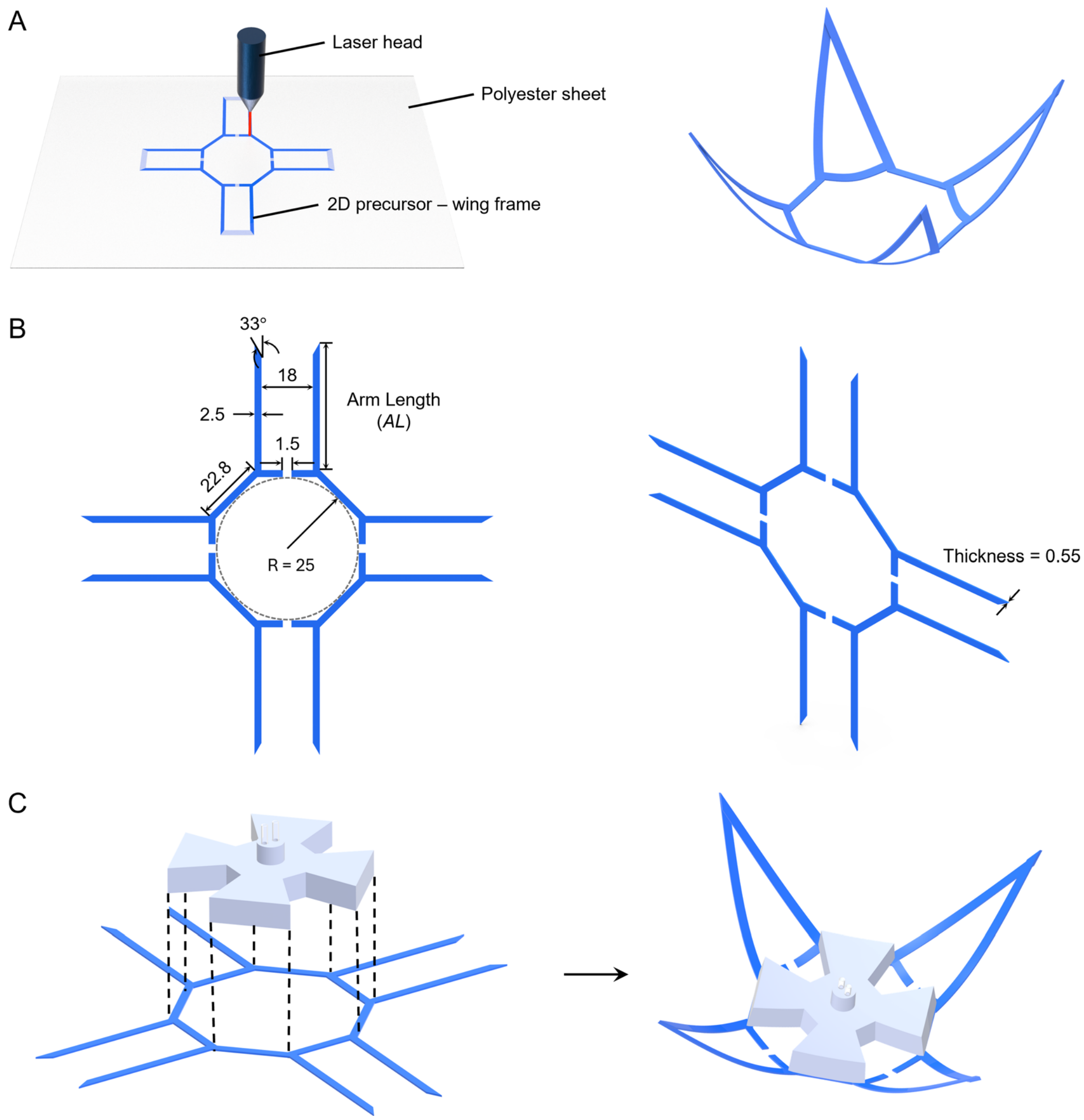

Figure S3. Geometry and fabrication procedure of wing-like ribbon frame. (A) Laser cutting of polyester ribbon frame. Bonding four pairs of tips of parallel ribbons to form the multistable ribbon frame. (B) Geometries of wing-like ribbon frames. (C) Bonding soft actuator with ribbon frame.

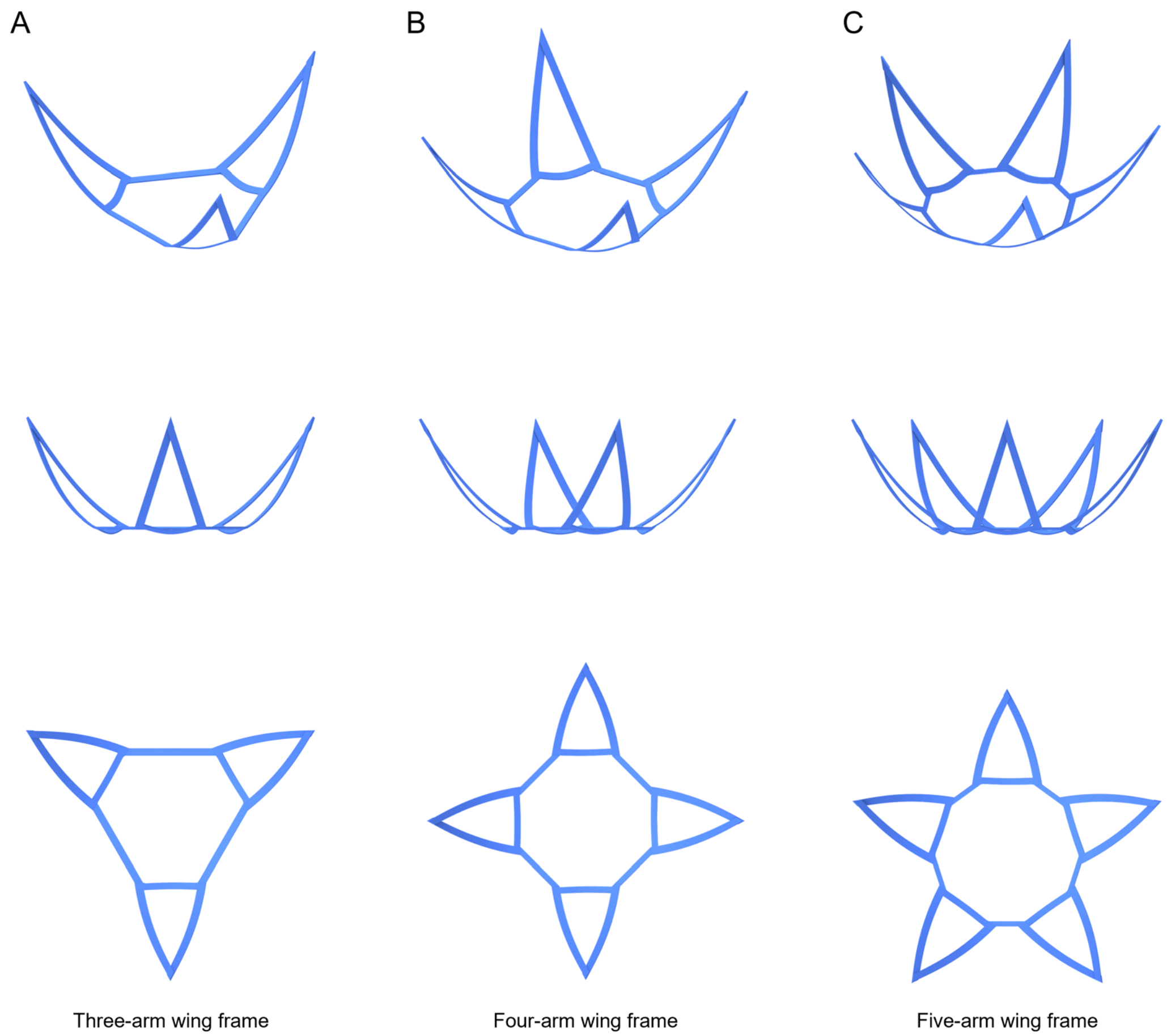

Figure S4. Schematics of three-, four-, and five-arm wing-like ribbon frame.

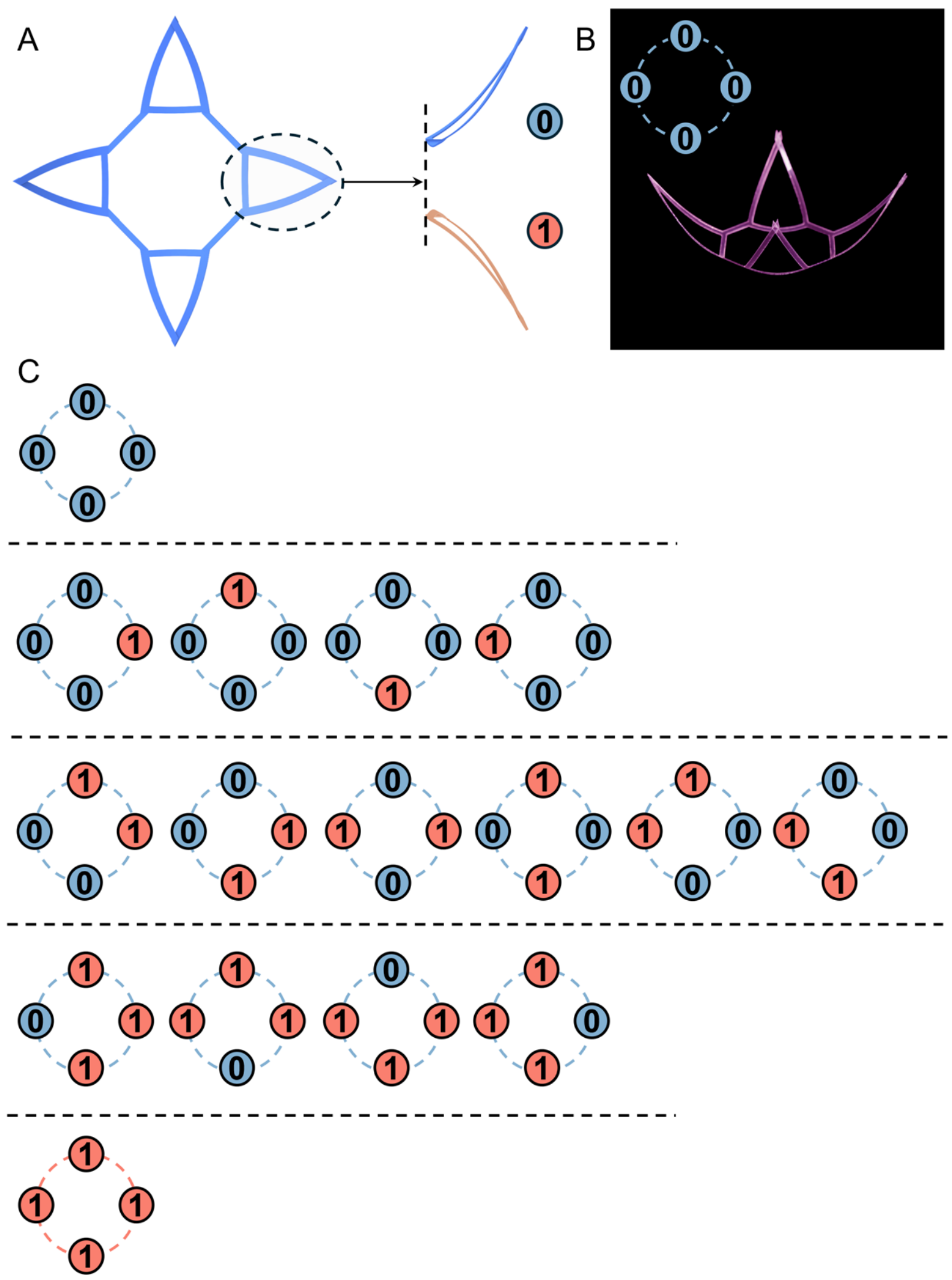

Figure S5. Configuration and stable states of four-arm ribbon frame. (A) Schematic of the four-arm ribbon frame, consisting of four bistable buckled ribbons connected by an octagonal ribbon frame. (B) Definition of the bistable states of an individual buckled ribbon: the upper configuration corresponds to the "0" state, whereas the lower configuration corresponds to the "1" state. (C) All 16 possible stable states of the four-arm ribbon frame.

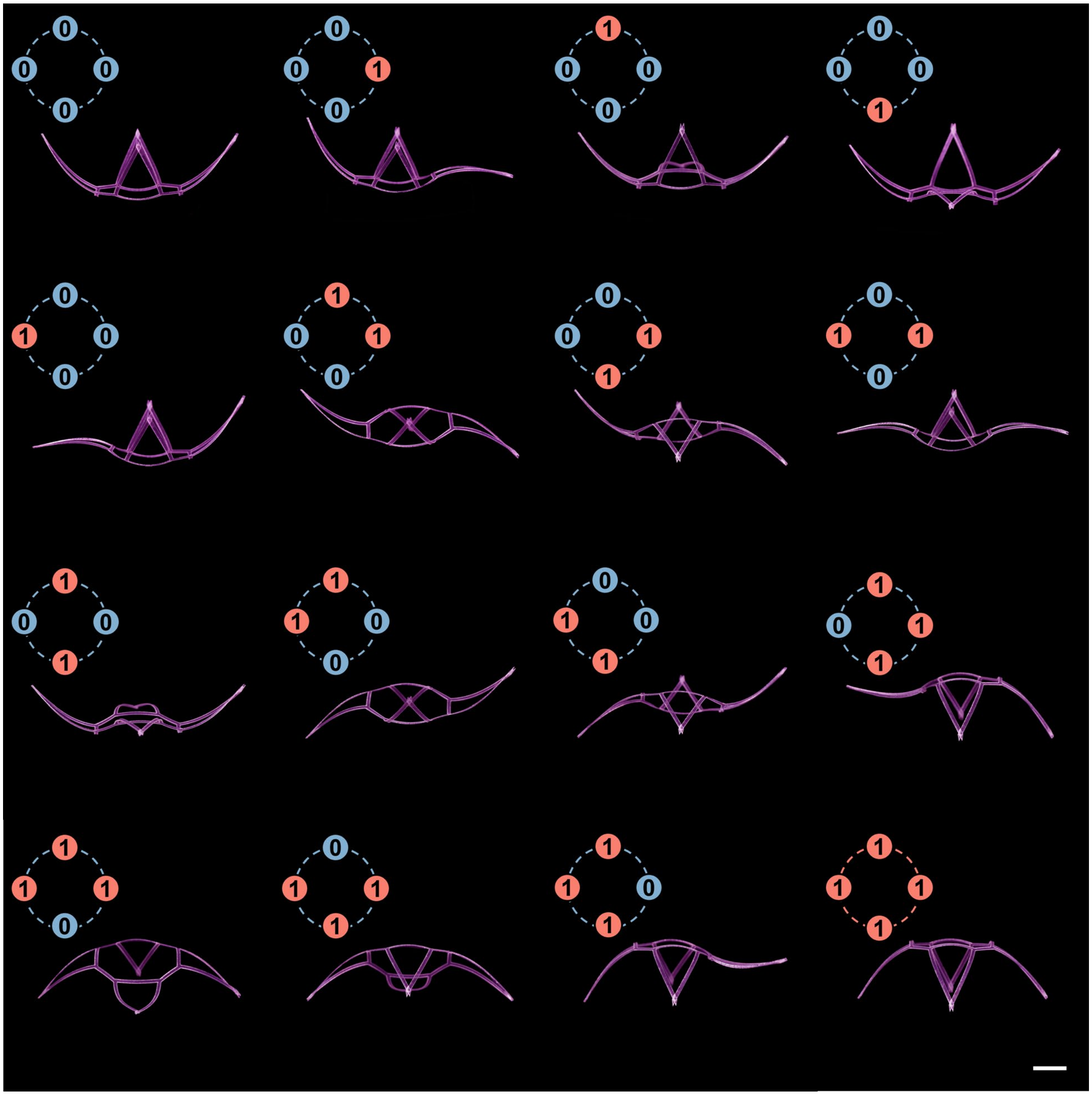

Figure S6. Experimental images illustrating the 16 stable states of the four-arm wing-like ribbon frame. Each arm of the frame exhibits bistability and can bend either upward (the "0" state) or downward (the "1" state), enabling switching between two stable configurations. The combination of the bistable states of the four arms gives rise to a total of 16 distinct stable states. Scale bar: 20 mm.

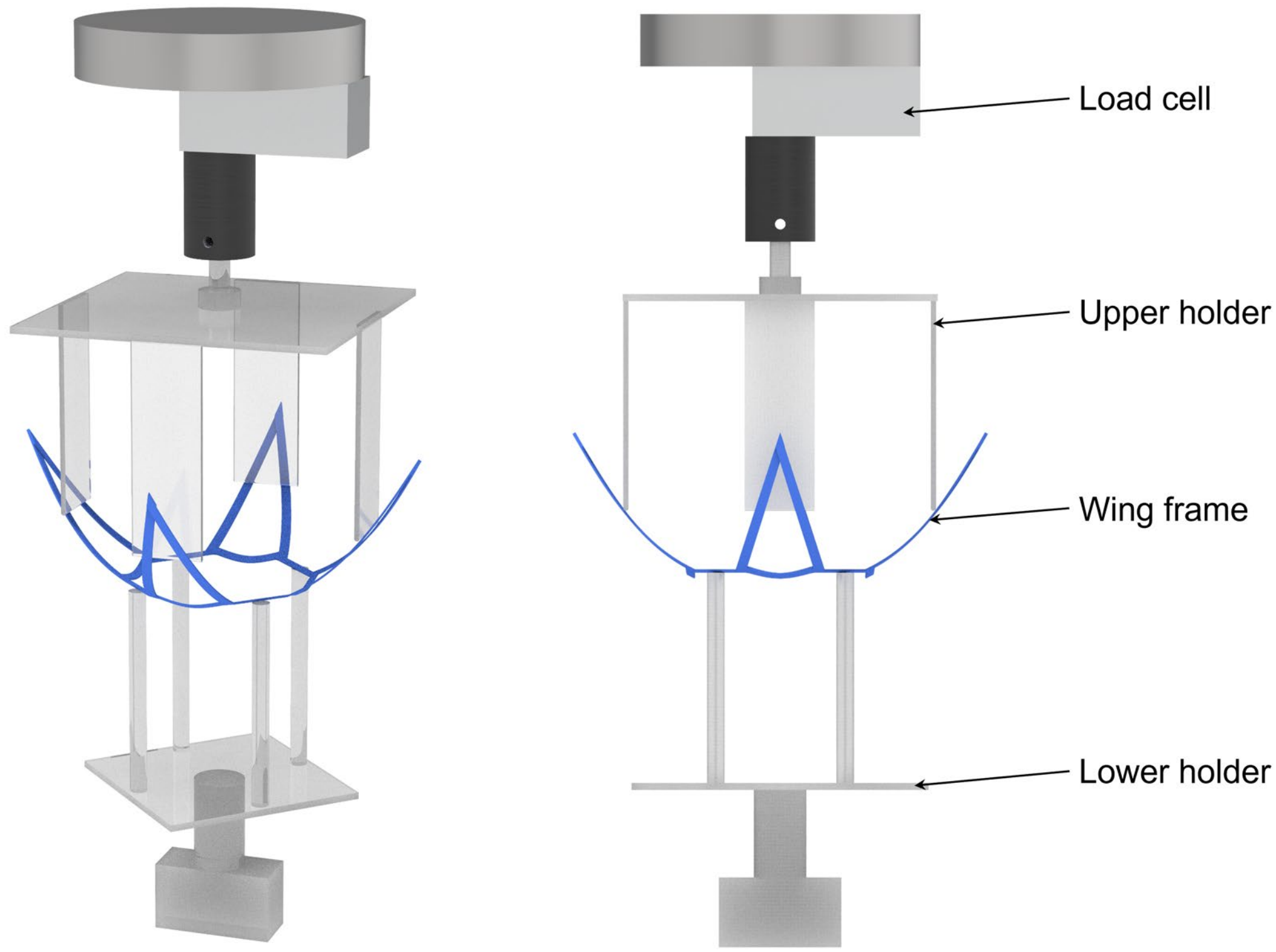

Figure S7. Setup for displacement-controlled force measurements. The wing frame is fixed with a upper holder and a lower holder. Upper holder is connected with the loadcell of Instron, and lower holder is connected with the pneumatic clamp.

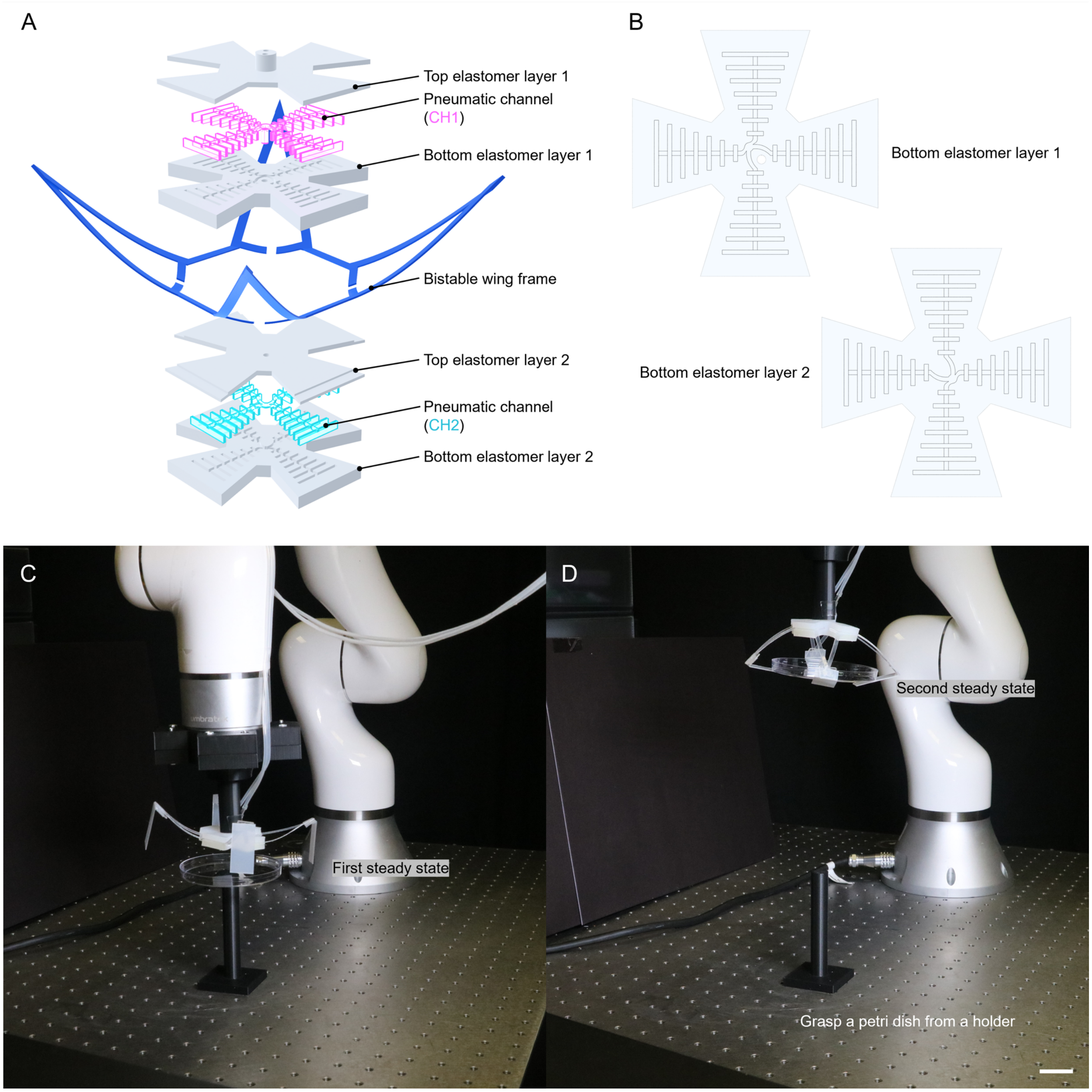

Figure S8. Demonstration of a bistable soft gripper using a bistable wing frame as the structural skeleton. The bistable wing frame is sandwiched between two soft bending actuators. (A) Exploded view of the bistable soft gripper, illustrating the assembly of the wing frame and actuators. (B) Configuration of the top and bottom elastomeric layers. (C) The bistable soft gripper mounted on a robotic arm for energy-efficient, gentle grasping. The gripper is actuated by two independently controlled pneumatic channels. Scale bar: 20 mm.

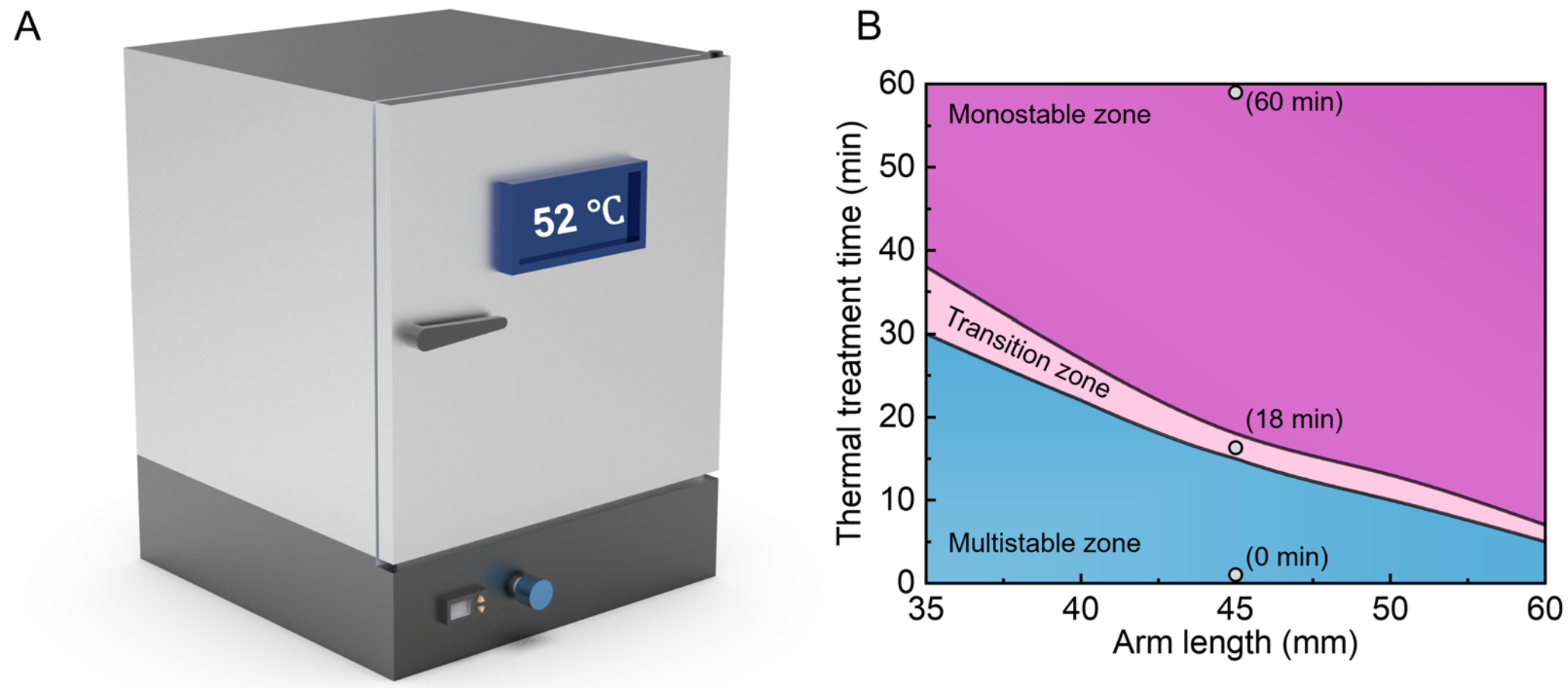

C

Type / Arm Length *AL*	Original	Cut	Thermal treated	Thermal treatment time	Temperature
35 mm	Multistable	Multistable	Monostable	30 ~ 38 min	52 ℃
40 mm	Multistable	Multistable		22 ~ 27 min	
45 mm	Multistable	Bistable		15 ~ 18 min	
50 mm	Multistable	Bistable		10 ~ 13 min	
60 mm	Monostable	Bistable		5 ~ 7 min	

Figure S9. Thermal treatment of the wing frame. (A) Thermal oven setup used for thermal treatment at 52 °C. (B) Phase diagram illustrating the transition from multistability to monostability in wing-like ribbon frames as a function of arm length *AL* and the thermal treatment time *T* at 52 °C. (C) Table summarizing the minimum thermal treatment time required to convert a multistable wing frame into a monostable configuration.

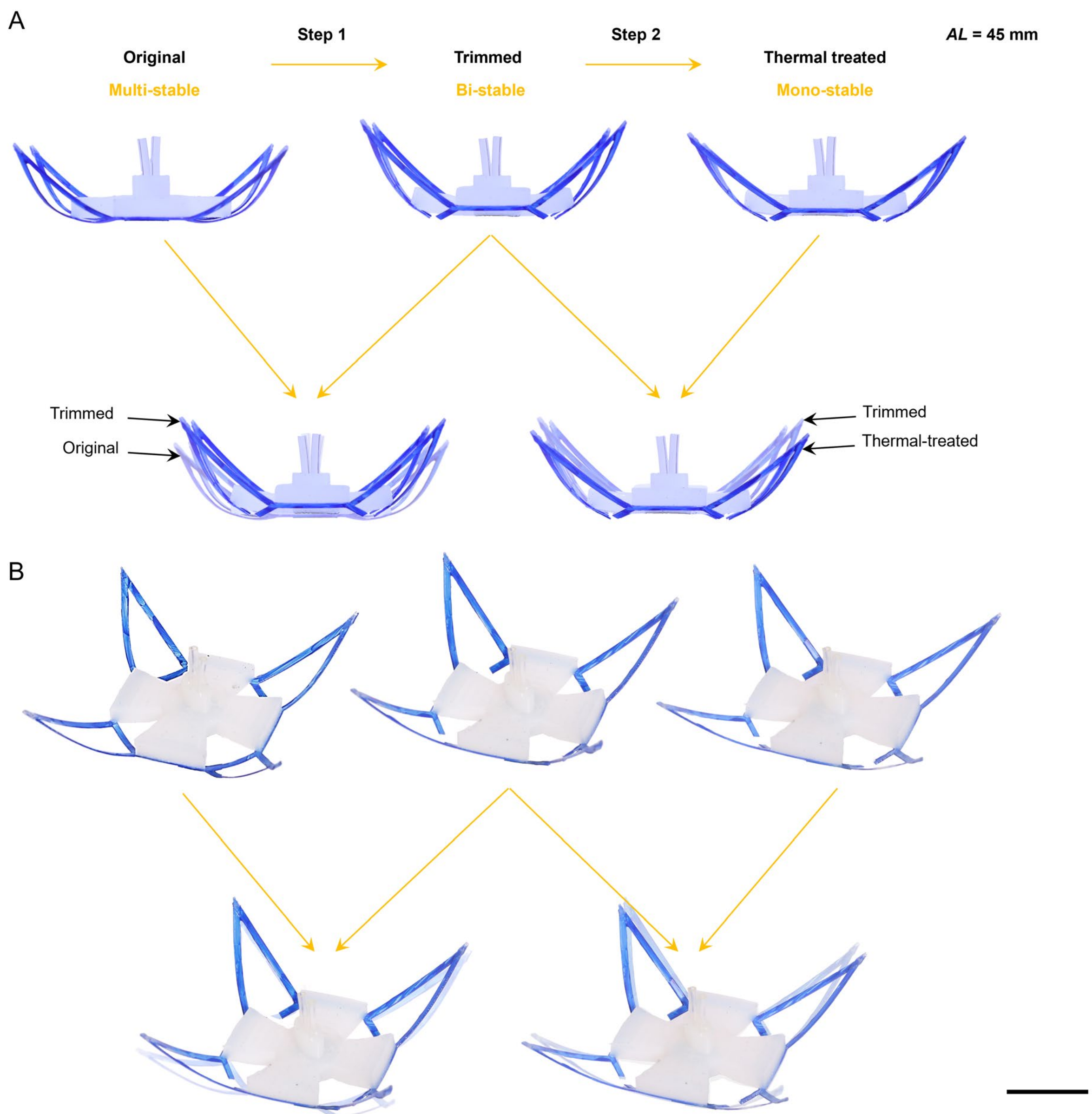

Figure S10. Shape comparison of original, trimmed, and thermal treated wing ribbon frames coupled with soft actuator (*AL* = 45 mm). (A) Front view. (B) Isometric view. Scale bar: 20 mm.

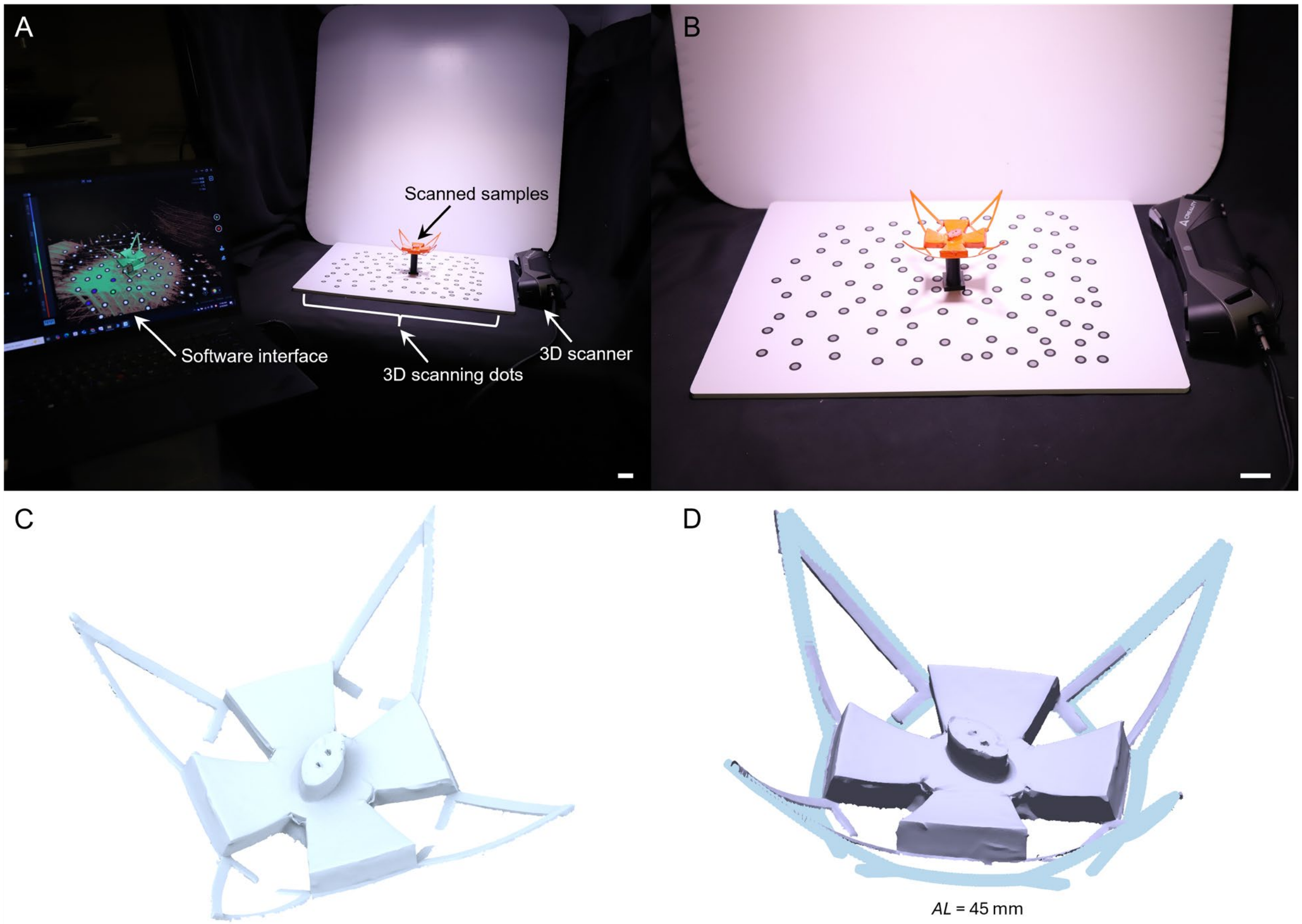

Figure S11. Setup of 3D Scanning and reconstruction process. (A) Setup. (B) Details. (C) Scanned images of a feather star-inspired soft actuator. (D) Comparison of analytical model and scanned model of a feather star-inspired soft actuator. Scale bar: 20 mm.

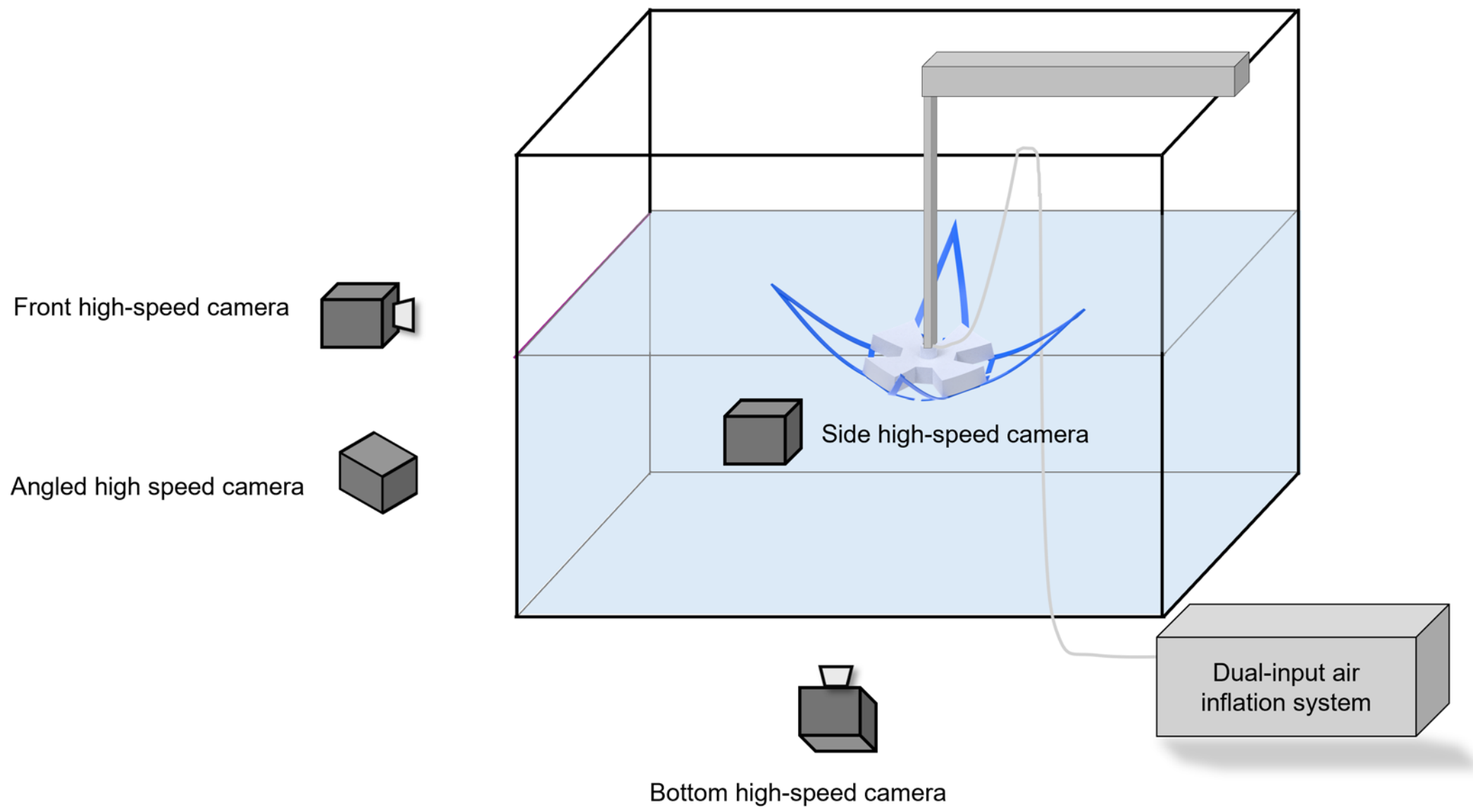

Figure S12. Experimental setup of high-speed camera captures for flapping motions of different actuation modes for feather-star soft swimmer.

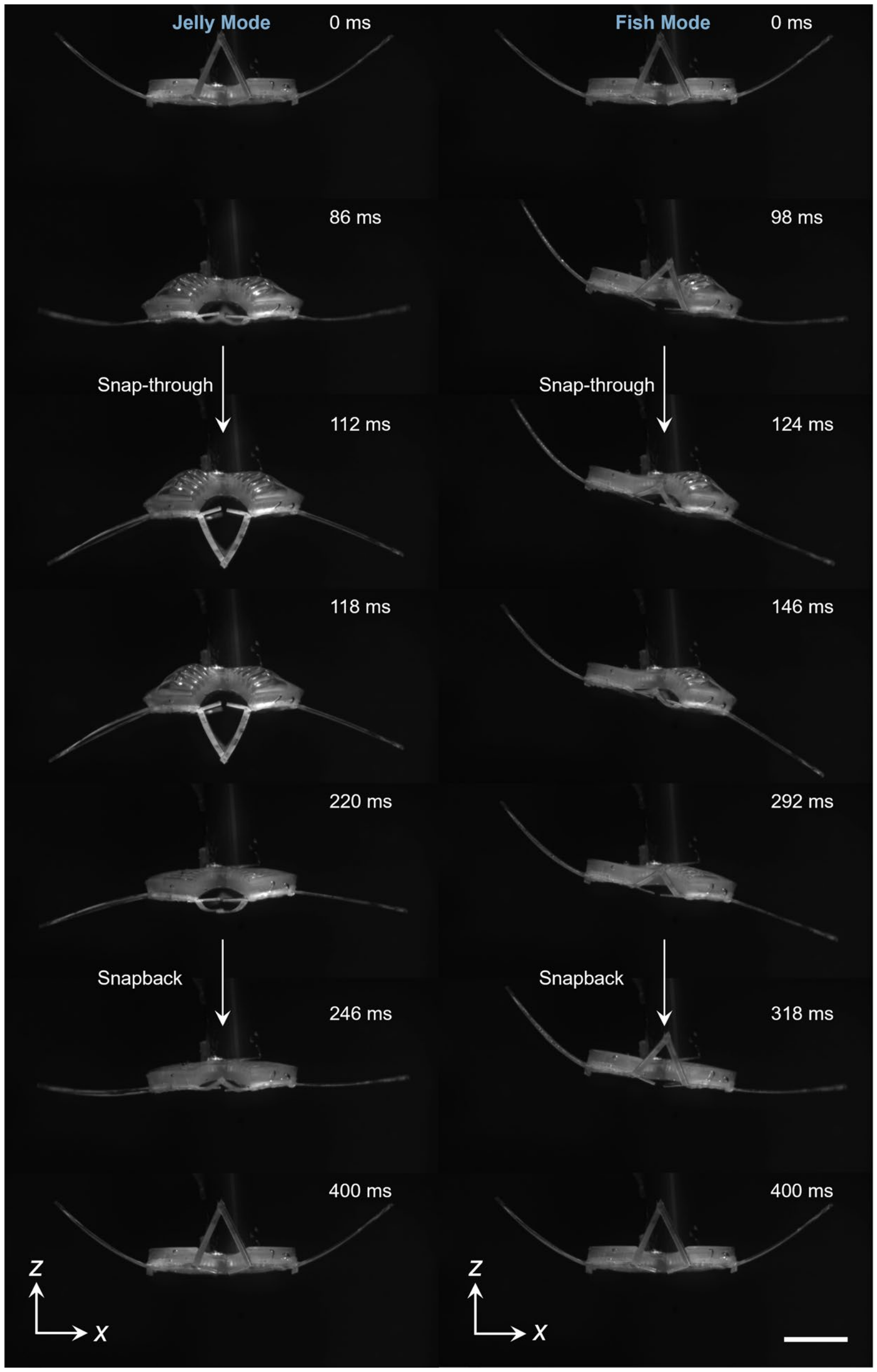

Figure S13. Time-lapse sequences showing one full flapping cycle in the Jelly and Fish Modes. Pneumatically actuated flapping motions during a representative downstroke–upstroke cycle are captured simultaneously from the front view (*xz* plane) using three synchronized high-speed cameras. The arm length is *AL* = 45 mm. Scale bar: 20 mm.

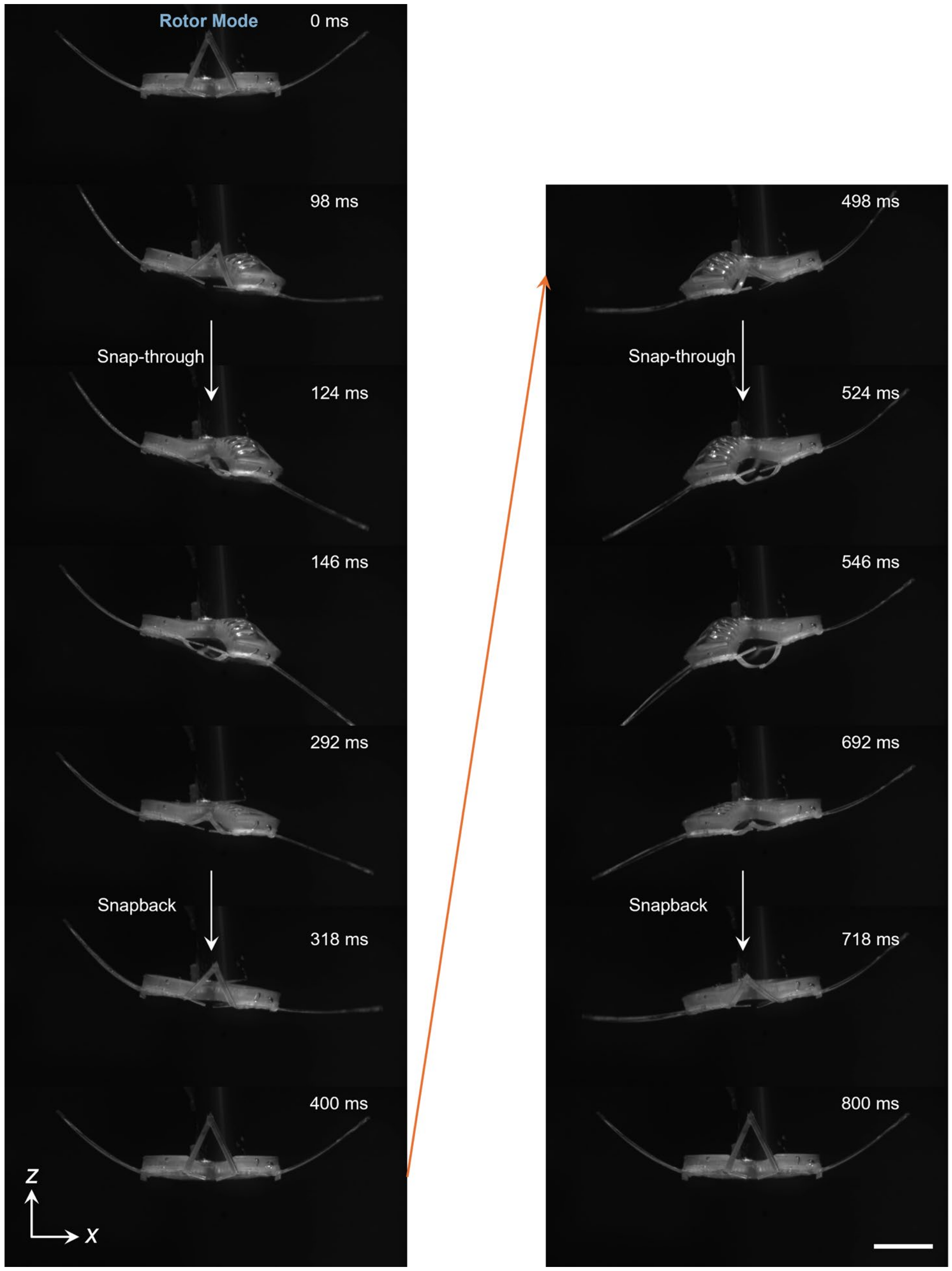

Figure S14. Time-lapse sequences showing one full flapping cycle in Rotor Mode. Pneumatically actuated flapping motions during a representative downstroke–upstroke cycle are captured simultaneously from front view (*xz* plane) using three synchronized high-speed cameras. The arm length is $AL = 45$ mm. Scale bar: 20 mm.

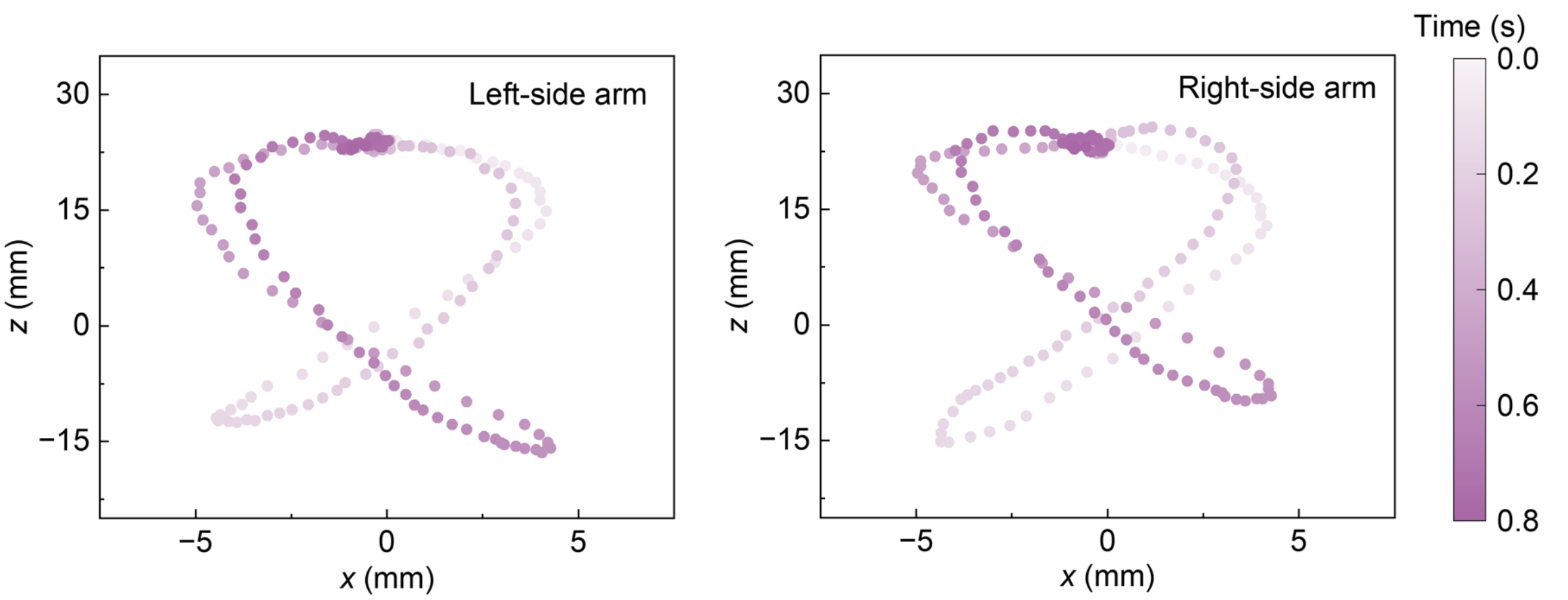

Figure S15. The tip trajectories of left-side and right-side arms during one full flapping cycle in the rotor mode actuated at 1.25 Hz (duration: 0.8 s). The trajectories of wing tips are captured at *xz* plane.

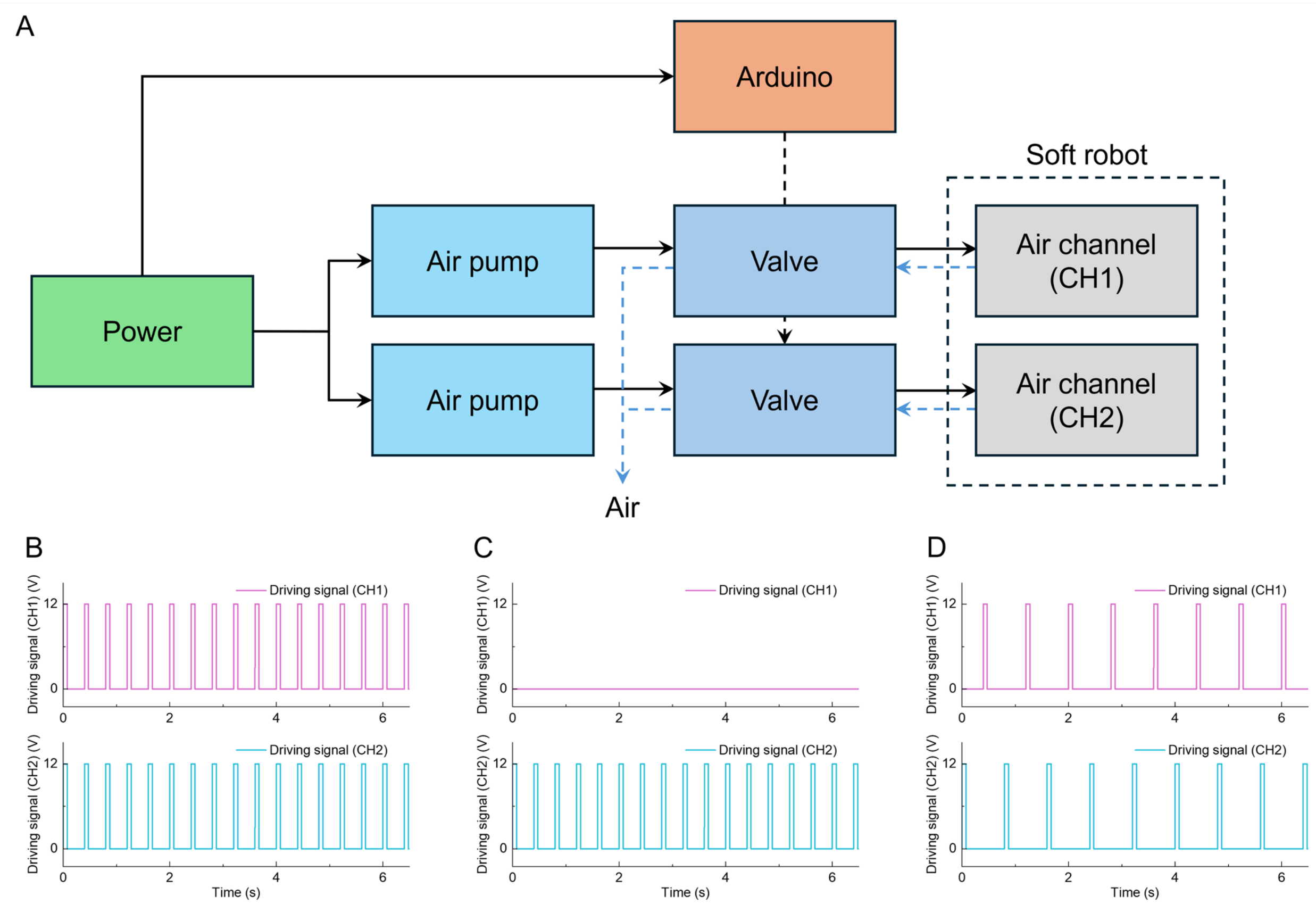

Figure S16. Actuation system for soft swimmers. (A) Diagram of the tethered actuation system. (B)-(D) The control signals of driving voltage for Jelly, Fish, and Rotor Modes, respectively.

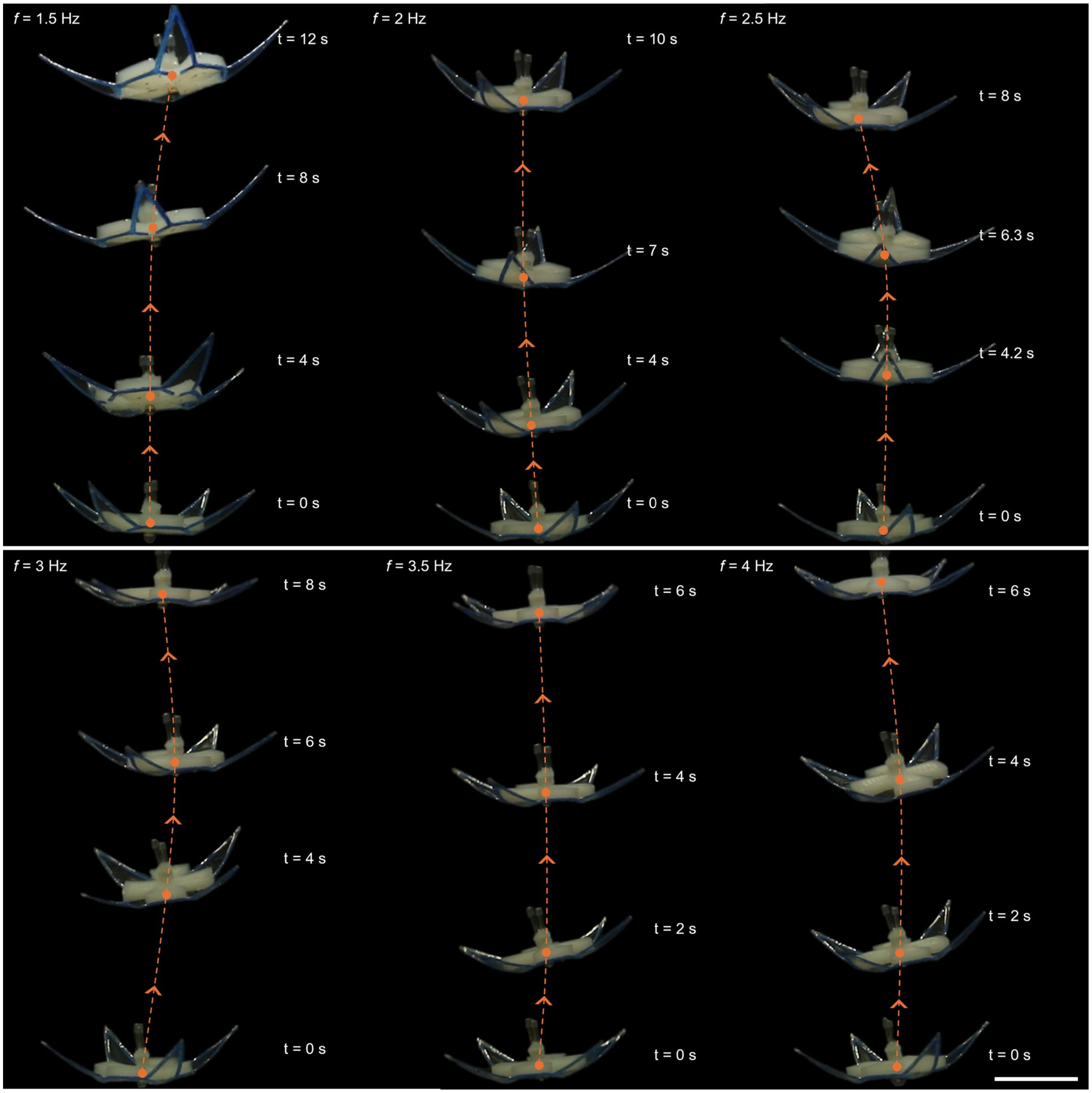

Figure S17. Time-lapse images of vertical ascent for soft swimmer actuated at Jelly Mode under different actuation frequencies starting from 1.5 Hz to 4 Hz. Scale bar: 50 mm.

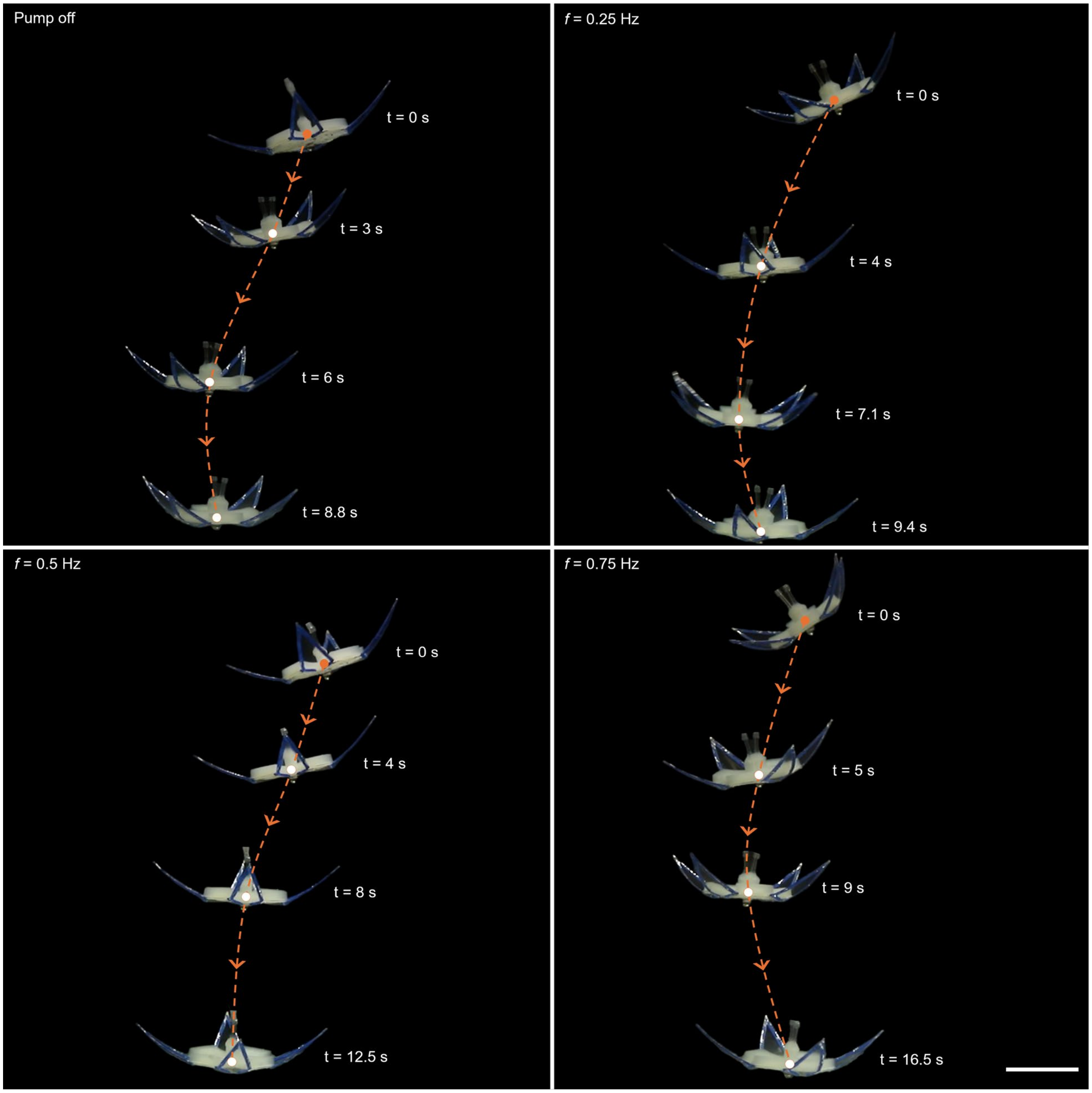

Figure S18. Time-lapse images of free descent for soft swimmer actuated at Jelly Mode under different actuation frequencies starting from 0 Hz to 0.75 Hz. Scale bar: 50 mm.

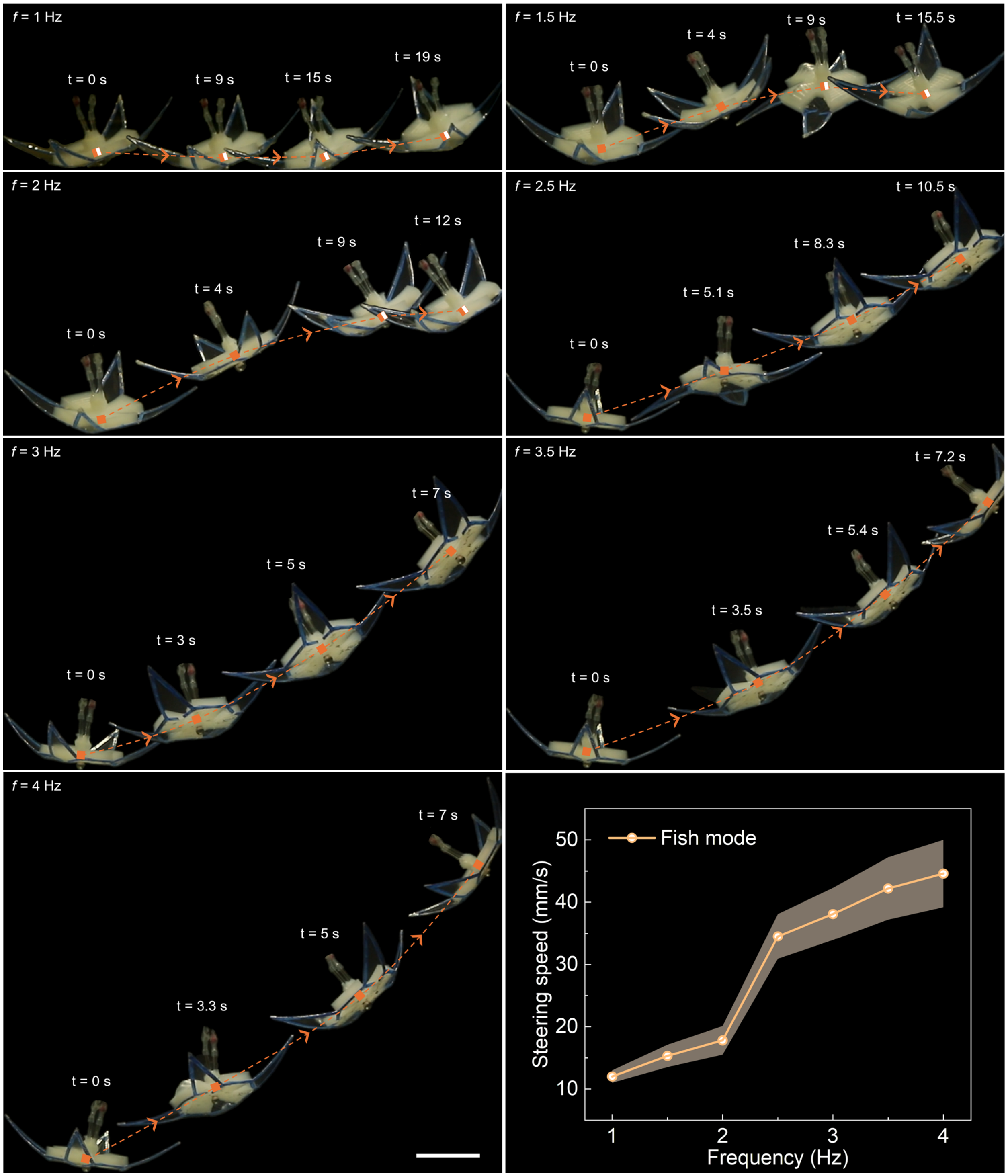

Figure S19. Time-lapse images illustrating rightward steering of the soft swimmer actuated in Fish Mode at different actuation frequencies ranging from f_{CH1} = 1 to f_{CH1} = 4 Hz. Directional steering is achieved by frequency-controlled fish-mode flapping, with representative trajectories shown for each actuation condition. The bottom-right panel quantifies the steering speed as a function of actuation frequency in Fish Mode. Scale bar: 50 mm.

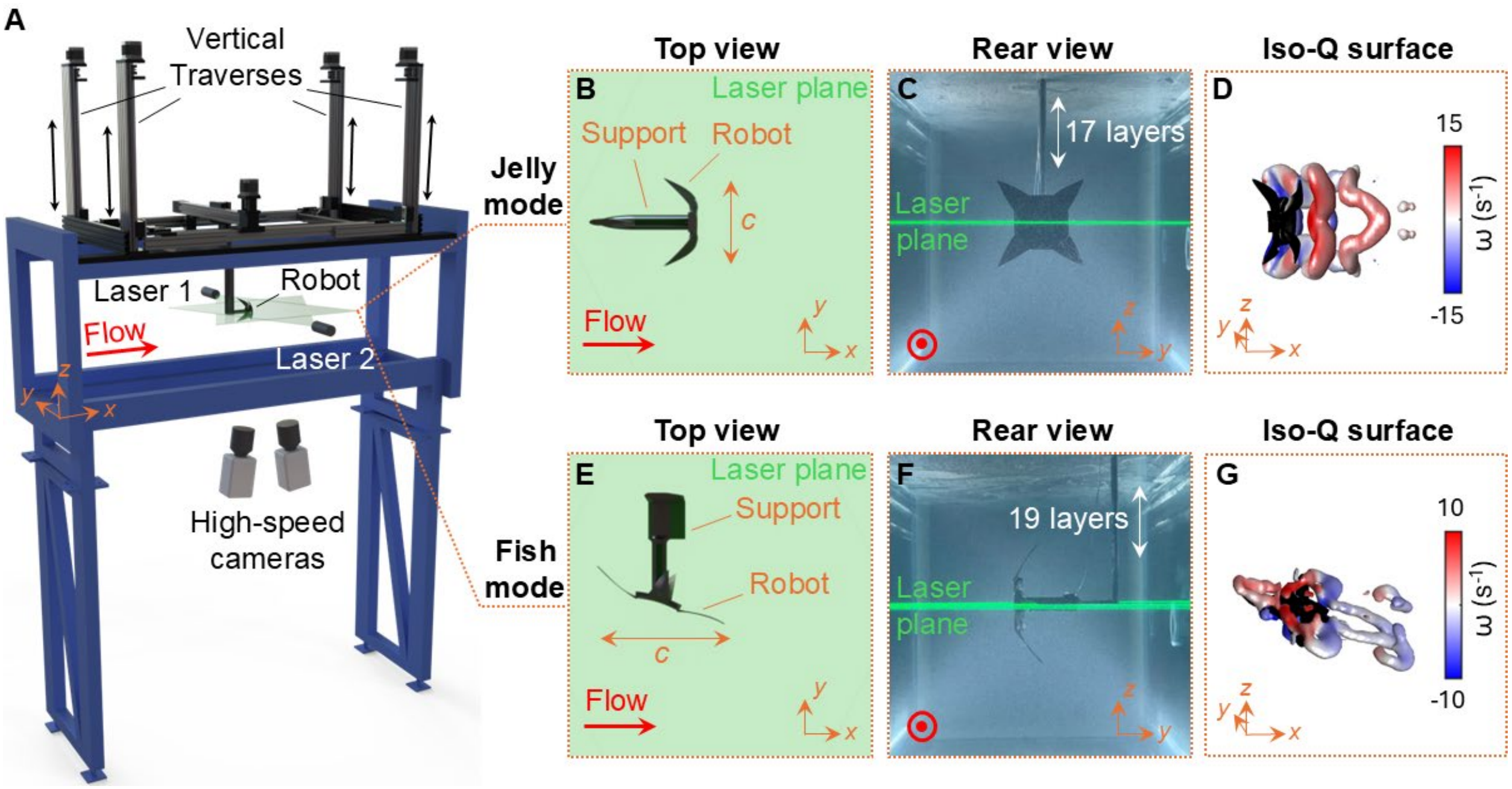

Figure S20. Multi-layer stereoscopic particle image velocimetry (SPIV) setup. (A) Water channel test section. (B, C, D) Top view, rear view, and 3D wake structures of the jelly mode. (E, F, G) Top view, rear view, and 3D wake structures of the fish mode.

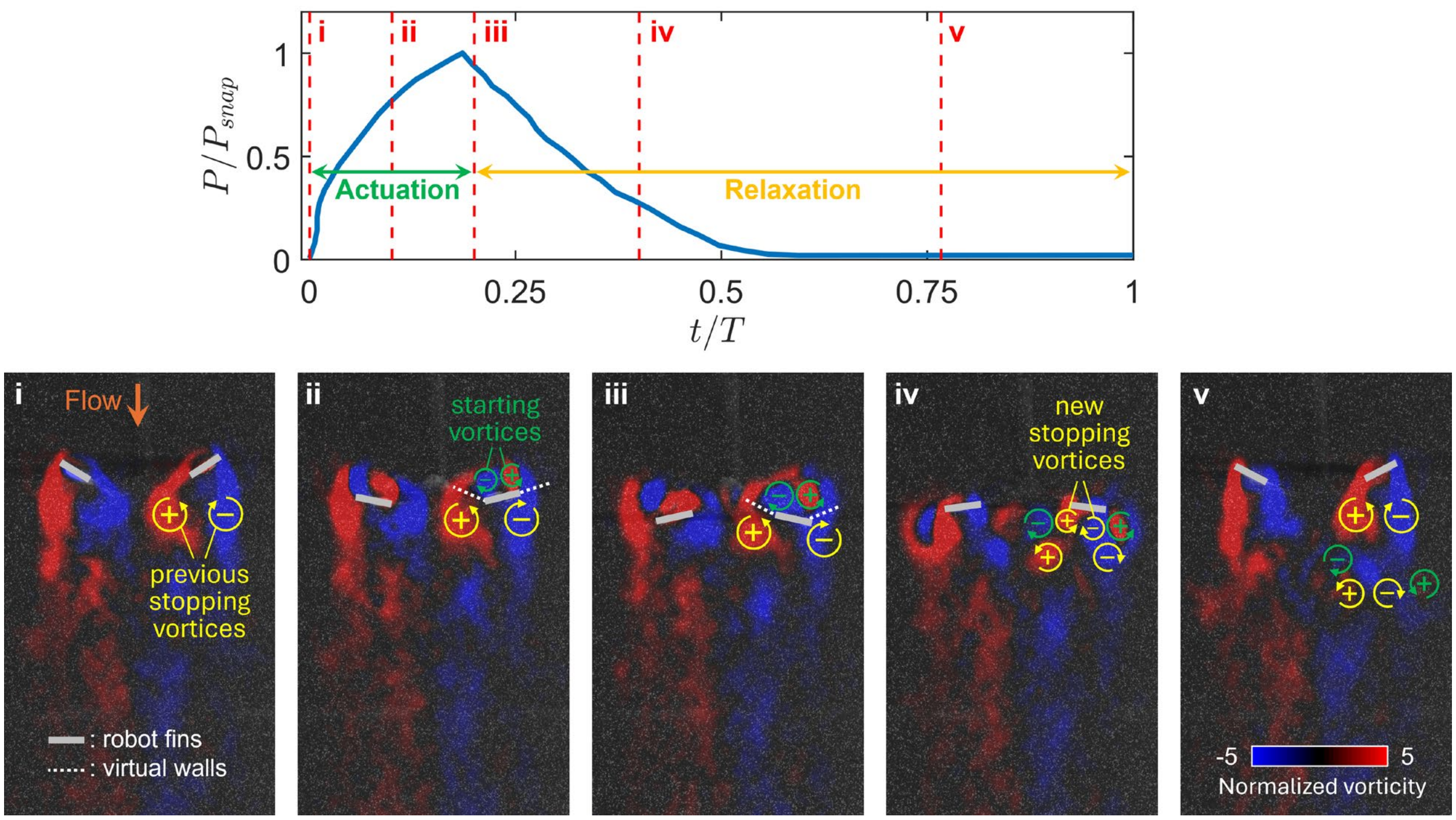

Figure S21. Flow analysis of Jelly Mode over one actuation cycle. Top panel: Time-lapse of the pressure signal replotted from Fig. 3A. (i–v) Vortex dynamics associated with Jelly-Mode propulsion over one actuation cycle. Only the vortices of the right fin are annotated for brevity.

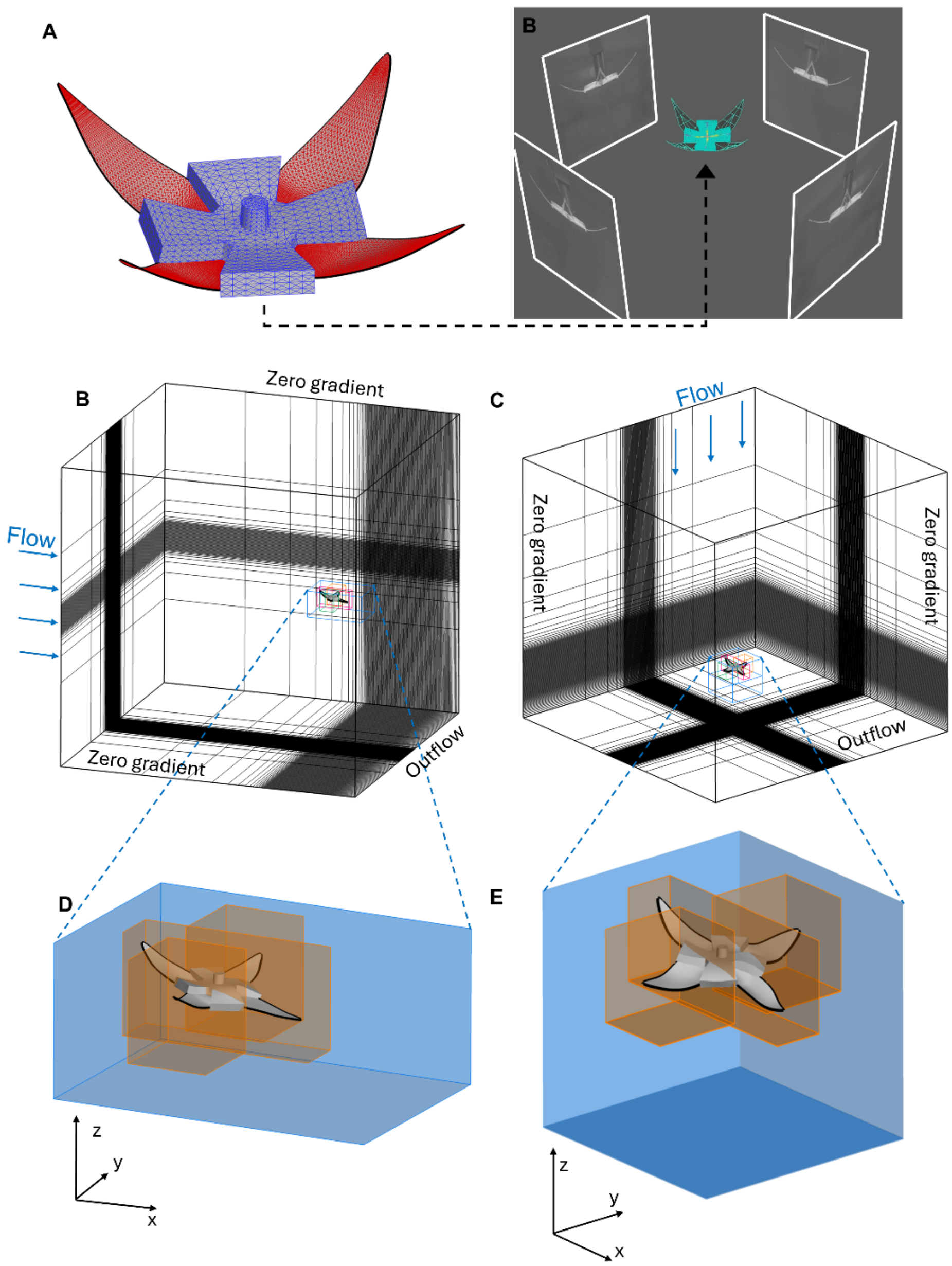

Figure S22. Computational kinematic reconstruction and computational fluid dynamics (CFD) setup. (A) The computational swimmer model. (B) The virtual scene setup for kinematic reconstruction. (B, C) CFD domain setup for flow simulations of the fish and rotor modes, respectively. (D, E) Nested tree-topological local mesh refinement (TLMR) of the near field.

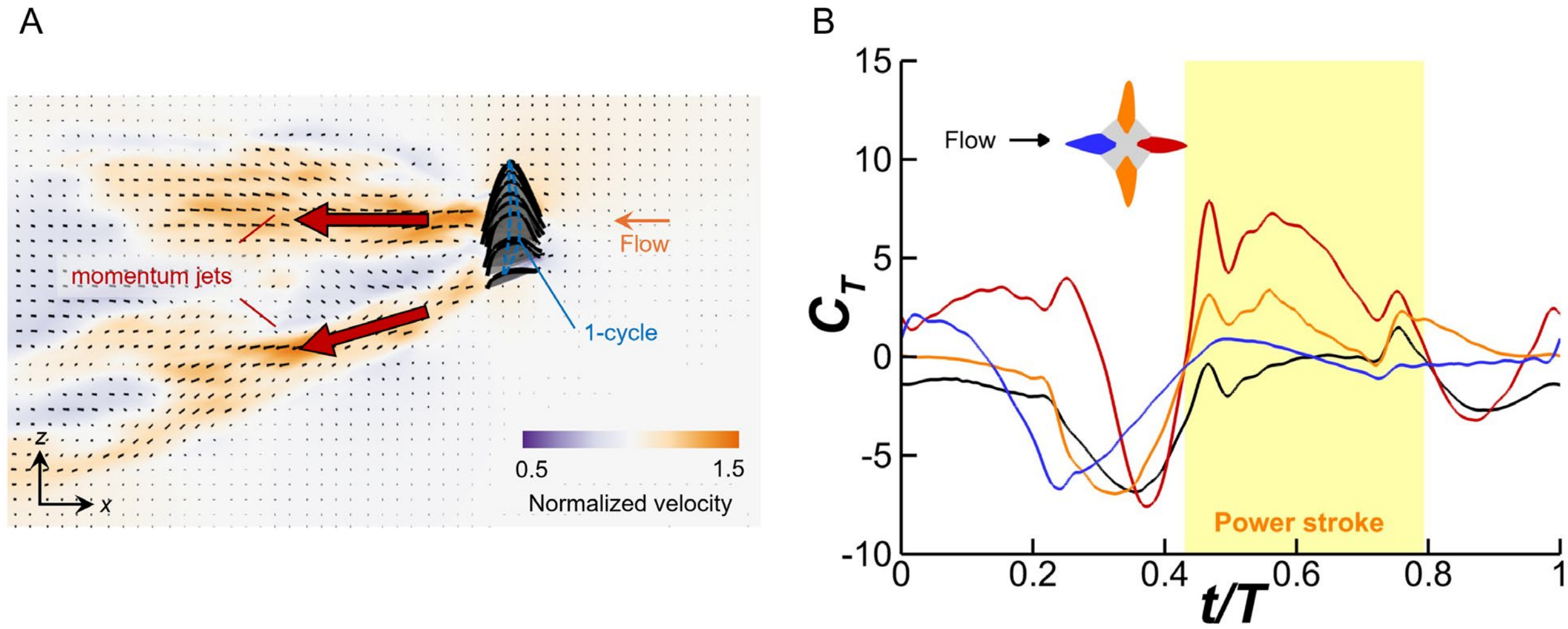

Figure S23. A, The CFD simulation of Fish Mode, showing the streamwise momentum on sagittal cross section of the flow field intersecting the side fin, a quarter-span from the fin tip. B, Instantaneous thrust coefficient of the body and the arms in Fish Mode. $C_T = F_T/q_\infty S_A$, where F_T is the force component opposite of the flow direction, q_∞ is the water dynamic pressure at room temperature, S_A is the average surface area of an arm. Black line is for the body, and each of the color corresponds to an arm with the same color as shown on the top left.

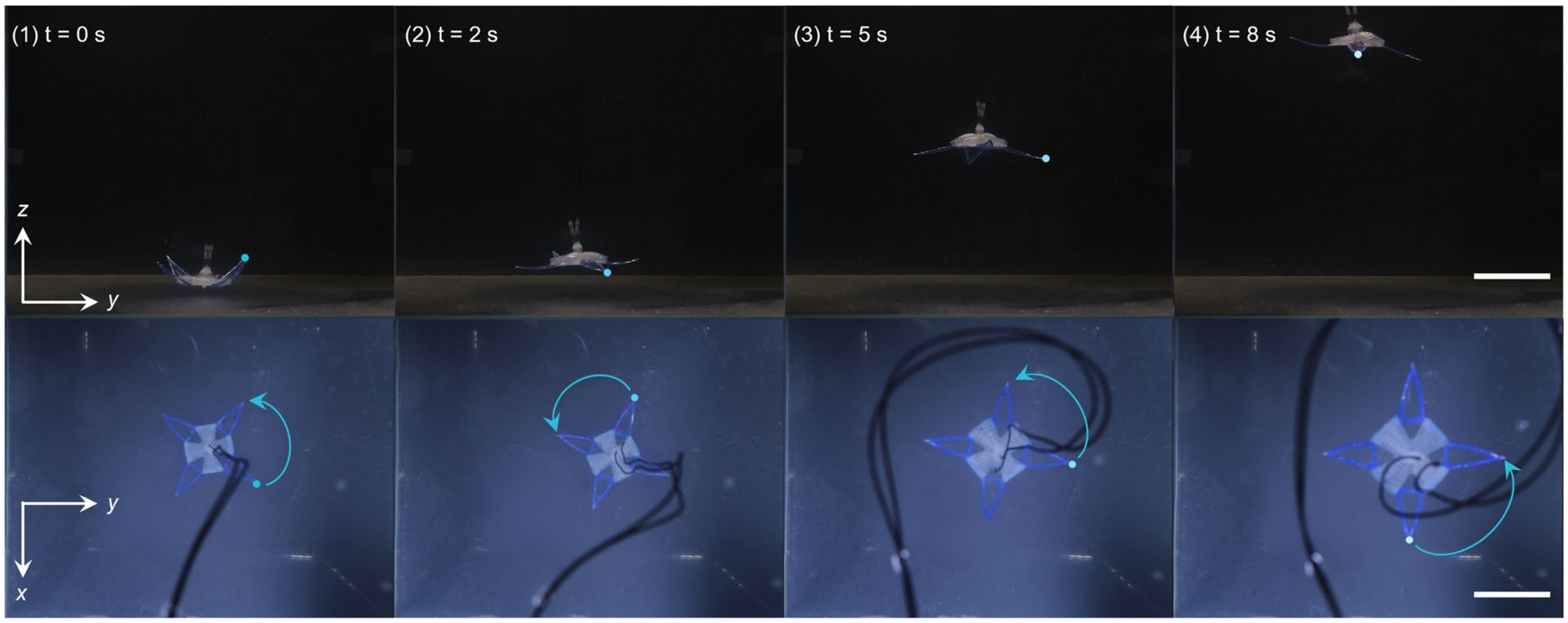

Figure S24. Characterization of the Rotor Mode: time-lapse front- and top-view images showing counterclockwise rotational ascent (*AL* = 45 mm, 1.52 Hz). Scale bar, 50 mm.

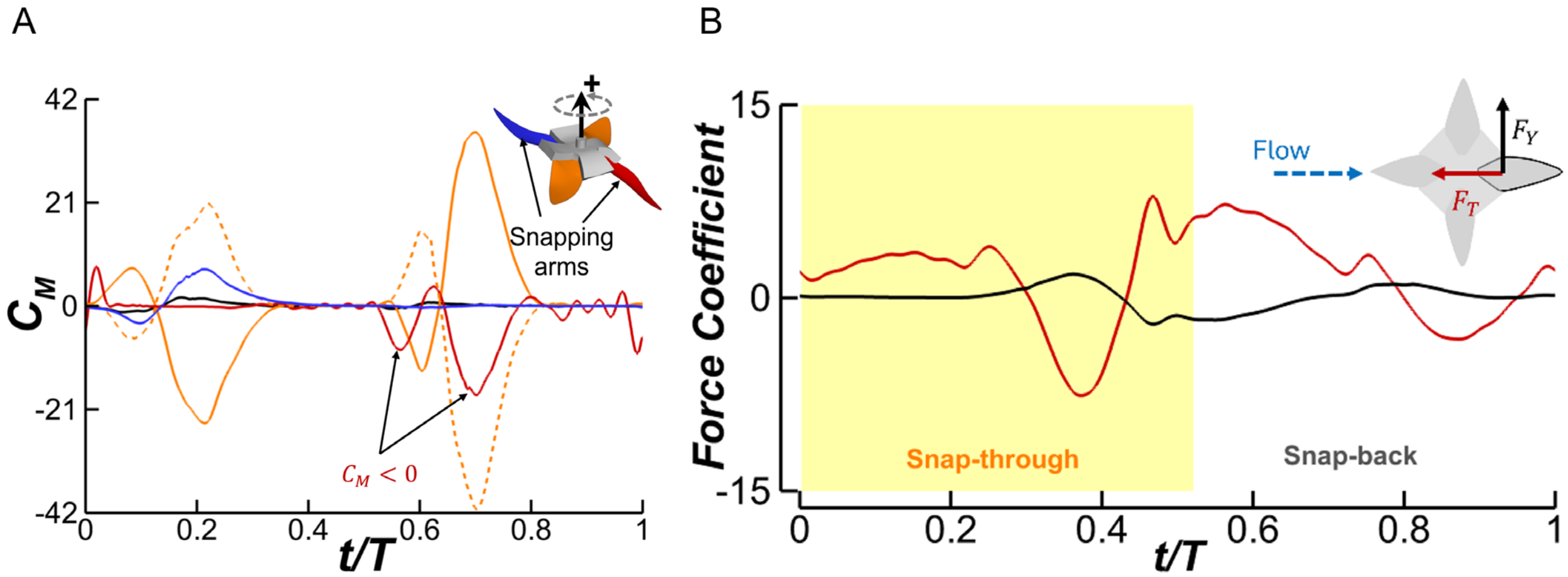

Figure S25. A, Instantaneous torque coefficient of the body and the arms in Rotor Mode. $C_M = M/q_\infty S_A l$, where M is the torque (positive being counterclockwise when viewed from the top), q_∞ is the water dynamic pressure at room temperature, S_A is the average surface area of an arm, and l is the length of the swimmer from one arm tip to the opposite arm tip. Black line is for the body, and each of the color corresponds to an arm with the same color as shown on the top left. The dashed orange arm represents the one that points out of the page from the body. B, Propulsive (red) and lateral (black) force coefficients on the trailing wing in the fish mode over a cycle of motion. $C_{T/Y} = F_{T/Y}/q_\infty S$, where F_T is the force component opposite of the flow direction, F_Y is the lateral component, q_∞ is the dynamic pressure of the flow, and S is the surface area of the wing.

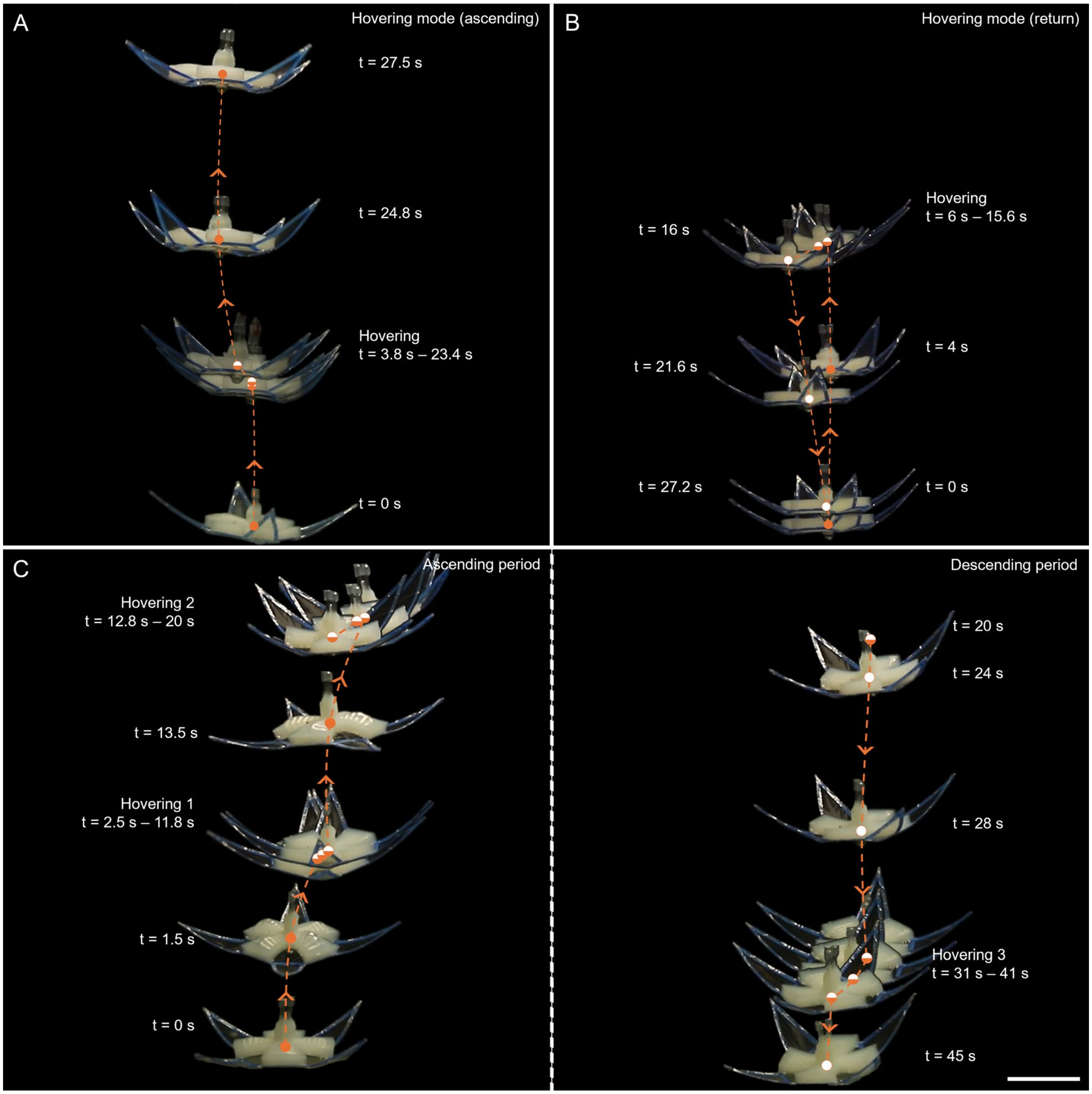

Figure S26. Time-lapse images demonstrating hovering depth control of the soft swimmer actuated in Jelly Mode at different actuation frequencies. (A) Starting from the water bottom, the soft swimmer ascends to an intermediate depth by t = 3.8 s under the jellyfish mode ($f_{CH1} = f_{CH2}$ = 2.5 Hz). It then switches to the hovering mode ($f_{CH1} = f_{CH2}$ = 1 Hz) and maintain its position at that depth until t = 23.4 s. From t = 23.4 s to 27.5 s, the swimmer resumes ascending motion to a shallower depth under Jelly Mode. (B) Starting from the bottom, the swimmer ascends to a target depth by t = 6 s under Jelly Mode ($f_{CH1} = f_{CH2}$ = 2.5 Hz), then switches to the hovering mode ($f_{CH1} = f_{CH2}$ = 1 Hz) and remains at that depth until t = 15.6 s. By reducing the actuation frequency to $f_{CH1} = f_{CH2}$ = 0.5 Hz in Jelly Mode, the swimmer descends and returns to its initial deeper position by t = 27.2 s. (C) Starting from the bottom, the swimmer ascends to an intermediate depth by t = 2.5 s under Jelly Mode ($f_{CH1} = f_{CH2}$ = 2.5 Hz), then switches to the hovering mode ($f_{CH1} = f_{CH2}$ = 1

Hz) and maintains that depth until t = 11.8 s. It subsequently ascends to a shallower depth from t = 11.8 s to t = 12.8 s under Jelly Mode, followed by another hovering phase at that depth until t = 20.0 s. When actuated at a lower frequency ($f_{CH1} = f_{CH2}$ = 0.5 Hz), the swimmer descends and then stabilizes at an intermediate depth between t = 31.0 s to t = 41.0 s. Finally, it returns to the initial starting point by t = 45.0 s under lower-frequency Jelly Mode actuation. Scale bar: 50 mm.

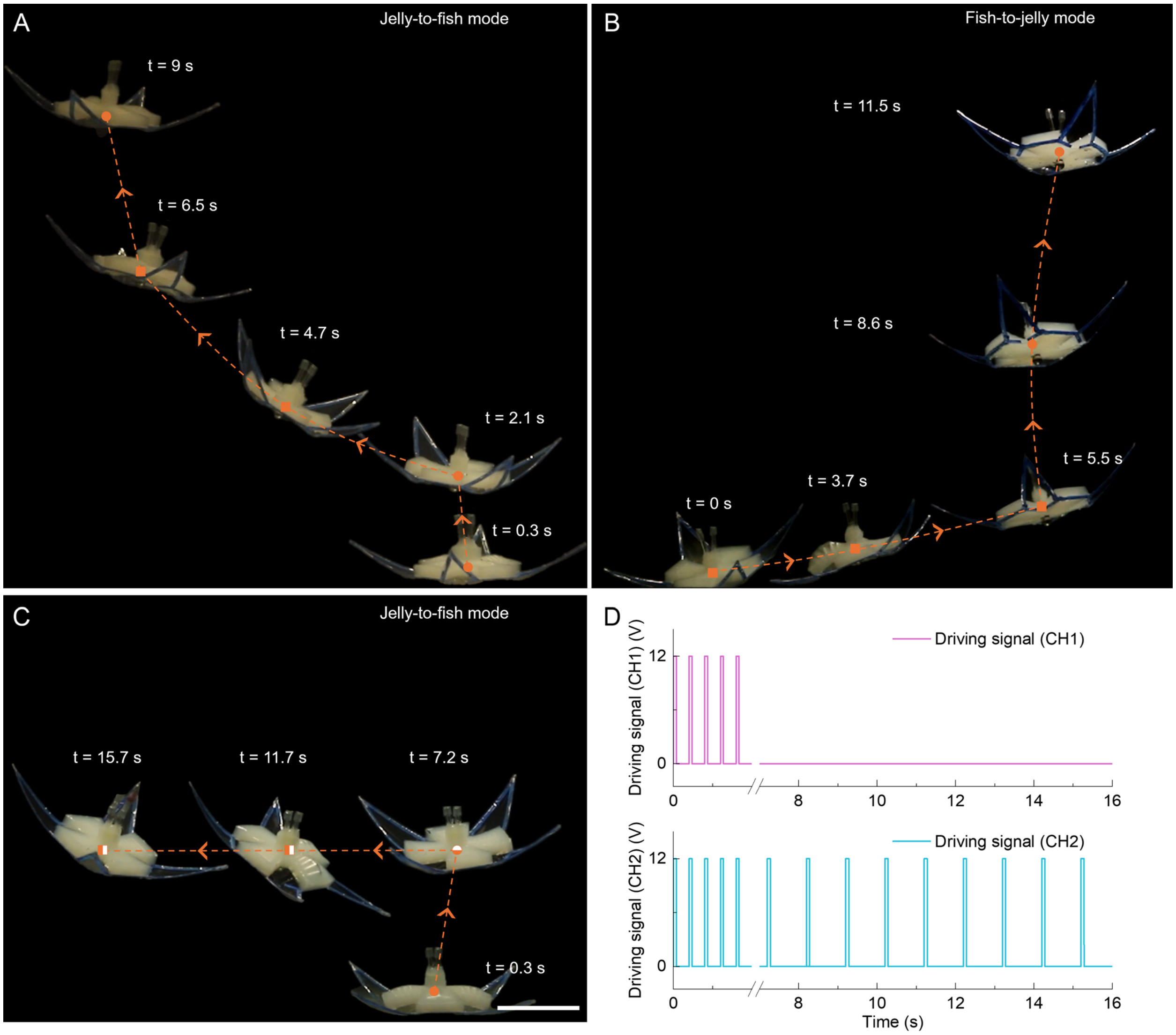

Figure S27. Diverse complex swimming trajectories demonstrating continuous transitions among Jelly and Fish Modes. (A) Jelly-to-Fish transition. Starting from the water bottom, the soft swimmer ascends vertically in Jelly Mode ($f_{CH1} = f_{CH2}$ = 2.5 Hz) and reaches an elevated position by t = 2.1 s. It then transitions to leftward steering in Fish Mode (f_{CH1} = 0 Hz; f_{CH2} = 4 Hz) by t = 6.5 s. Subsequently, the swimmer switches back to Jelly Mode and resumes vertical ascent to a shallower depth by t = 9.0 s. (B) Fish-to-Jelly transition. Starting from the bottom, the swimmer first performs rightward steering with a shallow trajectory slope in Fish Mode (f_{CH1} = 0 Hz; f_{CH2} = 1.5 Hz) and reaches a lateral position by t = 5.5 s. It then transitions to Jelly Mode ($f_{CH1} = f_{CH2}$ = 2.5 Hz) and ascends vertically to a shallower depth by t = 11.5 s. (C)-(D) Jelly-to-Fish transition and corresponding driving signals. Starting from the bottom, the swimmer ascends vertically in Jelly Mode ($f_{CH1} = f_{CH2}$ = 1.5 Hz) and reaches an elevated position by t = 7.2 s. It then transitions to leftward horizontal steering in Fish Mode (f_{CH1} = 0 Hz; f_{CH2} = 1 Hz) by t = 15.7 s. (D) shows the corresponding driving voltage signals used to achieve the mode transition. Scale bar: 50 mm.

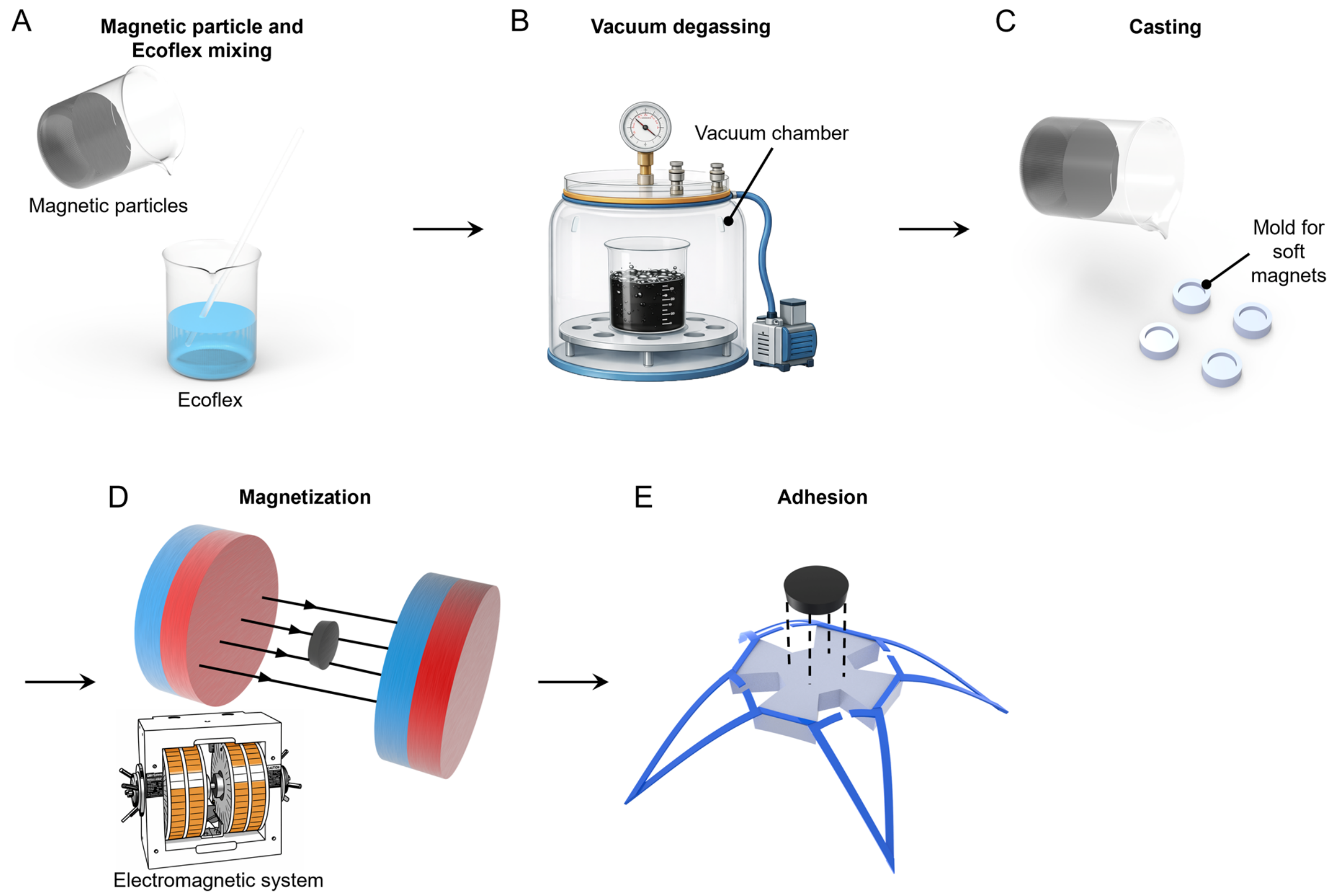

Figure S28. Fabrication procedure of the soft magnet integrated with a soft bending actuator. (A) Mixing Ecoflex with magnetic particles to form a homogeneous magnetic elastomer. (B) Vacuum degassing to remove trapped air bubbles from the magnetic mixture. (C) Casting and curing of the soft magnet by pouring the magnetic mixture into molds. (D) Magnetization of the cured soft magnets using an electromagnetic system. (E) Bonding the soft magnet to the bottom surface of the elastomeric soft actuator using a silicone rubber adhesive.

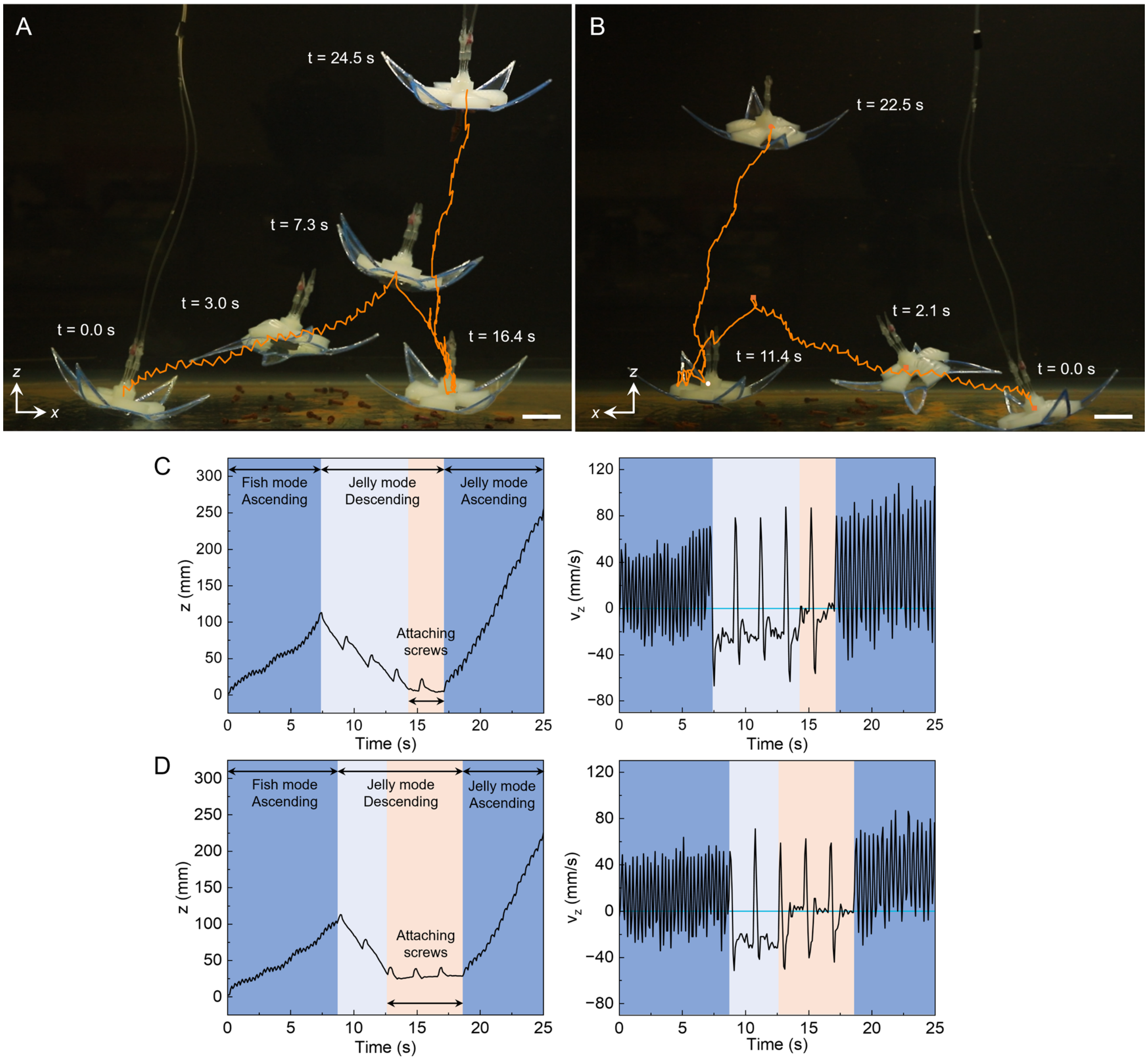

Figure S29. Corresponding experimental trajectories of the soft swimmer integrated with a soft magnetic pad. (A, C) Measured planar trajectories and the corresponding displacement and velocity along the z-axis as functions of time for the case shown in Figure 5B. (B, D) Measured planar trajectories and the corresponding displacement and velocity along the z-axis as functions of time for the case shown in Figure 5C. Scale bar: 20 mm.

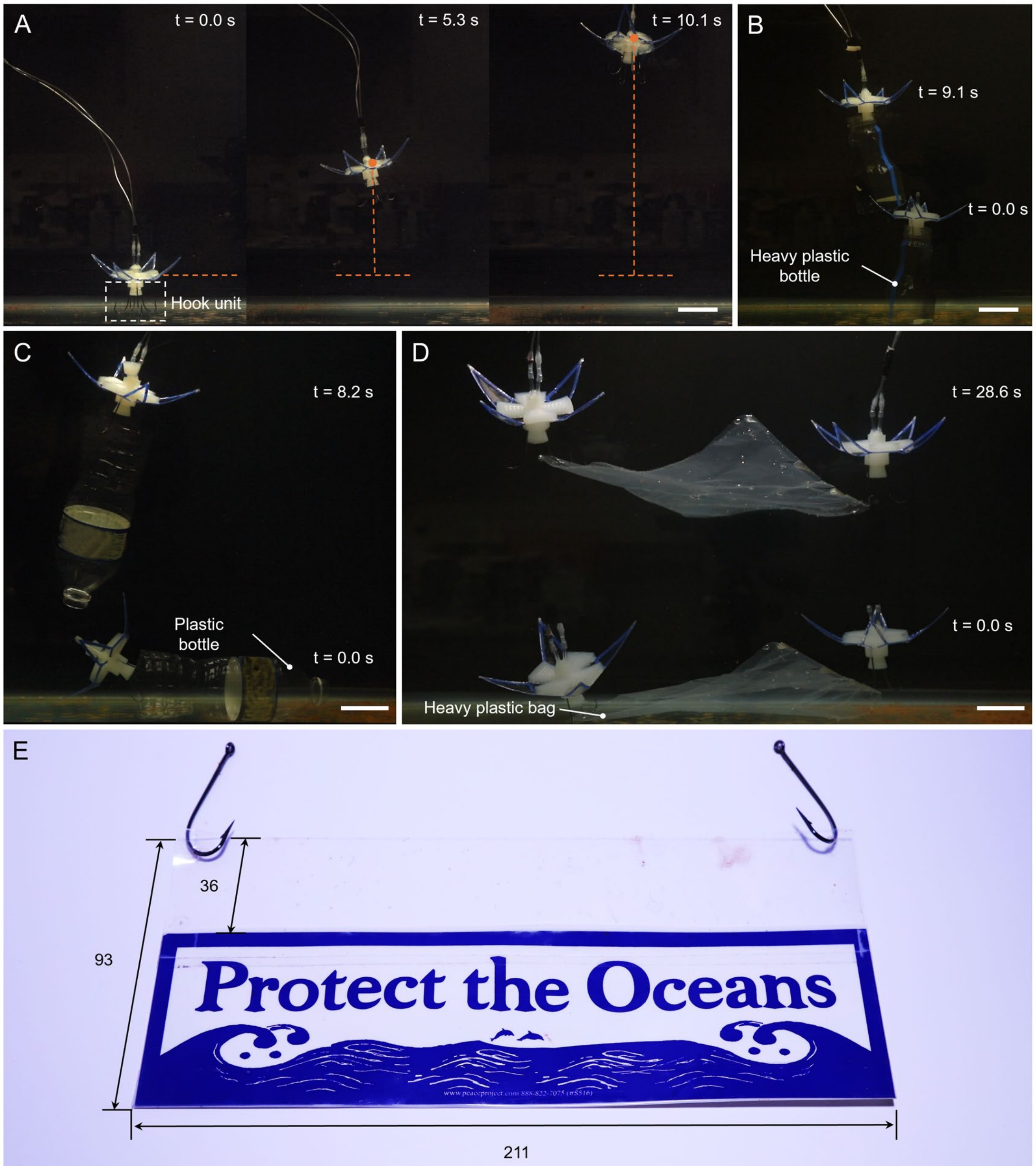

Figure S30. Plastic waste collection using integrated hooks and cooperative operation. (A) Vertical ascent performance of a soft swimmer integrated with four hooks and a buoyancy unit. (B) Demonstration of lifting a heavy plastic bottle with a single swimmer. (C) Demonstration of lifting a large plastic bottle with a single swimmer. (D) Demonstration of lifting a heavy plastic bag using two cooperative swimmers. (E) The real dimension of a large sticker used in Figure 5F. Scale bar: 50 mm.

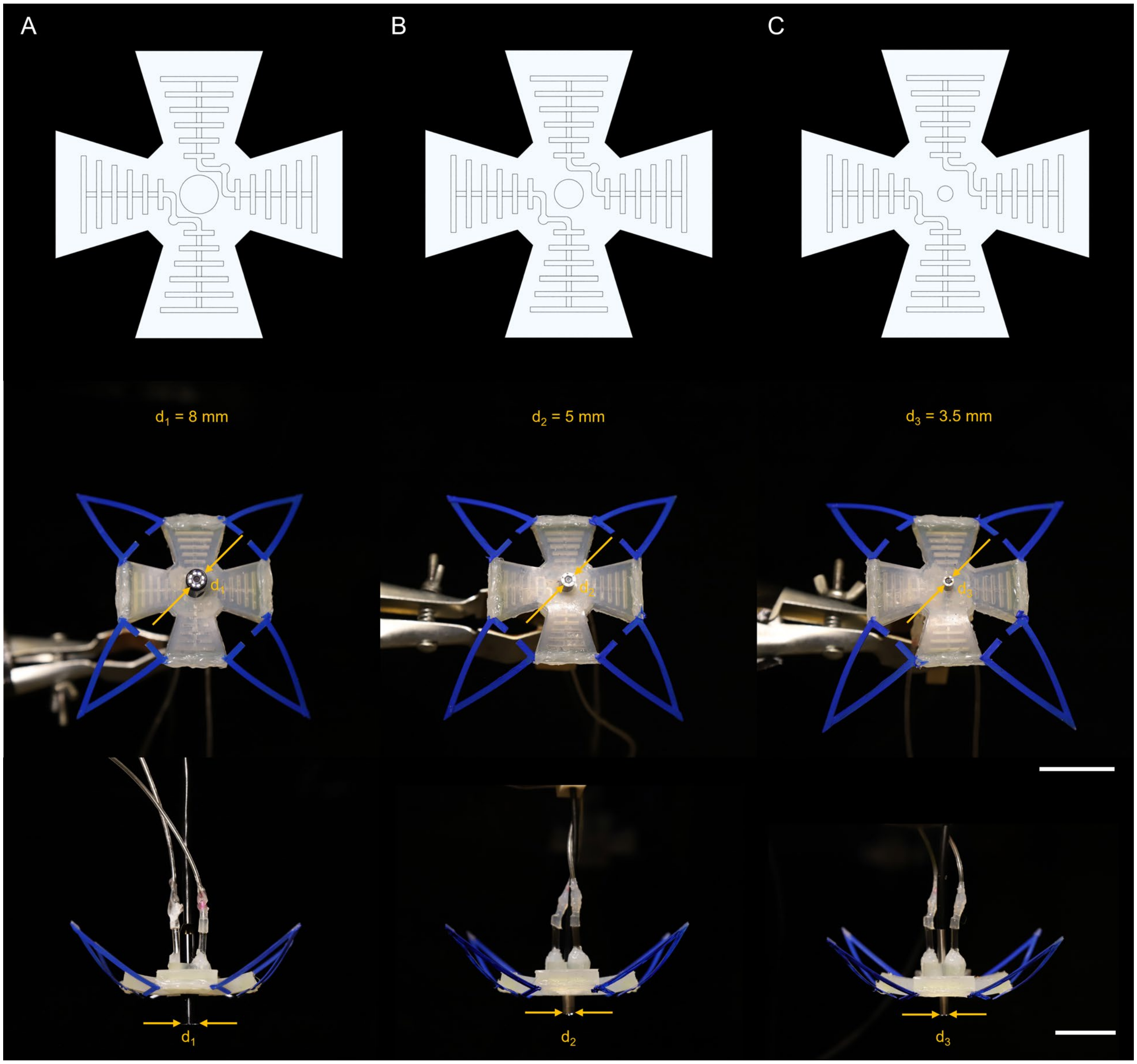

Figure S31. Schematics and optical images of soft swimmers mounted with miniature cameras of different diameters. (A) Actuator design for mounting a miniature camera with diameter d_1 = 8 mm, along with bottom and front views of the swimmer with the camera mounted. (B)-(C) Corresponding actuator designs and assembled views for miniature cameras with diameters d_2 = 5 mm and d_3 = 3.5 mm, respectively. Scale bar: 20 mm.

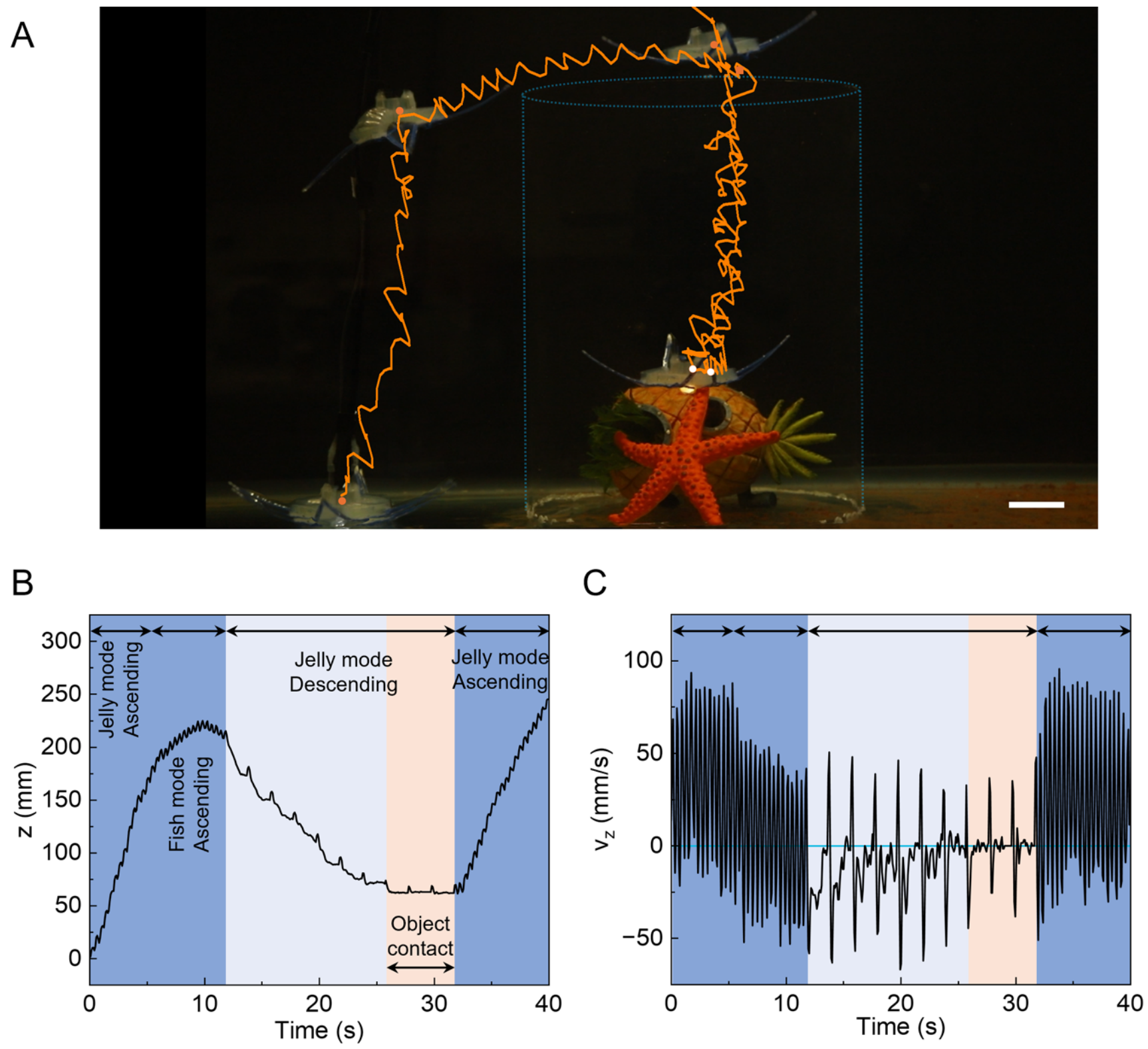

Figure S32. (A) Corresponding experimental trajectories of the soft swimmer integrated with an underwater camera. (B and C) Measured planar trajectories and the corresponding displacement and velocity along the *z*-axis as functions of time for the case shown in Figure 5H. Scale bar: 20 mm.

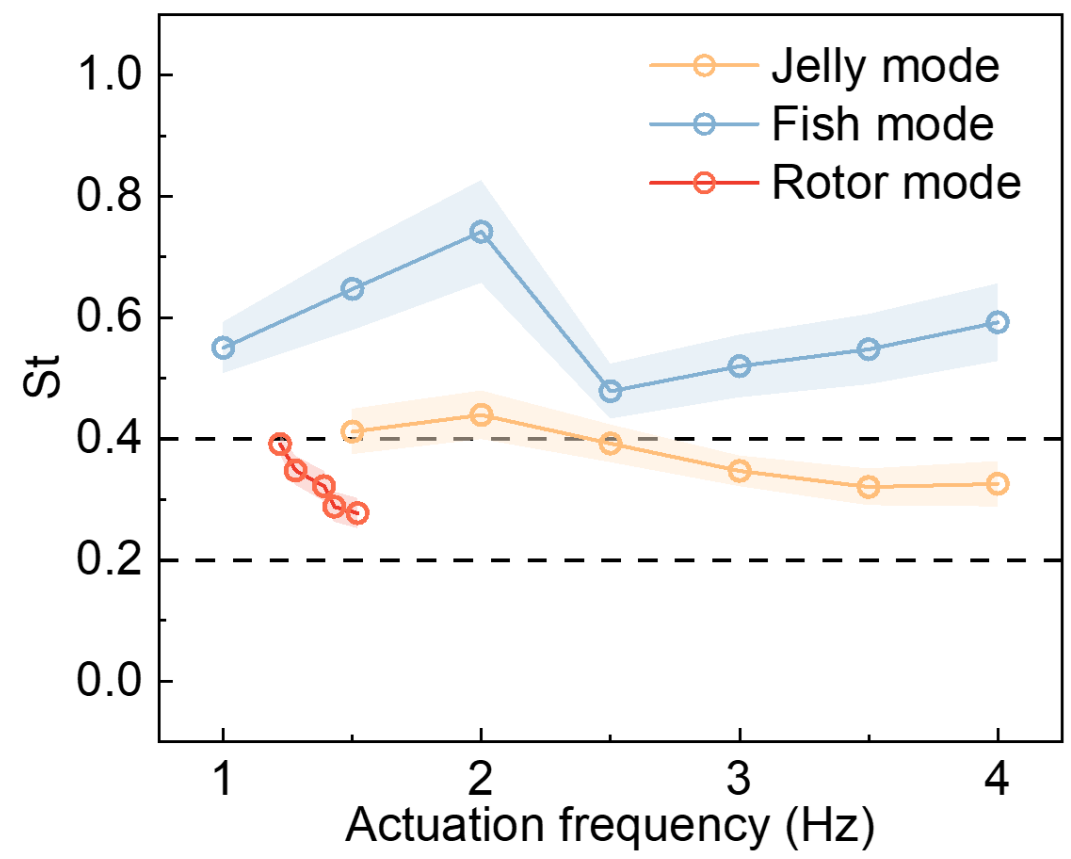

Fig. S33. Parametric study on the swimming performance of the soft swimmers with varying actuation frequency f characterized by the St.

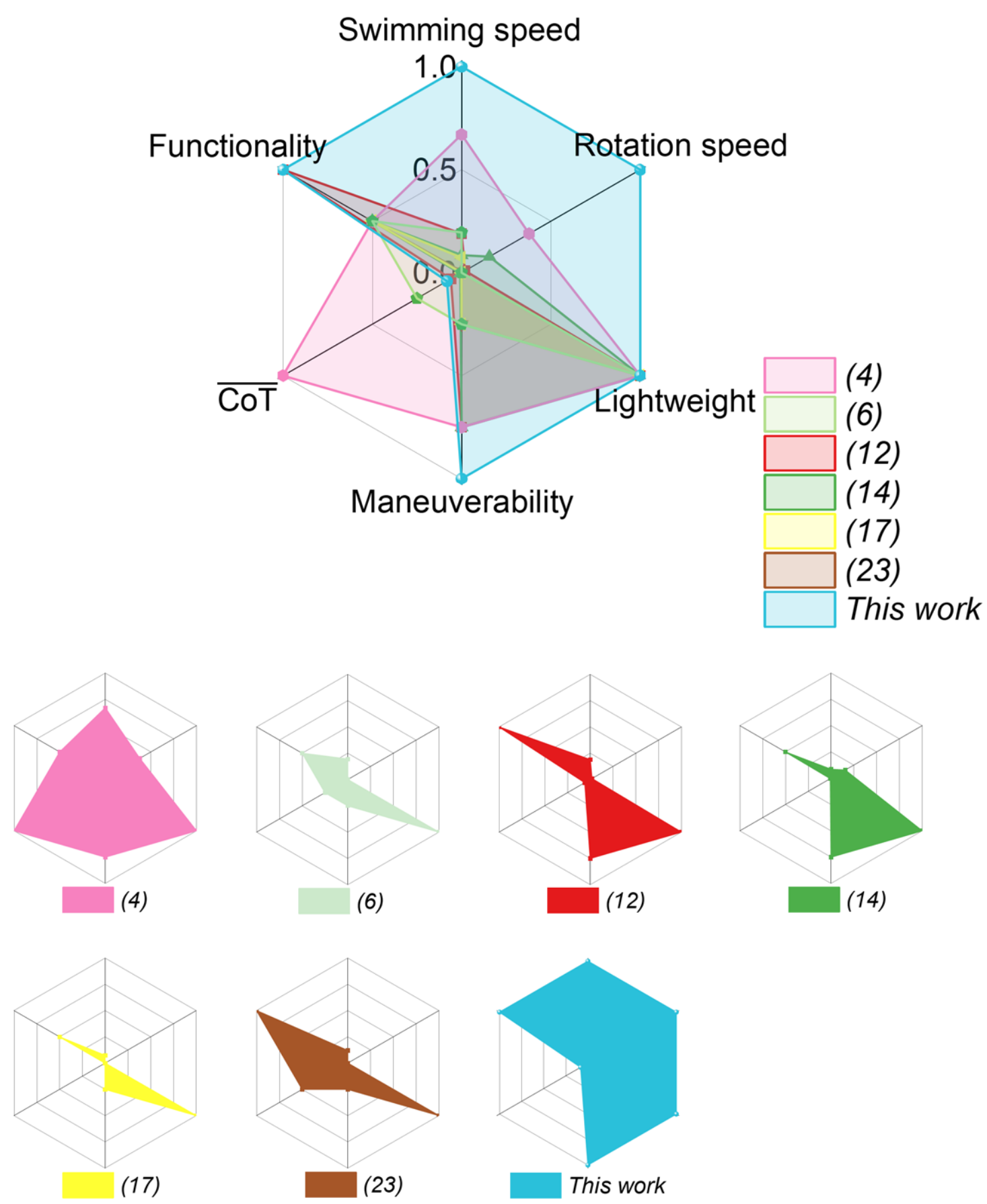

Figure S34. Radar chart comparing the performance of the feather star-inspired soft swimmer with state-of-the-art jellyfish-inspired soft swimming robots in metrics including swimming speed, rotation speed, maneuverability, lightweight, functionality, and actuation efficiency.

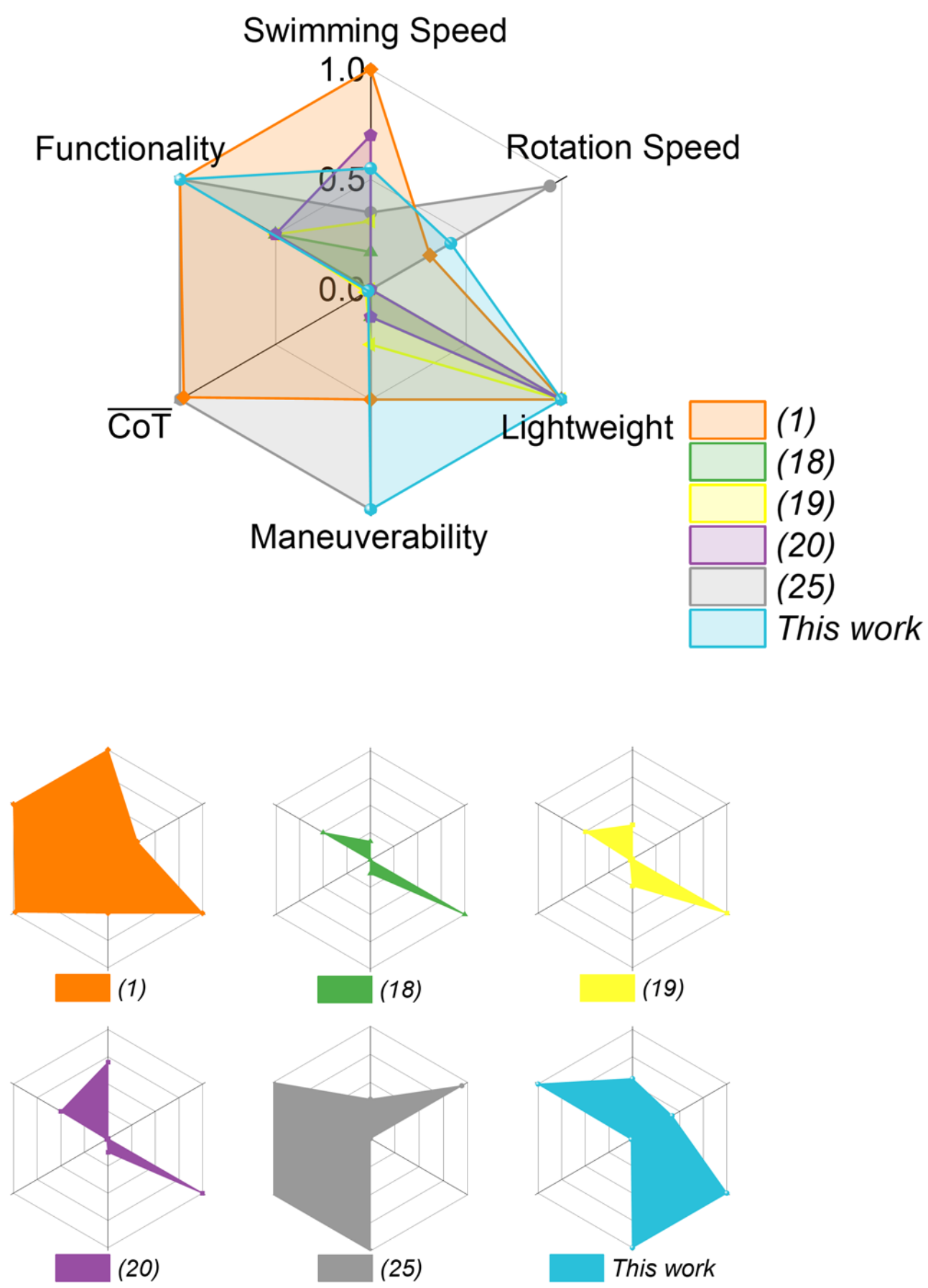

Figure S35. Radar chart comparing the performance of the feather star-inspired soft swimmer with state-of-the-art fish-inspired soft swimming robots in metrics including swimming speed, rotation speed, maneuverability, lightweight, functionality, and actuation efficiency.

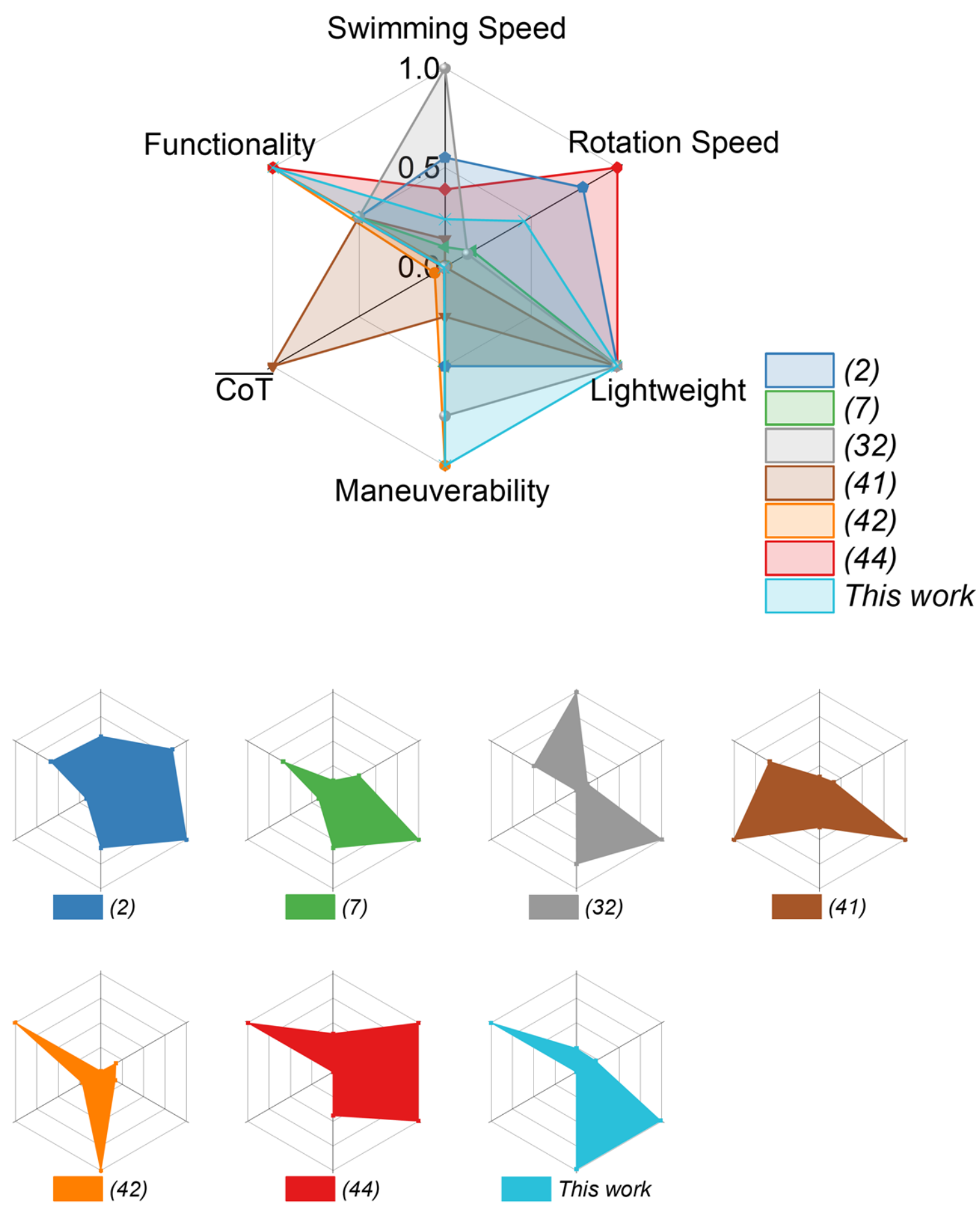

Figure S36. Radar chart comparing the performance of the feather star-inspired soft swimmer with state-of-the-art other bio-inspired soft swimming robots in metrics including swimming speed, rotation speed, maneuverability, lightweight, functionality, and actuation efficiency.

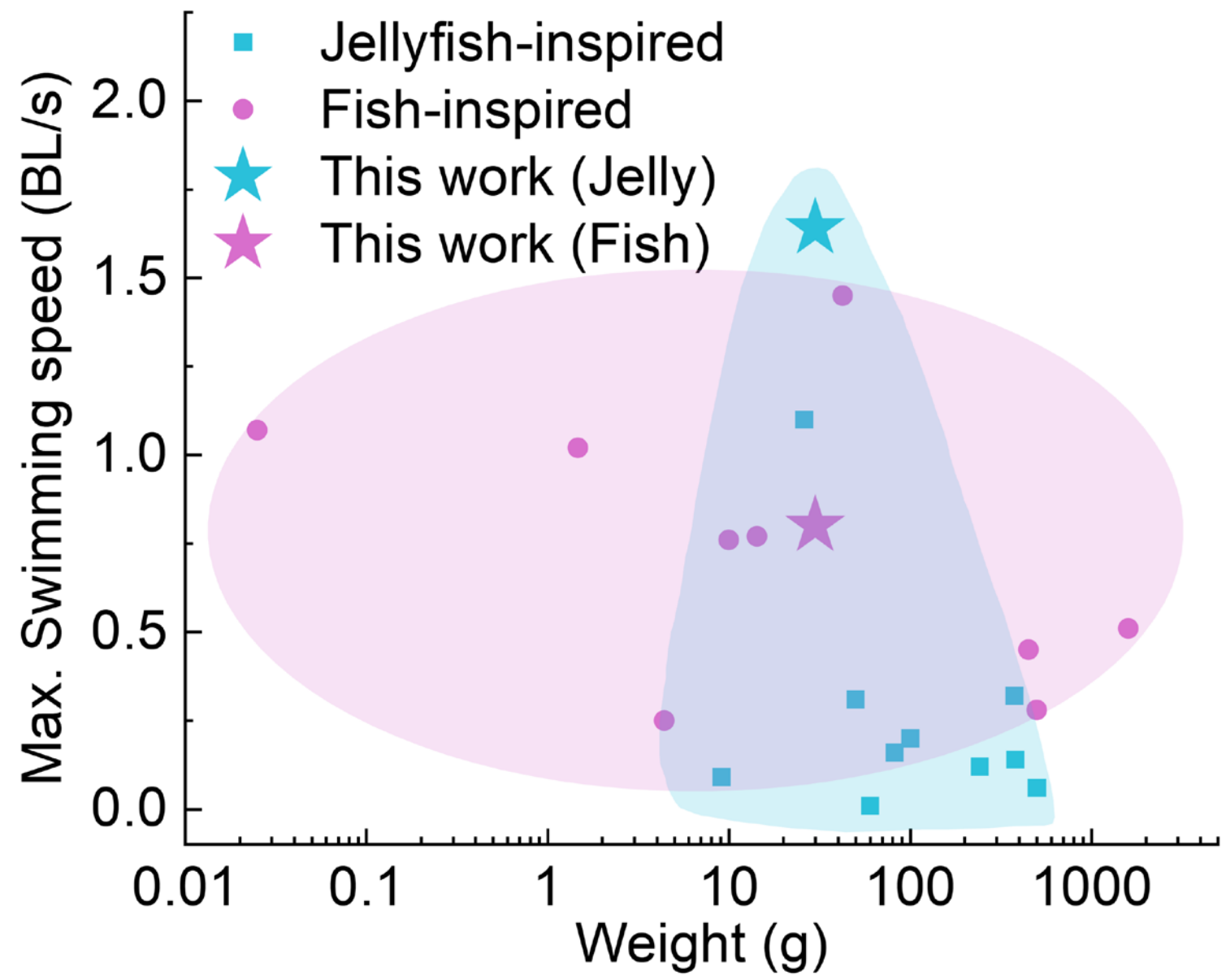

Figure S37. Relative speed comparison between this work (jellyfish- and fish-like modes) and other jellyfish- and fish-inspired soft swimming robots.

Table S1.

Category		Max. speed (BH/s)	Body length (mm)	Max. speed (mm/s)	Rotation speed (°/s)	Weight (g)	CoT	Maneuverability	Power input (N)	Actuation	Functionality
**Jellyfish-inspired soft robots**	Soft robotic jellyfish *(4)*	0.14	210	30	~14	380	200.88	2D	2	Pneumatic	Swimming only
	Soft jellyfish robot *(5)*	0.09	71	6.3	Unknown	9.1	unknown	2D	2	DEA	Swimming only
	Ocyropsis-inspired *(6)*	1.1	90	100	34	26	1.45	2D	4	HASEL	Swimming only
	Robojelly *(12)*	0.12	164	20	-	242	2270	1D	7	SMA	Swimming only
	Soft jellyfish robot *(13)*	0.06	140	9	-	~500	unknown	1D	1	DEA	Swimming only
	Jellyfish-inspired *(14)*	0.01 (TT); 0.02 (UTT)	160	2 (TT); 3 (UTT)	-	60 (TT); 230 (UTT)	2525.25	1D	1	DEA	Swimming only
	Biomimetic robotic jellyfish *(15)*	0.2	30	6	-	unknown	unknown	1D	1	Electromagnetic (Liquid metal)	Underwater monitoring
	Jelly-like underwater robot *(16)*	0.16 (TT); 0.3 (UTT)	30.3	4.9 (TT); 9.1 (UTT)	-	82 (TT); 137.4 (UTT)	unknown	1D	1	DEA	Swimming only
	Bistable jellyfish robot *(17)*	0.32 (TT); 0.38 (UTT)	118.8 (TT); 123.7 (UTT)	38 (TT); 47 (UTT)	-	unknown (TT); 377.3 (UTT)	5.76	1D	1	Pneumatic	Swimming only
	Jellyfish-like *(23)*	0.31 (TT); 0.16 (UTT)	160	50 (TT); 20 (UTT)	1.7	50 (TT); 170 (UTT)	24.14	2D	2	HASEL	Waste collection
This work		1.64 (J); 0.8 (F); 0.64 (R)	55	90 (J); 44 (F); 35 (R)	90 (R)	14.5	17.6 (J); 32.4 (F); 38.2 (R)	3D	2	Pneumatic	Waste collection; Underwater exploration; Underwater monitoring

Table S2.

Category		Max. speed (BL/s)	Body length (mm)	Max. speed (mm/s)	Rotational speed (°/s)	Weight (g)	CoT	Maneuverability	Power input (N)	Actuation	Functionality
**Fish-inspired soft robots**	Soft electronic fish *(1)*	1.45 (TT); 0.69 (UTT)	93	135 (TT); 64 (UTT)	7.2	42.5 (TT); 90.3 (UTT)	0.43	2D (Surface only)	2	DEA	Stealth sailing
	Breaststroke-inspired *(9)*	0.77	100	76.7	14.3	14.3	unknown	2D (Surface only)	2	DEA	Swimming only
	Soft robotic fish *(10)*	0.76	100	76	15.8	unknown	unknown	2D (Surface only)	2	DEA	Swimming only
	Swimming biohybrid fish *(11)*	1.07	14	15	18.2	0.025	unknown	2D (Surface only)	2	Phototactic	Swimming only
	Soft biomimetic fish *(18)*	0.25	150	37.2	unknown	4.4	573.5	1D (Surface only)	2	DEA	Swimming only
	Snailfish-inspired *(19)*	0.45	115	51.9	-	450	16.6	1D	1	DEA	Swimming only
	Flapping soft aquabot *(20)*	1.02	19.5	19.9	-	1.47	34.7	1D (Surface only)	1	Electrified hydrogel	Swimming only
	Mackerel-inspired *(21)*	0.28	125	35	-	unknown	unknown	1D (Surface only)	4	DEA	Swimming only
	SoFi *(25)*	0.51	470	239.7	22.1	1600	0.42	3D	6	Hydraulic	Underwater exploration
This work		1.64 (J); 0.8 (F); 0.64 (R)	55	90 (J); 44 (F); 35 (R)	90 (R)	14.5	17.6 (J); 32.4 (F); 38.2 (R)	3D	2	Pneumatic	Waste collection; Underwater exploration; Underwater monitoring

Table S3.

Category		Max. speed (BL/s)	Body length (mm)	Max. speed (mm/s)	Rotational speed (°/s)	Weight (g)	CoT	Maneuverability	Power input (N)	Actuation	Functionality
**Other bioinspired soft robots**	Bistable soft swimmer (2)	3.74	22.8	85.3	157	3.1	39	2D (Surface only)	4	Pneumatic	Swimming only
	Monostable soft swimmer (3)	6.8 (TT); 1.18 (UTT)	23 (TT); 30 (UTT)	156.4 (TT); 35.4 (UTT)	26	2.2 (TT); 4.4 (UTT)	23.7	2D	1	Pneumatic	Swimming only
	Sea slug-inspired (7)	0.69	160	110.4	30.8	31.8	68.3	2D (Surface only)	4	Pneumatic	Swimming only
	Soft-bodied manta (22)	0.67	150	100	-	unknown	unknown	1D (Surface only)	1	Pneumatic	Swimming only
	Turtle-like (39)	0.35	64.3	22.5	3.8	82.5	unknown	2D (Surface only)	4	SMA	Swimming only
	Eel-inspired (40)	0.009	73	1.9	-	unknown	unknown	1D	6	DEA	Stealth sailing
	Squid-inspired (41)	0.98	266	260	-	380	0.087	1D	1	Piston	Swimming only
	Octopus-inspired (42)	0.05	330	16.5	2.2	1320	1.44	3D	6	Tendon	Underwater grasping
	Snake-like (43)	0.07	1010	75	8	unknown	unknown	2D (Surface only)	6	Hydraulic	Swimming only
	Flat swimming robot (44)	2.64 (TT); 1.14 (UTT)	45	119 (UTT); 51.4 (TT)	120 (R; TT) 195 (R; UTT)	1.25 (TT); 6.25 (UTT)	13	2D (Surface only)	2	HASEL	Navigation; Object translation
This work		1.64 (J); 0.8 (F); 0.64 (R)	55	90 (J); 44 (F); 35 (R)	90 (R)	14.5	17.6 (J); 32.4 (F); 38.2 (R)	3D	2	Pneumatic	Waste collection; Underwater exploration; Underwater monitoring

Supplementary movies

Movie S1. Schematic design and fabrication of the feather star–inspired soft robot.

Movie S2. Measured force-normalized displacement (F-$\bar{u}$) tests and corresponding energy landscape (U-$\bar{u}$).

Movie S3. Stationary swimming of the feather star–inspired soft robot in three modes.

Movie S4. Free swimming of feather star–inspired soft robot in Jelly Mode.

Movie S5. Free steering of feather star–inspired soft robot in Fish Mode.

Movie S6. 3D particle image velocimetry tests for Jelly Mode.

Movie S7. 3D wake formations for Fish Mode (particle image velocimetry and computational fluid dynamics).

Movie S8. Free swimming of feather star–inspired soft robot in Rotor Mode (clockwise).

Movie S9. Vorticity field in Rotor Mode (computational fluid dynamics).

Movie S10. Continuous transitions between Jelly and Fish Modes of feather star–inspired soft robot.

Movie S11. 3D trajectory of feather star–inspired soft robot in combined modes.

Movie S12. Underwater waste collections.

Movie S13. Object lifting via cooperative operation mode.

Movie S14. Underwater exploration and monitoring.
